# Small extracellular vesicle-mediated epithelial–macrophage crosstalk contributes to macrophage dysfunction during PEDV infection

**DOI:** 10.1038/s41467-026-77854-w

**Published:** 2026-09-18

**Authors:** Qiu Zhong, Chengcheng Wang, Ruiling Liu, Yunlei Cao, Hui Zeng, Na Su, Rongfeng Tang, Wenqian Wang, Jiaxin Qi, Yuchen Li, Qian Yang

**Affiliations:** MOE Joint International Research Laboratory of Animal Health and Food Safety, College of Veterinary Medicine, Nanjing Agricultural University, Nanjing, PR China

**Keywords:** Actin, Viral immune evasion, Virus-host interactions

## Abstract

Mucosal immune defense depends on coordinated communication between epithelial and immune cells, yet pathogens can disrupt this crosstalk through diverse mechanisms. Here we show that porcine epidemic diarrhea virus (PEDV), an enteric alphacoronavirus, reshapes epithelial small extracellular vesicle (sEV) signaling to impair macrophage function. Despite detection by the intestinal innate immune system, PEDV promotes the release of epithelial sEVs enriched in immunoregulatory noncoding RNAs. Among this cargo, miR-190a targets the actin-regulating protein TMOD3 and disrupts F-actin remodeling, reducing macrophage migration. In parallel, the long noncoding RNA TS2083 promotes annexin A2 degradation, impairing phagocytic cup formation and suppressing phagocytosis. Moreover, serum sEVs from PEDV-infected piglets are enriched in both RNAs and impair isolated alveolar macrophage migration and phagocytosis, suggesting distal macrophage modulation. Collectively, these findings implicate sEV-mediated macrophage reprogramming in PEDV-mediated immune evasion and highlight sEV-associated RNAs as potential biomarkers of PEDV infection and therapeutic targets for restoring macrophage-mediated mucosal immunity.

## Abstract (web summary)

Here, Li and colleagues show that porcine epidemic diarrhea virus alters the RNA cargo of small extracellular vesicles from intestinal epithelial cells. These vesicles weaken intestinal immune defence by impairing macrophage migration and phagocytosis.

## Introduction

The recurrent emergence of viral pathogens such as influenza viruses and coronaviruses has defined recent decades, imposing persistent threats to global health and major economic burdens^1^. Although advances in vaccination, interferon-based therapies, and antivirals have reduced viral morbidity and mortality, persistent problems such as delayed clearance, prolonged shedding, and secondary infections continue to challenge clinical management^2,3^. Accumulating clinical and experimental evidence suggests that these shortcomings are driven by the ability of viruses to evade immune recognition and suppress mucosal immunity at the earliest stages of infection^4,5^. Dissecting the mechanisms of mucosal immune suppression is therefore critical not only for elucidating the molecular basis of viral immune evasion and its clinical consequences, but also for overcoming current bottlenecks and guiding the development of next-generation antiviral strategies.

Viral infection typically begins at the respiratory or intestinal mucosal epithelium. As the first line of defense, epithelial cells detect pathogen-associated molecular patterns (PAMPs) through pattern recognition receptors (PRRs) such as RIG-I–like receptors and Toll-like receptors, initiating signaling cascades that induce type I/III interferons and pro-inflammatory mediators. These responses establish local gradients that recruit lymphocytes, macrophages, and dendritic cells to clear infection and prime adaptive immunity. Consequently, studies of mucosal immune suppression have traditionally centered on interferon and inflammatory pathways. Yet accumulating evidence indicates that activation of these canonical responses alone is insufficient for effective control. For example, SARS-CoV-2 replicates extensively in the upper respiratory tract despite interferon induction^6^, and mucosal SIV exposure elicits strong IFN-α/β responses but still results in systemic dissemination^7^. Together, these findings underscore that while interferon and inflammatory signaling are indispensable, they do not fully capture the defensive capacity of mucosal immunity.

The basis of this discrepancy lies in the mucosa itself, a complex barrier formed by multiple cell lineages whose immune status is determined not by single cells or isolated pathways, but through dynamic epithelial–immune interactions^8,9^. Evidence shows that while viruses activate innate responses at mucosal sites, they often reprogram host signaling to induce immunosuppressive mediators, reshaping the microenvironment to favor dissemination and persistence. For example, HIV-1 Tat upregulates PD-L1 on dendritic cells to weaken T-cell responses^10^; HCV core induces monocyte reprogramming into an MDSC-like phenotype that generates NOX2-dependent ROS to suppress T-cell activity^11^; and influenza A virus enhances glycolysis and lactate accumulation, causing local acidification and direct inhibition of effector T cells^12^. Together, these studies show that mucosal immune suppression arises not from a single signaling defect but from the coordinated reprogramming of multiple cell types. Future work must therefore move beyond cell- or pathway-centric views and focus on the dynamic interplay within multicellular networks to reveal how viruses achieve mucosal immune suppression and immune evasion.

As an enteric porcine coronavirus belonging to the genus *Alphacoronavirus*, porcine epidemic diarrhea virus (PEDV) has caused devastating losses to the global swine industry since its re-emergence in the 2010s^13^. A major obstacle to PEDV control is its age-dependent pathogenicity: it causes acute, almost invariably fatal enteric disease in neonatal piglets, but establishes subclinical infection with prolonged shedding in older animals, thereby sustaining transmission^14,15^. These distinct outcomes have been closely linked to virus-induced intestinal immune suppression, yet most mechanistic studies have relied on heterologous in vitro systems and focused on peak viral replication, failing to capture the dynamic immune networks operating during infection in vivo. Piglet models show that within 12–24 h after oral challenge, intestinal levels of IL-6, TNF-α, and type I/III interferons rise sharply, accompanied by activation of the JAK–STAT pathway and numerous interferon-stimulated genes (ISGs)^16^. Yet these early antiviral and inflammatory responses are insufficient to control viral replication, enabling PEDV to spread rapidly across the mucosal epithelium and underscoring the complexity of its immunosuppressive mechanisms.

In this study, we combined an in vivo piglet infection model with single-cell RNA sequencing (scRNA-seq) to dissect the mucosal immune response to PEDV. We demonstrate that PEDV robustly activates interferon and inflammatory signaling in intestinal epithelial cells and enhances their communication with mucosal immune populations, whereas lamina propria immune cells—particularly macrophages—show marked suppression of effector functions, including migration, phagocytosis, and antigen presentation. Moreover, PEDV infection promotes the release of host-derived sEVs from intestinal epithelial cells, which deliver immunoregulatory cargos to macrophages and contribute to impaired macrophage migration and phagocytosis. Together, our findings support a role for PEDV-induced epithelial sEVs as vesicular mediators of mucosal immune dysfunction during intestinal infection. By linking sEV-associated noncoding RNA cargos to impaired macrophage migration and phagocytosis, this study provides insight into the immunological basis of PEDV mucosal pathogenesis and highlights candidate vesicle-associated host pathways for future studies aimed at restoring macrophage-mediated mucosal immunity.

## Results

### PEDV infection impairs intestinal macrophage functions in neonatal piglets

**Fig. 1:**
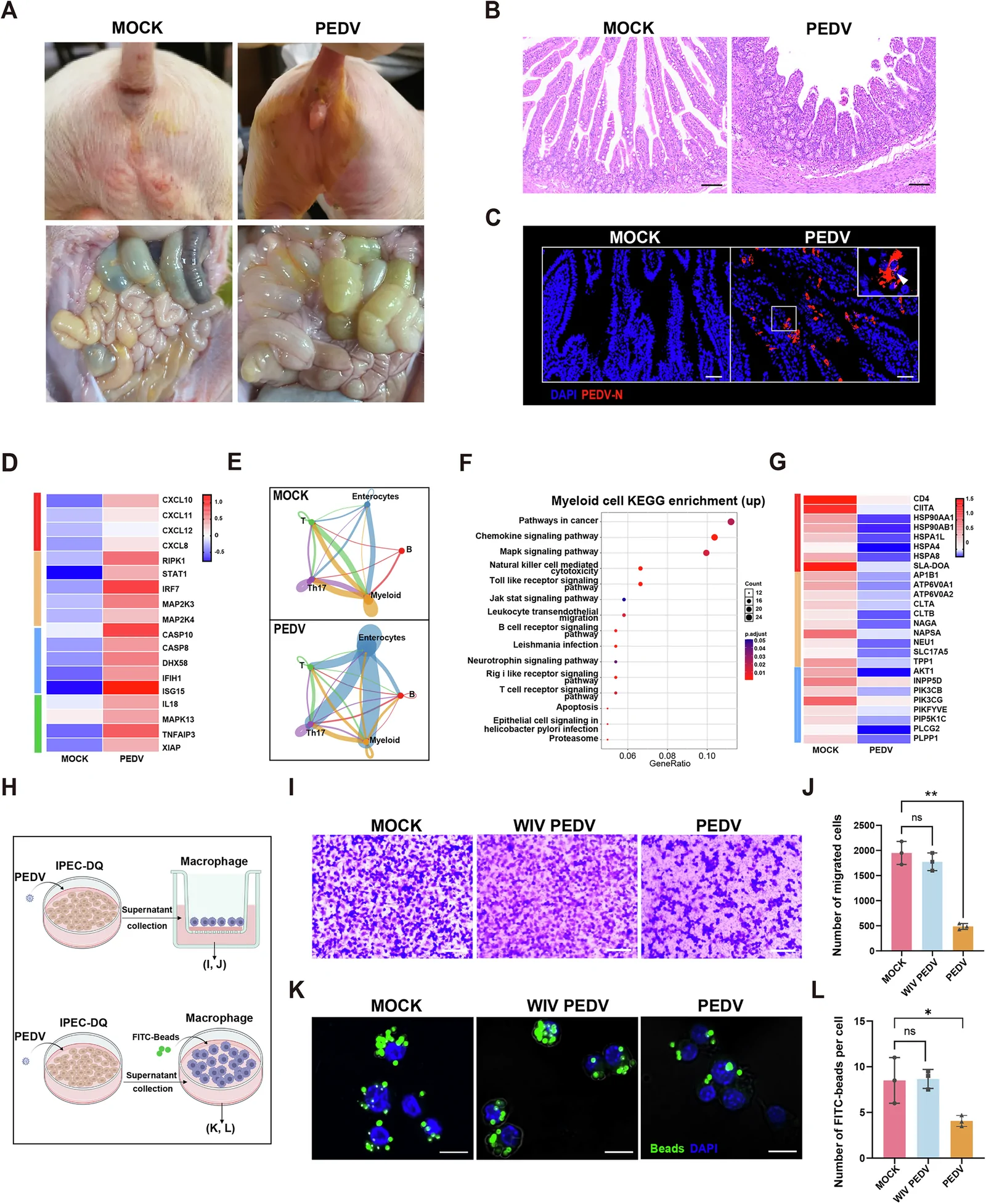
Single-cell transcriptomics reveal intestinal mucosal immune suppression after PEDV infection. **A** Gross pathological changes of intestinal tissues after PEDV infection. **B** H&E staining of jejunal tissues showing villous atrophy and epithelial shedding. Scale bar, 100 μm. The images shown were representative of three independent replicates. **C** Confocal imaging of jejunal sections showing PEDV antigen (red) detected by anti-PEDV monoclonal antibody; nuclei were counterstained with DAPI (blue). Scale bar, 50 μm. White arrows indicate PEDV-positive cells. The images shown were representative of three independent replicates. **D** Transcriptional profiles of epithelial cells showing chemokine receptor (red), TLR (orange), RIG-I signaling (blue), and NOD-like receptor (green) modules. **E** Ligand–receptor interaction analysis between epithelial and myeloid subsets (macrophages, DCs, monocytes). **F** KEGG enrichment of upregulated genes in intestinal myeloid cells following PEDV infection. Dot size indicates gene counts, and color indicates adjusted *P* values. **G** Transcriptional profiles of myeloid cells showing antigen presentation (red), lysosome (orange), and phagosome (blue) pathways. **H**–**L** Functional validation of total conditioned supernatants from MOCK-, WIV PEDV-, or PEDV-treated epithelial cells on macrophage migration and phagocytosis. **H** Experimental design. At 24 h, conditioned media were harvested, neutralized with anti-PEDV serum where indicated, and applied either to the lower chamber of Transwells for migration assays or directly to macrophages for phagocytosis assays with fluorescent beads. **I** Representative images of migrated macrophages in Transwell assays. Scale bar, 50 μm. **J** Quantification of migrated macrophages shown in (**I**, *n* = 3 biologically independent samples). **K** Confocal microscopy of macrophages associated with fluorescent beads; nuclei were counterstained with DAPI. Scale bar, 10 μm. **L** Quantification of bead uptake shown in (K, *n* = 3 biologically independent samples). Data are presented as mean ± SD from *n* = 3 independent experiments. Each dot represents one independent experiment. Statistical significance was determined by one-way ANOVA with Tukey’s post-hoc test, with *P*-values denoted as follows: *P < 0.05; **P < 0.01. Exact *P* values are available in the source data. Schematic in (**H**): Created in BioRender. Qin, X. (2026) https://BioRender.com/u2xdpiu.

To evaluate the impact of PEDV on intestinal immunity, we established an oral infection model in neonatal piglets. By 36 h post-infection, piglets developed acute watery diarrhea with extensive perianal soiling. Necropsy revealed dilated small intestines with thinned translucent walls and yellow-green fluid contents (Fig. 1A). Histopathology showed severe villous atrophy, brush border damage, and widespread epithelial shedding, while immunofluorescence confirmed abundant PEDV antigen in villous epithelial cells (Fig. 1B, C). scRNA-seq of jejunal mucosa at 36 h identified 29 transcriptionally distinct cell subsets spanning epithelial, secretory, mesenchymal, and immune compartments (Fig. S1A). Cell-composition and ROE summaries suggested condition-associated shifts after PEDV infection, including lower relative representation of epithelial and secretory lineages and T cells, together with relative enrichment of myeloid populations (Fig. S1B-E). Pathway enrichment revealed epithelial enrichment of tight junction, adherens junction, actin cytoskeleton, focal adhesion, and MAPK/TGF-β signaling pathways (Fig. S1F), consistent with epithelial barrier disruption, aberrant differentiation, and fibrotic remodeling. PRR-related pathways (TLR, RLR and NLR) were enriched, with increased IFN-α, IFN-γ, and TNF-α transcripts (Fig. 1D, Fig. S1G), indicating initiation of systemic antiviral inflammation.

CellChat analysis demonstrated enhanced epithelial–immune interactions, especially with myeloid cells (Fig. 1E). Myeloid enrichment showed enrichment of innate signaling (RLR, TNF/NF-κB and chemokines) (Fig. 1F), together with lower activity of macrophage effector pathways, including lysosome, phagosome, autophagy, endocytosis, and Fcγ receptor–mediated phagocytosis (Fig. S1H). Downregulated transcripts included lysosomal acidification/hydrolases (ATP6V0A1, ATP6V0A2, NAGA, NAPSA and NEU1), endocytosis (CLTA and CLTB), and antigen presentation (CIITA and SLA-DOA) (Fig. 1G), consistent with reduced transcriptional programs related to phagocytosis and antigen presentation. Sample-aware pseudobulk analysis using individual piglet libraries as aggregation units recapitulated the major directions observed in the single-cell analysis, with increased epithelial antiviral/inflammatory programs and reduced macrophage effector programs at the sample level (Fig. S2A-F).

To assess the cross-strain consistency of these transcriptional trends, we re-analyzed published scRNA-seq data from piglets infected with another highly pathogenic PEDV strain^17^. This dataset showed concordant epithelial PRR activation and epithelial–immune interaction patterns, together with reduced macrophage effector programs, including lysosome, phagosome, autophagy, and Fcγ receptor-mediated phagocytosis pathways (Fig. S2G–I). Together, the sample-aware pseudobulk analysis and the public dataset reanalysis supported the major transcriptional trends identified in our in-house scRNA-seq dataset.

### PEDV-induced intestinal epithelial sEVs suppress macrophage migration and phagocytosis

ScRNA-seq revealed that PEDV infection increased epithelial immune responses in the intestine, with marked induction of inflammatory cytokines and chemokines. In contrast, although macrophages initiated innate responses, their effector functions were suppressed. To validate these in vivo findings, we analyzed transcriptomic datasets from in vitro infection models using multiple PEDV strains^18,19^ (PRJNA599091; SRR14552484 and SRR14552504). Consistent with the in vivo results, transcriptomic datasets from porcine jejunal epithelial cells infected with different PEDV strains likewise showed increased cytokine and chemokine expression (Fig. S3A). Based on this, we established an IPEC-DQ–3D4/21 coculture system. PEDV replicated efficiently in IPEC-DQ cells, reaching peak titers at 24 h post-infection and inducing cytopathic effects by 48 h (Fig. S3B-E). In line with single-cell results, PEDV-infected IPEC-DQ cells secreted high levels of IL-1β, IL-6, and TNF-α, and their conditioned supernatants reduced migration by 75.1% and phagocytosis by 52.3% (Fig. 1H–L). Population-level flow-cytometric analysis further confirmed these functional changes: PEDV-conditioned medium reduced the number of migrated macrophages recovered from the lower Transwell chamber and decreased the proportion of bead-positive macrophages after fluorescent bead exposure (Fig. S3F, G). These inhibitory effects were absent when using supernatants from UV-inactivated PEDV (WIV PEDV)-treated cells. This epithelial secretome, therefore, combined inflammatory cues with macrophage-suppressive activity. In conditioned medium from PEDV-infected epithelial cells, IL-6 and TNF-α were increased, whereas PGE2 was not significantly elevated (Fig. S4A, B). Macrophages exposed to PEDV-conditioned medium also showed enrichment of IL-6/JAK/STAT3 and TNF-α/NF-κB transcriptional signatures (Fig. S4C, D), indicating that inflammatory activation and effector suppression coexisted in recipient macrophages. However, neutralization of IL-6 or TNF-α using neutralizing antibodies did not substantially restore macrophage migration or bead uptake (Fig. S4E, F), suggesting that these major soluble inflammatory cytokines were insufficient to account for the migration and phagocytosis defects induced by PEDV-conditioned medium.

**Fig. 2:**
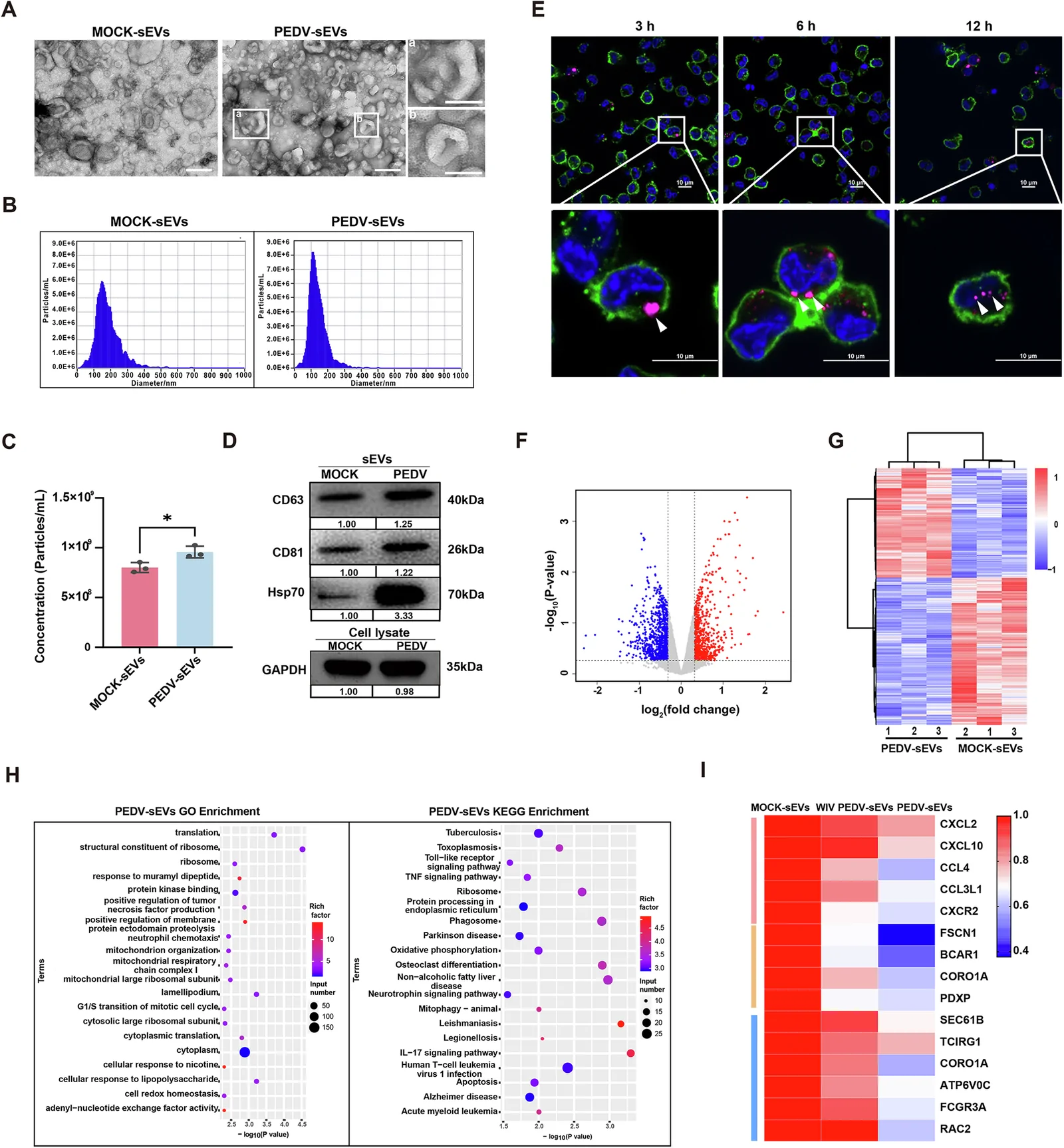
PEDV-induced epithelial sEVs reprogram macrophage transcriptional profiles. Characterization of sEVs isolated from IPEC-DQ cell supernatants by ultracentrifugation followed by anti-CD63 immunomagnetic enrichment. **A** Transmission electron microscopy showing typical cup-shaped morphology of sEVs. Insets (a′, b′) display higher-magnification views of boxed regions. Scale bars, 200 nm (overview) and 50 nm (insets). **B** Representative NTA particle size-distribution profiles showing the concentration of particles across different size ranges in MOCK-sEV and PEDV-sEV preparations. **C** Quantification of the peak particle concentration within the predominant size range (100–200 nm) from independent sEV preparations. For quantitative sEV measurements, each dot represents one independent sEV preparation. Data are presented as mean ± SD from *n* = 3 independent experiments. Each dot represents one independent experiment. Statistical comparisons between two groups were performed using two-tailed unpaired *t*-test. *P < 0.05. Exact *P* values are available in the source data. **D** Western blot analysis of sEV-associated markers in sEV preparations recovered from equal volumes of conditioned medium from MOCK- or PEDV-treated IPEC-DQ cells. CD63, CD81, and HSP70 were detected as sEV-associated markers. GAPDH was detected in matched donor-cell lysates to indicate comparable donor-cell input for sEV collection. The images shown were representative of three independent replicates. **E** Confocal microscopy of macrophages incubated with CM-DiI–labeled sEVs (red). F-actin is stained with FITC-phalloidin (green) and nuclei with DAPI (blue). White arrows indicate internalized sEVs. Scale bars, 10 μm. The images shown were representative of three independent replicates. Transcriptomic profiling of macrophages exposed to PEDV-induced sEVs. **F** Volcano plot of differentially expressed genes identified using edgeR v3.12.1 (red, upregulated; blue, downregulated; gray, not significant). Differential expression was assessed using a two-sided exact test. Genes with an absolute log₂(fold change) greater than 0.32 and a nominal *P* value of less than 0.05 were considered differentially expressed. *P* values were not adjusted for multiple comparisons. **G** Heatmap of hierarchical clustering of gene expression profiles. Each column represents an independent replicate of PEDV-sEVs or MOCK-sEVs treatment (*n* = 3). **H** GO and KEGG enrichment analyses of downregulated genes in macrophages exposed to PEDV-sEVs. Enrichment was assessed using a one-sided hypergeometric test. Terms with a nominal *P* value of less than 0.05 were considered enriched. *P* values were not adjusted for multiple comparisons. Dot size indicates the number of input genes, color indicates the rich factor, and the x-axis shows −log₁₀ (*P* value). **I** Heatmap of transcriptional profiles showing representative genes involved in macrophage chemotaxis, lamellipodia formation, and phagosome signaling pathways after treatment with PEDV-sEVs and WIV PEDV-sEVs.

**Fig. 3:**
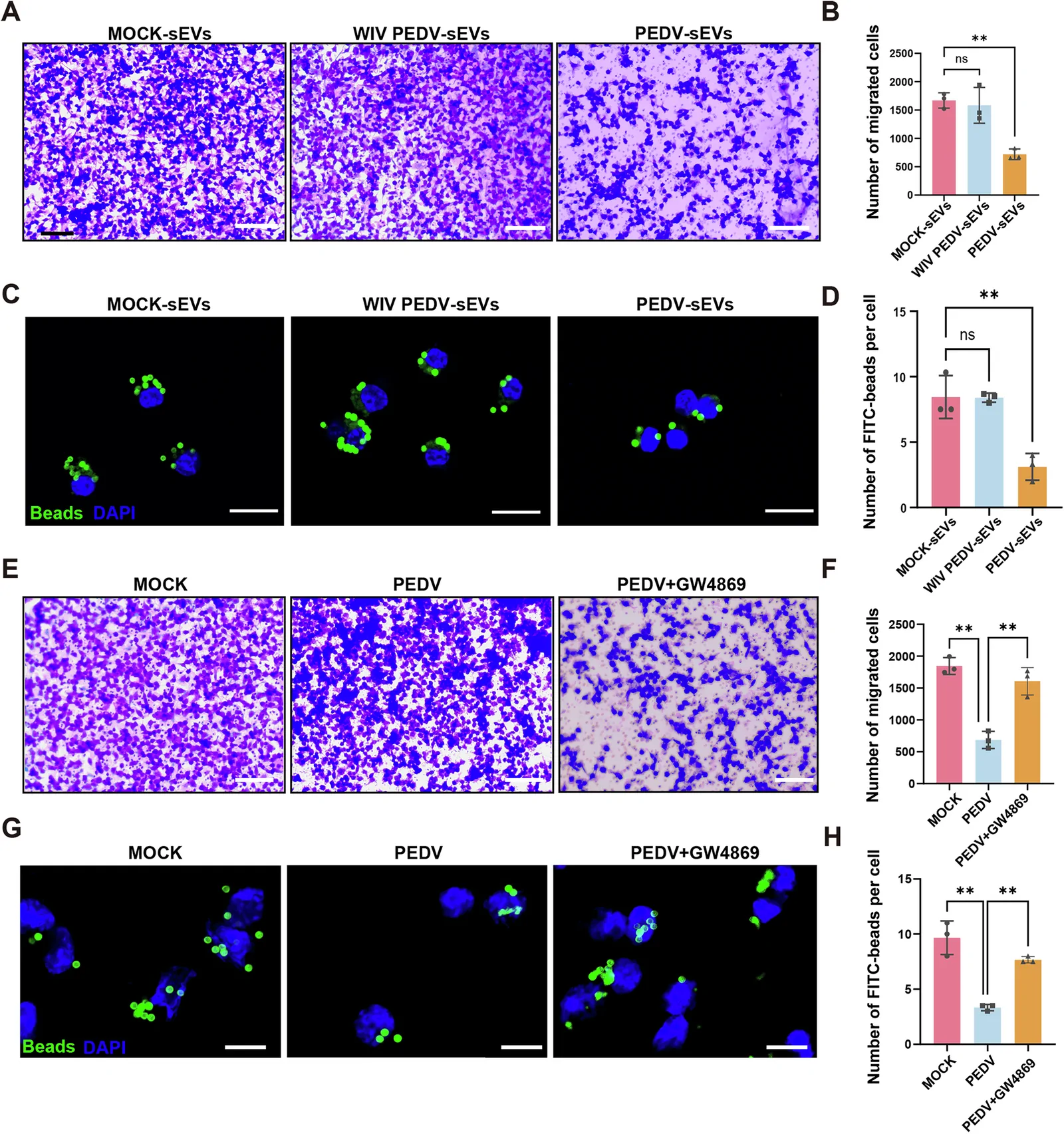
PEDV-induced epithelial sEVs suppress macrophage migration and phagocytosis. Macrophages were incubated for 24 h with BCA-normalized sEV preparations derived from MOCK-, WIV PEDV-, or PEDV-treated epithelial cells. sEV preparations were isolated from conditioned medium by differential ultracentrifugation followed by anti-CD63 immunomagnetic enrichment and are hereafter referred to as CD63-enriched sEV preparations. **A** Representative images of migrated macrophages in Transwell assays. Scale bar, 50 μm. **B** Quantification of migrated macrophages in (**A**, *n* = 3 biologically independent samples). **C** Confocal microscopy of macrophages engulfing fluorescent beads (green); nuclei were counterstained with DAPI (blue). Representative internalized beads are indicated by white arrows. Scale bar, 10 μm. **D** Quantification of bead uptake per macrophage shown in (**C**, *n* = 3 biologically independent samples). IPEC-DQ cells were inoculated with PEDV and treated with the sEVs biogenesis inhibitor GW4869. Supernatants were collected and incubated with macrophages. **E** Representative images of migrated macrophages in Transwell assays. Scale bar, 50 μm. **F** Quantification of migrated macrophages in (**E**, *n* = 3 biologically independent samples). **G** Confocal microscopy of macrophages engulfing fluorescent beads (green); nuclei were counterstained with DAPI (blue). White arrows highlight representative internalized beads. Scale bar, 10 μm. **H** Quantification of bead uptake per macrophage shown in (**G**, *n* = 3 biologically independent samples). Data are presented as mean ± SD from *n* = 3 independent experiments. Each dot represents one independent experiment. Statistical significance was determined by one-way ANOVA with Tukey’s post-hoc test, with *P*-values denoted as follows: *P < 0.05, **P < 0.01. Exact *P* values are available in the source data.

Further analysis showed that PEDV infection increased the release of small extracellular vesicles (sEVs) from intestinal epithelial cells. Ultracentrifugation yielded visible vesicle pellets mainly in the infected group (Fig. S5A). TEM revealed typical cup-shaped vesicles (Fig. 2A), and nanoparticle tracking analysis (NTA) showed a significant increase in particle concentration, with size distributions peaking at 100–120 nm (Fig. 2B, C). Western blotting detected the sEV-associated markers CD63, CD81, and HSP70 in isolated sEV-enriched preparations, whereas calnexin was detected in donor-cell lysates but not in sEV preparations, suggesting minimal contamination by endoplasmic reticulum-derived cellular components. Under equal-volume loading, PEDV-sEVs showed stronger CD63, CD81, and HSP70 signals than MOCK-sEVs (Fig. 2D). In contrast, when equal amounts of sEV protein were loaded, CD63, CD81, and HSP70 signals were comparable between MOCK-sEVs and PEDV-sEVs (Fig. S5B). For downstream macrophage treatment experiments, MOCK-sEVs, WIV PEDV-sEVs, and PEDV-sEVs were quantified by BCA assay and applied at equal total protein amounts from sEV preparations, enabling comparison of their functional activity under normalized sEV-input conditions. Confocal microscopy revealed rapid, time-dependent uptake of PEDV-sEVs by macrophages, with detectable signals within 3 h (Fig. 2E). Under these normalized sEV-input conditions, transcriptomic profiling of sEV-treated macrophages, identified 1,547 differentially expressed genes in the PEDV-sEV group (Fig. 2F, G). GO and KEGG analyses highlighted suppression of migration- and phagocytosis-related pathways, including chemokine signaling, lamellipodia formation, and phagosome function (Fig. 2H). Pathway-level comparison showed that WIV PEDV-sEVs induced weaker transcriptional effects than PEDV-sEVs (Fig. 2I and Fig. S5C). Before functional assays, sEV preparations from MOCK-, WIV PEDV-, and PEDV-treated epithelial cells were quantified by BCA assay and normalized to the same total protein input. Using these normalized sEV preparations, PEDV-sEVs reduced macrophage migration (Fig. 3A, B, Fig. S5D) and bead phagocytosis (Fig. 3C, D, Fig. S5E), whereas sEVs from WIV PEDV-treated cells had no comparable effect. To determine whether this activity remained after removal of the vesicular fraction, we prepared sEV-depleted conditioned medium from MOCK-, WIV PEDV-, and PEDV-treated epithelial cells. Flow-cytometric analysis showed that sEV-depleted PEDV-conditioned medium did not reproduce the macrophage-suppressive phenotype, with migrated macrophage counts and bead-positive macrophage levels comparable to MOCK and WIV PEDV controls (Fig. S5F, G). Consistently, GW4869-mediated inhibition of sEV release from donor epithelial cells restored macrophage migration and bead uptake during PEDV infection (Fig. 3E–H, Fig. S5H, I). To further assess whether PEDV-induced epithelial sEVs could elicit similar macrophage functional responses beyond the porcine system, we performed a preliminary validation using THP-1-derived human macrophage-like cells. Consistent with the results obtained in porcine macrophages, PEDV-sEV treatment reduced THP-1-derived macrophage migration and bead uptake compared with MOCK-sEV treatment (Fig. S5J, K). These data suggest that macrophage responses to PEDV-induced epithelial sEVs may not be restricted to porcine macrophages, although the cross-species activity of individual sEV-associated ncRNA cargos remains to be further defined.

### sEV-associated miR-190a contributes to PEDV-induced suppression of macrophage migration

Noncoding RNAs, including miRNAs, lncRNAs, and circRNAs, comprise an important fraction of sEV-associated RNA cargos and play critical roles in intercellular communication^20^. To assess whether PEDV-induced sEVs regulate macrophage immunity through host RNA cargos, we performed RNA sequencing of sEVs released from PEDV-infected intestinal epithelial cells (Fig. S6A, B). PEDV infection remodeled sEV-associated ncRNA profiles, yielding 36 differentially expressed miRNAs (23 upregulated, 13 downregulated) and 41 differentially expressed lncRNAs (18 upregulated, 23 downregulated). By contrast, sEVs from WIV PEDV treated cells contained only 7 differentially expressed miRNAs and 7 differentially expressed lncRNAs (Fig. S6C–J), indicating that productive PEDV infection was associated with more extensive sEV-associated ncRNA remodeling. No viral RNA was detected, suggesting that the macrophage-regulatory effects were mainly associated with host-derived sEV RNA cargos rather than direct viral RNA transfer. These cargo differences provided the basis for subsequent miRNAs and lncRNAs functional analyses.

**Fig. 4:**
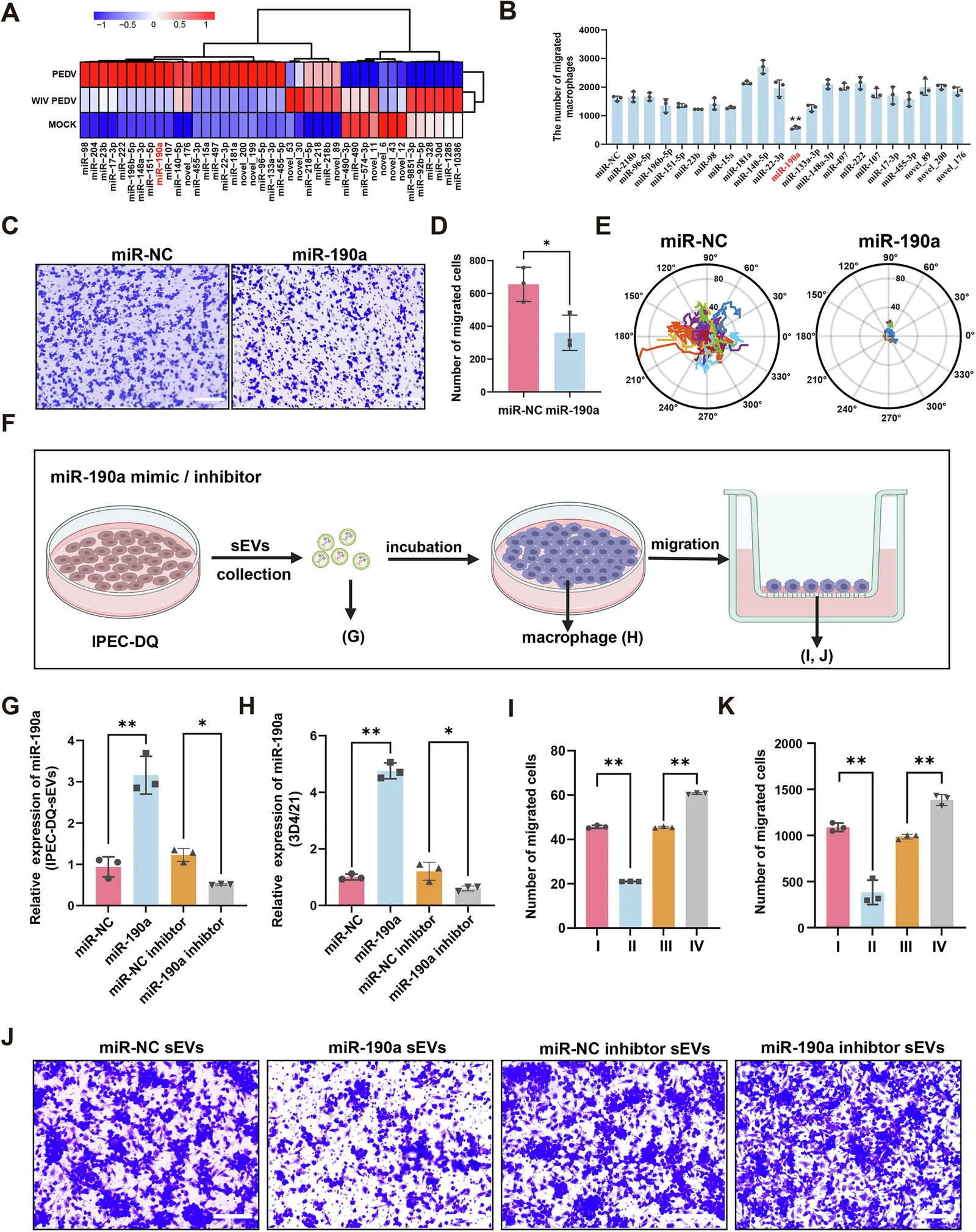
PEDV-induced epithelial sEVs suppress macrophage migration via miR-190a. **A** Heatmap of differentially expressed miRNAs in sEVs from PEDV-treated epithelial cells compared with MOCK and WIV PEDV controls. miR-190a is highlighted. **B** Transwell migration screen of macrophages transfected with candidate miRNA mimics. Each dot represents one independent experiment; bars indicate mean ± SD, *n* = 3 biologically independent samples. **C** Representative Transwell images showing reduced migration of macrophages transfected with synthetic miR-190a mimics. Scale bar, 50 μm. **D** Quantification of migrated macrophages shown in (**C**, *n* = 3 biologically independent samples). **E** Time-lapse microscopy tracking macrophage motility after transfection with miR-190a mimics. **F**–**K** Functional validation of sEV-associated miR-190a in regulating macrophage migration. **F** Schematic workflow: intestinal epithelial cells were transfected with miR-190a overexpression or inhibition constructs, sEVs were isolated and co-incubated with macrophages, and macrophage migration was assessed. **G**, **H** RT–qPCR analysis of miR-190a levels in sEVs and recipient macrophages (*n* = 3 biologically independent samples). **I** Flow-cytometric quantification of migrated macrophages (*n* = 3 biologically independent samples). Groups are defined as follows: I, miR-NC-sEVs; II, miR-190a-sEVs; III, inhibitor-NC-sEVs; IV, miR-190a-inhibitor-sEVs. **J** Representative Transwell images showing the effects of sEV-associated miR-190a on macrophage migration. Scale bar, 50 μm. **K** Quantification of migrated macrophages shown in (**J**, *n* = 3 biologically independent samples). Groups are defined as follows: I, miR-NC-sEVs; II, miR-190a-sEVs; III, inhibitor-NC-sEVs; IV, miR-190a-inhibitor-sEVs. Data are presented as mean ± SD from *n* = 3 independent experiments. Each dot represents one independent experiment. Statistical significance was determined using one-way ANOVA with Dunnett’s multiple comparisons test (**B**), a two-tailed unpaired *t*-test (**D**), and one-way ANOVA with Tukey’s multiple comparisons test (**G**, **H**, **I** and **K**), with *P*-values denoted as follows: *P < 0.05, **P < 0.01. Exact *P* values are available in the source data. Schematic in (**F**): Created in BioRender. Qin, X. (2026) https://BioRender.com/u2xdpiu.

Functional enrichment analysis showed that predicted targets of differentially expressed miRNAs were significantly associated with ion transport, protein oligomerization, cell adhesion, and cell migration (Fig. S7A, B; Fig. 4A). Notably, the enrichment of adhesion-, migration-, and cytoskeleton-related terms suggested a potential role for these sEV-associated miRNAs in regulating macrophage motility. Because these pathways matched the macrophage migration defect observed after PEDV-sEV treatment, we therefore focused on differentially expressed miRNAs to identify candidate cargos linked to impaired macrophage motility. To test this migration-linked miRNA program, we synthesized mimics for 21 candidate miRNAs and screened their effects on macrophage migration (Fig. S8A, B). Transwell assays identified miR-190a as the candidate with the most pronounced inhibitory effect on macrophage migration in this screen, reducing migration by 63.5% compared with controls (Fig. 4B–D, Fig. S8C). Real-time tracking confirmed this effect: miR-190a treatment reduced maximal radial displacement from 80 μm to 20 μm, with trajectories clustering near the origin, consistent with localized wandering and loss of directionality (Fig. 4E; Movie S1, S2). To determine whether sEV-associated miR-190a contributes to PEDV-sEV-induced migration suppression, we manipulated miR-190a expression in intestinal epithelial cells before sEV isolation. Overexpression increased miR-190a levels in both donor-cell-derived sEVs and recipient macrophages and enhanced the inhibitory effect of these sEVs on macrophage migration. Conversely, miR-190a knockdown reduced its sEV-associated abundance and partially attenuated the migration-suppressive activity of PEDV-induced sEVs (Fig. 4G–K). These results indicate that miR-190a is a functional contributor to PEDV-sEV-induced inhibition of macrophage migration.

### sEV-associated miR-190a inhibits macrophage migration by disrupting TMOD3-dependent F-actin remodeling

**Fig. 5:**
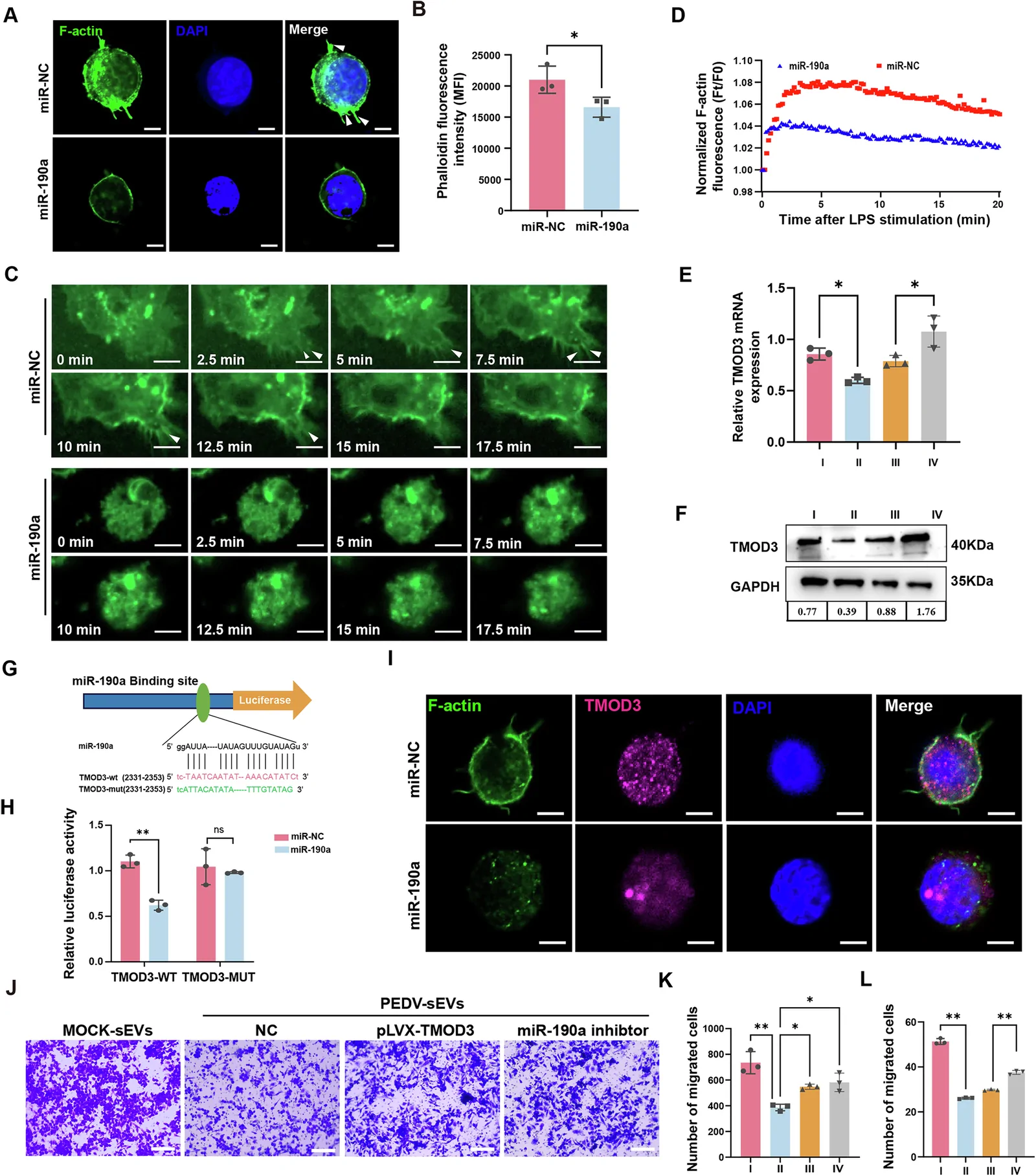
sEV-associated miR-190a suppresses macrophage migration through TMOD3-dependent F-actin remodeling. **A** Confocal microscopy showing reduced lamellipodia formation and F-actin enrichment in macrophages transfected with miR-190a mimic compared with control mimic. The images shown were representative of three independent replicates. Scale bar, 10 μm. **B** Flow-cytometric quantification of F-actin-associated phalloidin fluorescence in macrophages treated with miR-190a or control mimic, shown as mean fluorescence intensity (MFI) (*n* = 3 biologically independent samples). **C** Representative time-lapse confocal images of F-actin remodeling in LPS-stimulated macrophages transfected with miR-190a mimic or control mimic. Images were acquired every 10 s for 20 min; representative frames are shown at 2.5-min intervals. White arrowheads indicate ventral ROIs used for fluorescence analysis. Scale bar, 5 μm. **D** Representative F-actin fluorescence traces from the ventral ROIs indicated in (**C**), with one representative cell shown per condition. Background-corrected fluorescence was normalized to the value at 0 min and expressed as *F*_*t*_*/F*_*0*_. (E) RT–qPCR analysis of TMOD3 mRNA expression in macrophages transfected with miR-190a mimic or inhibitor (*n* = 3 biologically independent samples). **F** Western blot analysis of TMOD3 protein levels, with GAPDH as loading control. Group definitions: I, miR-NC; II, miR-190a mimic; III, miR-NC inhibitor; IV, miR-190a inhibitor. The images shown were representative of three independent replicates. **G** Schematic of wild-type (WT) and mutant (MUT) TMOD3 3′UTR luciferase constructs containing the predicted miR-190a binding site. **H** Dual-luciferase reporter assay validating the direct binding of miR-190a to the TMOD3 3′UTR. **I** Immunofluorescence showing co-localization of TMOD3 (red) and F-actin (green) in macrophages; nuclei are counterstained with DAPI (blue). Scale bar, 10 μm. The images shown were representative of three independent replicates. **J** Representative Transwell images showing macrophage migration after TMOD3 overexpression or miR-190a inhibition. Scale bar, 50 μm. **K** Quantification of migrated macrophages shown in (**J**, *n* = 3 biologically independent samples). **L** Flow-cytometric validation of migrated macrophages after TMOD3 overexpression or miR-190a inhibition under PEDV-sEV treatment. For each Transwell insert, five randomly selected, non-overlapping fields were imaged and averaged to generate one value per biological replicate (*n* = 3 biologically independent samples). Group definitions: I, MOCK-sEVs; II, PEDV-sEVs (NC); III, PEDV-sEVs with TMOD3 overexpression (pLVX-TMOD3); IV, PEDV-sEVs with miR-190a inhibition (miR-190a inhibitor). Data are presented as mean ± SD from *n* = 3 independent experiments. Each dot represents one independent experiment. Statistical significance was determined using two-tailed unpaired *t*-tests (**B** and **H**) and one-way ANOVA with Tukey’s multiple comparisons test (**E**, **K** and **L**), with *P*-values denoted as follows: *P < 0.05, **P < 0.01. Exact *P* values are available in the source data.

Given the inhibitory effect of miR-190a on macrophage motility, we next examined whether miR-190a altered F-actin organization, since dynamic F-actin assembly and disassembly are essential for macrophage polarization, protrusive activity, and directional migration^21^. Confocal microscopy revealed that miR-190a treatment reduced F-actin enrichment at the cell periphery and suppressed lamellipodia formation (Fig. 5A). Consistent with this imaging phenotype, flow-cytometric quantification of phalloidin-stained macrophages also showed a decrease in F-actin-associated fluorescence after miR-190a treatment (Fig. 5B). Representative live-cell imaging showed that, following LPS stimulation, control macrophages exhibited an increase in ventral F-actin fluorescence together with dynamic lamellipodial remodeling, whereas miR-190a-treated cells showed a weaker fluorescence response and less pronounced lamellipodial dynamics (Fig. 5C, D). These observations support that miR-190a attenuates activation-associated F-actin remodeling. To identify molecular targets mediating this effect, we used miRanda and PITA algorithms to predict potential binding genes of miR-190a, yielding 96 candidates (Fig. S8D), of which 11 were associated with actin remodeling and cell migration. Transfection of macrophages with miR-190a mimics led to downregulation of most candidate genes, with the strongest suppression observed for TMOD3 and FCAR (Fig. S8E).

TMOD3, a pointed-end actin-capping protein involved in actin–tropomyosin filament stability and cytoskeletal remodeling^22^, was selected for validation. Overexpression of miR-190a reduced TMOD3 mRNA and protein levels, whereas inhibition of miR-190a increased its expression (Fig. 5E, F). A conserved miR-190a binding site was identified in the TMOD3 3′-UTR (nt 2331–2353) (Fig. 5G), and dual-luciferase assays confirmed direct targeting: miR-190a significantly suppressed TMOD3-WT activity (*P* < 0.01), while the effect was lost with TMOD3-MUT (Fig. 5H). Confocal microscopy showed organized co-localization of TMOD3 with F-actin fibers in control macrophages. By contrast, miR-190a reduced overall TMOD3 abundance and disrupted its F-actin-associated distribution, leaving a disorganized punctate residual TMOD3 staining pattern (Fig. 5I). Finally, rescue experiments demonstrated that PEDV-induced sEVs inhibited macrophage migration, whereas miR-190a blockade or TMOD3 overexpression partially restored migratory activity (Fig. 5J-L). Collectively, these results suggest that sEV-associated miR-190a contributes to PEDV-sEV–induced macrophage migration defects by directly targeting TMOD3 and impairing F-actin remodeling.

### sEV-associated lncRNA TS2083 contributes to suppression of macrophage phagocytosis

**Fig. 6:**
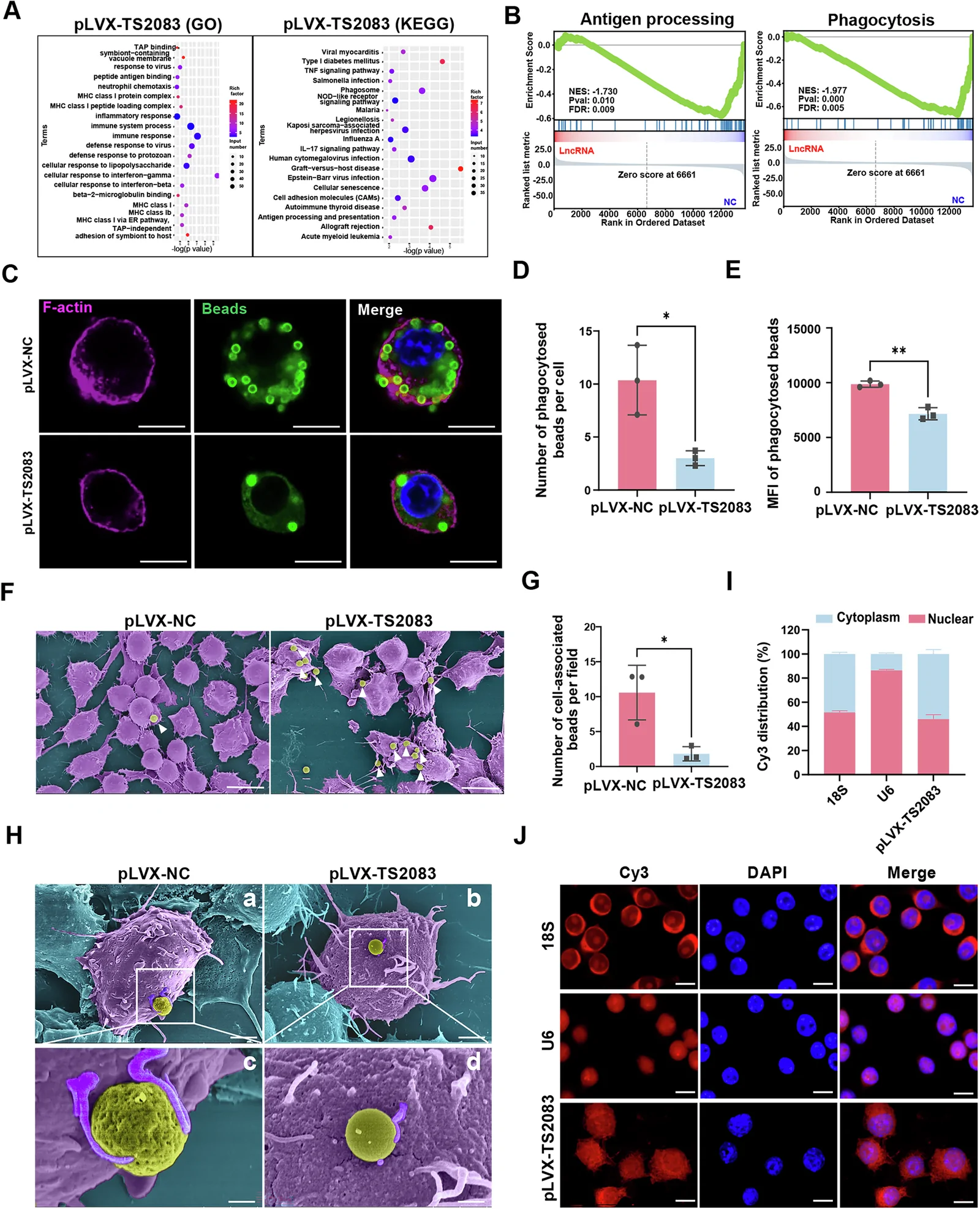
PEDV-induced epithelial sEVs suppress macrophage phagocytosis via lncRNA TS2083. Transcriptomic profiling of macrophages overexpressing lncRNA TS2083. **A** GO and KEGG enrichment analysis of downregulated genes. **B** GSEA enrichment plots showing suppression of antigen processing and phagocytosis pathways. Confocal microscopy analysis of bead phagocytosis. **C** Representative images of macrophages incubated with fluorescent beads. F-actin (red), beads (green), nuclei (blue, DAPI). Scale bar, 10 μm. Arrows indicate phagocytosed beads. **D** Quantification of bead uptake per cell shown in (**C**, *n* = 3 biologically independent samples). **E** Flow-cytometric quantification of bead uptake in macrophages overexpressing lncRNA TS2083, shown as mean fluorescence intensity (MFI) (*n* = 3 biologically independent samples). Scanning electron microscopy (SEM) showing bead adhesion and surface morphology. **F** Representative SEM images showing early bead adhesion to macrophages. Scale bar, 0.5 μm. White arrows indicate captured beads. **G** Quantification of cell-associated beads shown in (**F**, *n* = 3 biologically independent samples). **H** SEM images showing cell surface morphology and lamellipodia formation after lncRNA TS2083 treatment. Beads are pseudocolored yellow, and cup-shaped membrane structures are highlighted in purple. Scale bars: 20 μm (a, b), 1 μm (c, d). The images shown were representative of three independent replicates. Subcellular localization of lncRNA TS2083 in macrophages by RNA in situ hybridization. **I** Quantification of Cy3-labeled RNA distribution between nucleus and cytoplasm (*n* = 3 biologically independent samples). **J** Representative FISH images. LncRNA TS2083 (red, Cy3); nuclei (blue, DAPI). U6 and 18S served as positive controls. Scale bar, 5 μm. Data are presented as mean ± SD from *n* = 3 independent experiments. Each dot represents one independent experiment. Statistical significance was determined using two-tailed unpaired *t*-tests (**D**, **E** and **G**) and one-way ANOVA with Tukey’s multiple comparisons test (**I**), with *P*-values denoted as follows: *P < 0.05, **P < 0.01. Exact *P* values are available in the source data.

In addition to impairing macrophage migration, PEDV-induced sEVs also suppressed macrophage phagocytic activity. Because sEV-associated miR-190a contributed primarily to migration inhibition but did not fully account for the pronounced phagocytosis defect induced by PEDV-sEVs (Fig. S9A, B), we next examined sEV-associated lncRNAs as an additional host RNA cargo class. Differential profiling of sEV-associated lncRNAs revealed several candidates linked to immune regulation, ubiquitination, and phagocytosis-related pathways (Fig. S9C). Among them, TCONS_00002083, hereafter referred to as lncRNA TS2083, was highly enriched in PEDV-sEVs. In recipient macrophages, lncRNA TS2083 altered transcriptional programs related to antigen processing and presentation, immune signaling, phagosome function, and receptor-mediated endocytosis (Fig. 6A). Gene set enrichment analysis (GSEA) further showed downregulation of antigen-presentation and Fc receptor-mediated phagocytosis modules (Fig. 6B), prioritizing lncRNA TS2083 for functional validation of the macrophage phagocytosis defect.

Functional assays supported this role. Overexpression of lncRNA TS2083 significantly reduced macrophage bead uptake, as shown by a decrease in bead number per cell in microscopy-based analysis and reduced bead-associated fluorescence intensity by flow cytometry (Fig. 6C–E). SEM revealed fewer surface-associated beads during the early phase of phagocytosis in lncRNA TS2083-treated cells (Fig. 6F, G). High-magnification imaging further demonstrated defective F-actin-dependent phagocytic-cup formation, with minimal lamellipodial extension and engulfment, thereby providing a morphological basis for the reduced bead uptake (Fig. 6H).

To explore its mechanism, we assessed coding potential using the CNCI tool; all ORFs scored below the threshold, confirming its noncoding nature. Subcellular localization analysis by fluorescence in situ hybridization (FISH) showed lncRNA TS2083 distributed in both nucleus and cytoplasm (Fig. 6I–J). Given its cytoplasmic presence, the observed defects in phagocytic cup formation, and the central role of F-actin remodeling, we infer that cytoplasmic lncRNA TS2083 interferes with F-actin dynamics, thereby disrupting phagocytosis-related structures and suppressing macrophage phagocytic function.

### LncRNA TS2083 disrupts F-actin-dependent phagocytic cup formation via ubiquitin-mediated degradation of ANXA2

**Fig. 7:**
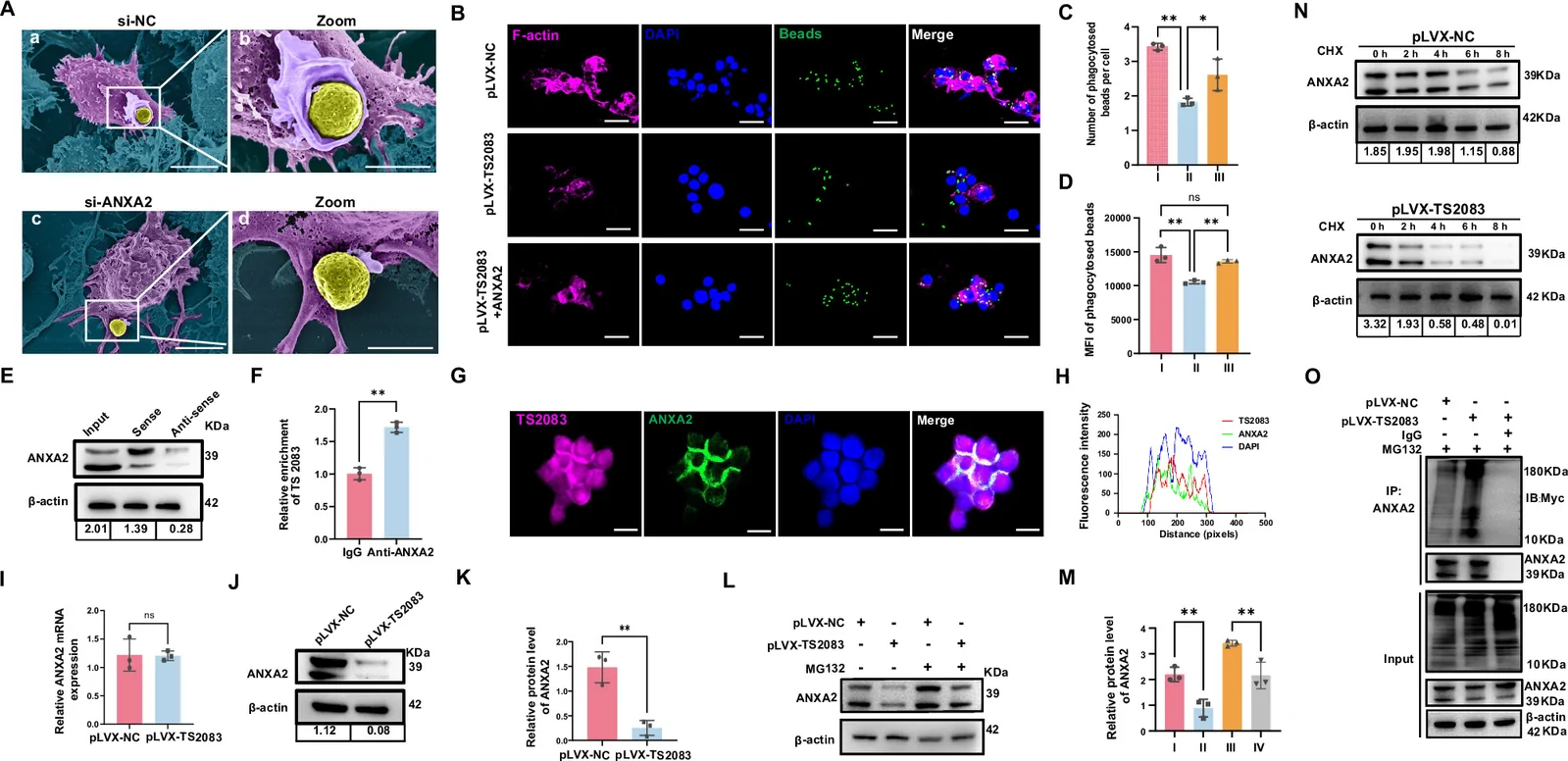
sEV-associated lncRNA TS2083 induces ANXA2 ubiquitination to block phagocytic cup formation and suppress macrophage phagocytosis. **A** Scanning electron microscopy showing bead phagocytosis in macrophages after ANXA2 knockdown. Beads are shown in yellow; phagocytic cups are indicated in purple. Scale bars: 5 μm (a, c), 2 μm (b, d). **B** Confocal microscopy showing bead phagocytosis in macrophages co-expressing lncRNA TS2083 and ANXA2. F-actin (red), beads (green), nuclei (blue, DAPI). Scale bar, 20 μm. **C** Quantification of bead uptake per cell shown in (B, *n* = 3 biologically independent samples). **D** Flow-cytometric quantification of bead-associated fluorescence in macrophages under the conditions shown in (**B**), shown as mean fluorescence intensity (MFI) (*n* = 3 biologically independent samples). Group definitions: I, pLVX-NC; II, pLVX-TS2083; III, pLVX-TS2083 + ANXA2. **E** RNA pull-down assay showing binding of lncRNA TS2083 to ANXA2, with antisense RNA as control. **F** RIP assay showing enrichment of lncRNA TS2083 by ANXA2 protein immunoprecipitation, quantified by RT–qPCR. **P < 0.01, *n* = 3 biologically independent samples. **G** Immunofluorescence and RNA FISH showing co-localization of ANXA2 (green) and lncRNA TS2083 (magenta, Cy3) in macrophages; nuclei (blue, DAPI). Scale bar, 10 μm. **H** Line-scan analysis of fluorescence intensities showing overlap between lncRNA TS2083 (red) and ANXA2 (green). RT–qPCR (**I**, *n* = 3 biologically independent samples) and Western blot (**J**) analysis of ANXA2 expression in macrophages overexpressing lncRNA TS2083. **K** Quantification of ANXA2 protein levels shown in (**J**, *n* = 3 biologically independent samples). Western blot analysis (**L**) and quantification (**M**, *n* = 3 biologically independent samples) of ANXA2 protein levels in macrophages overexpressing lncRNA TS2083 with or without MG132 treatment. Groups I–IV correspond to pLVX-NC, pLVX-TS2083, pLVX-NC + MG132, and pLVX-TS2083 + MG132, respectively. **N** Cycloheximide (CHX) chase assay showing ANXA2 protein stability in control and lncRNA TS2083-overexpressing macrophages. **O** Immunoprecipitation analysis of ANXA2 ubiquitination in lncRNA TS2083-overexpressing macrophages after MG132 treatment. ANXA2 was immunoprecipitated, and ubiquitination was detected by immunoblotting for Myc-tagged ubiquitin. Data are presented as mean ± SD from *n* = 3 independent experiments. Images shown in (**A**, **E**, **G**, and **N**–**O**) are representative of three independent biological replicates. Statistical significance was determined using two-tailed unpaired *t*-tests (**F**, **I** and **K**) and one-way ANOVA with Tukey’s multiple comparisons test (**C**, **D** and **M**),  with *P*-values denoted as follows: *P < 0.05, **P < 0.01. Exact *P* values are available in the source data.

To elucidate how lncRNA TS2083 regulates cytoskeletal remodeling and phagocytosis, we performed RNA pull-down coupled with mass spectrometry using RAW 264.7 cell lysates, identifying 58 candidate interacting proteins, including six F-actin regulators (Fig. S9D, E). Functional screening revealed that siRNA-mediated knockdown of ANXA2 significantly impaired macrophage uptake of fluorescent beads (Fig. S9F, G). ANXA2, a canonical membrane–cytoskeleton linker protein, regulates F-actin assembly and remodeling^23^. SEM confirmed that ANXA2 deficiency blocked nascent phagocytic cup formation (Fig. 7A), whereas ANXA2 overexpression in lncRNA TS2083–treated macrophages partially restored phagocytic capacity (Fig. 7B-D), establishing ANXA2 as a functional target.

We next assessed the lncRNA TS2083–ANXA2 interaction. RNA pull-down and RNA immunoprecipitation showed specific enrichment of lncRNA TS2083 in ANXA2 complexes (Fig. 7E, F). Combined FISH–immunofluorescence imaging demonstrated co-localization of lncRNA TS2083 and ANXA2 in the perimembranous cytoplasm (Fig. 7G, H), consistent with functional interplay at cytoskeletal regulatory sites.

Mechanistically, lncRNA TS2083 overexpression did not alter ANXA2 mRNA but reduced protein abundance (Fig. 7I-K). Cycloheximide chase assays revealed accelerated ANXA2 degradation, which was blocked by proteasome inhibition with MG132 (Fig. 7L–N). Moreover, immunoprecipitation confirmed increased ubiquitination of ANXA2 upon lncRNA TS2083 overexpression (Fig. 7O). Together, these findings support a mechanism whereby lncRNA TS2083 can promote ubiquitin-mediated degradation of ANXA2, thereby destabilizing ANXA2-dependent F-actin remodeling and contributing to impaired macrophage phagocytosis.

### PEDV-induced sEVs contribute to local and distal macrophage dysfunction

**Fig. 8:**
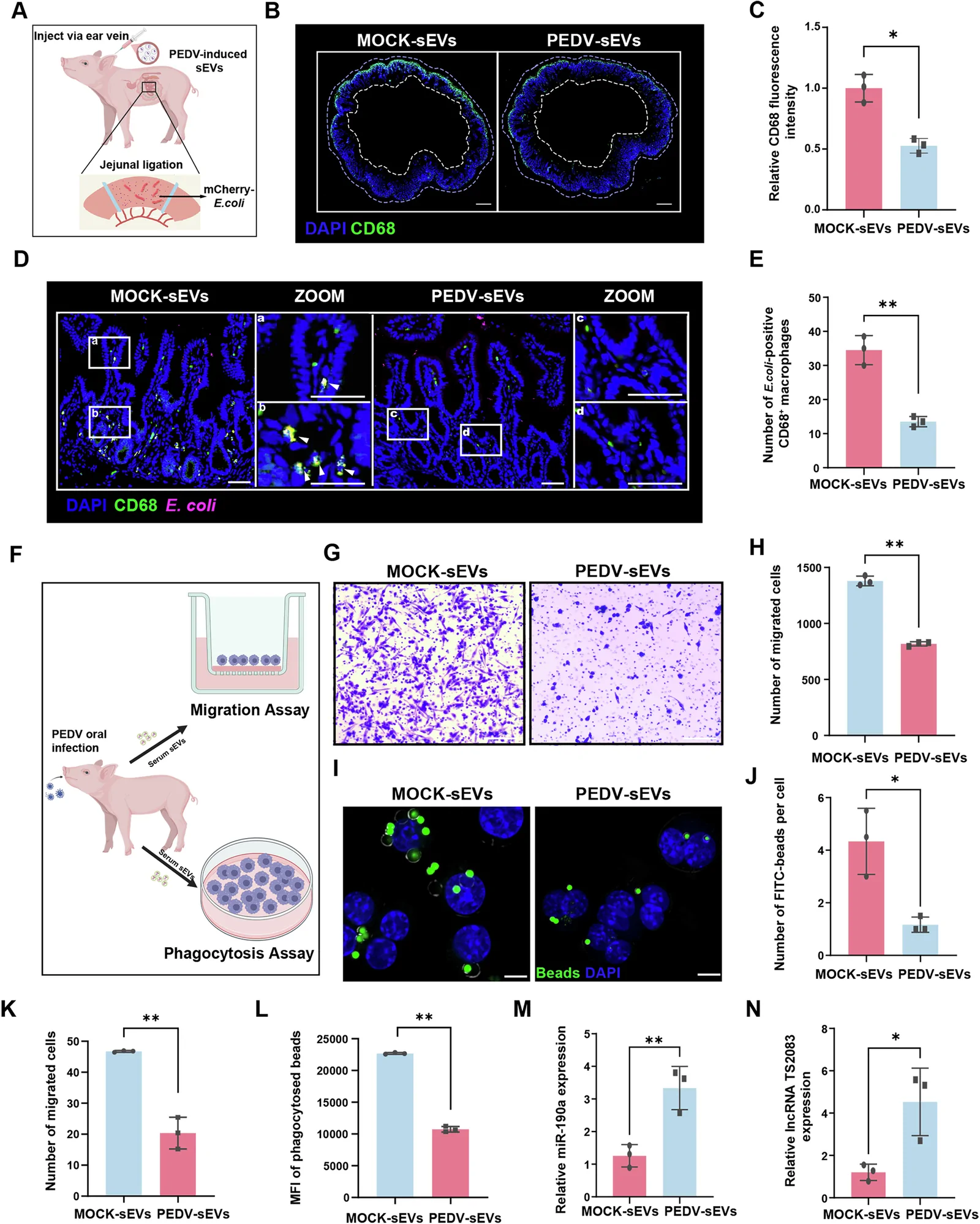
PEDV-induced epithelial sEVs contribute to local and distal macrophage dysfunction in piglets. In vivo evaluation of PEDV-induced epithelial sEVs in intestinal macrophage responses. **A** Schematic of intravenous sEV administration via the ear vein, followed 24 h later by jejunal ligated-loop injection of mCherry-labeled *E. coli*. **B** Representative in situ hybridization (FISH) images showing CD68⁺ macrophages in jejunal tissues. CD68⁺ macrophages are shown in green, and nuclei were counterstained with DAPI. Scale bar, 500 μm. **C** Quantification of relative CD68^+^ fluorescence intensity in jejunal tissues, *n* = 3 biologically independent samples. **D** Representative fluorescence images showing co-localization of CD68⁺ macrophages with mCherry-labeled *E. coli* in jejunal tissues. CD68⁺ macrophages are shown in green, *E. coli* in red, and nuclei in blue. White arrows indicate representative co-localization events. Scale bar, 50 μm. **E** Quantification of *E. coli* co-localization with CD68⁺ macrophages, *n* = 3 biologically independent samples. Functional analysis of serum-derived sEVs from PEDV-infected piglets. **F** Schematic of serum-sEV isolation and macrophage functional assays. Piglets were orally infected with PEDV or given mock treatment, and serum-derived sEVs were isolated by ultracentrifugation followed by anti-CD63 immunomagnetic enrichment at 24 h post-infection. **G** Representative Transwell images of alveolar macrophage migration after treatment with serum-derived sEVs. Scale bar, 50 μm. **H** Quantification of migrated alveolar macrophages in (**G**, *n* = 3 biologically independent samples). **I** Representative confocal images showing macrophage association with fluorescent beads. Nuclei were counterstained with DAPI. White arrows indicate bead-positive macrophages. Scale bar, 10 μm. **J** Quantification of bead uptake in (**I**, *n* = 3 biologically independent samples). **K**, **L** Flow-cytometric validation of alveolar macrophage migration and bead uptake after treatment with serum-derived sEVs, *n* = 3 biologically independent samples. **M**, **N** RT–qPCR analysis of miR-190a and lncRNA TS2083 levels in serum-derived sEVs, *n* = 3 biologically independent samples. Data are presented as mean ± SD; *n* = 3 piglets per group. Each dot represents one individual piglet. Statistical comparisons between two groups were performed using two-tailed unpaired *t*-test. *P < 0.05, **P < 0.01. Exact *P* values are available in the source data. Schematics in (**A**) and (**F**): Created in BioRender. Qin, X. (2026) https://BioRender.com/u2xdpiu.

To validate the immunosuppressive role of PEDV-induced sEVs in vivo, we intravenously injected sEVs from PEDV-infected intestinal epithelial cells into 10-day-old piglets and assessed their effects using ligated intestinal-loop assays (Fig. 8A). Fluorescence in situ hybridization combined with confocal microscopy showed that PEDV-sEV treatment reduced intestinal CD68 signal and decreased the association of mCherry-labeled *E. coli* with CD68-positive macrophages compared with the control treatment (Fig. 8B–E). Because intestinal epithelial sEVs can cross the mucosal barrier and enter the circulation via blood or lymph^24,25^, we next evaluated their potential distal effects. Serum sEVs isolated at 24 h after oral PEDV infection significantly impaired alveolar macrophage migration and bead uptake. Microscopy-based assays showed fewer migrated macrophages and reduced bead uptake per cell, and these effects were independently confirmed by flow cytometry (Fig. 8F–L). RT–qPCR confirmed enrichment of miR-190a and lncRNA TS2083 in these circulating sEV preparations compared with controls (Fig. 8M, N). Together, these findings indicate that PEDV-induced epithelial sEVs impair intestinal macrophage function and that circulating sEVs from infected piglets suppress alveolar macrophage activity ex vivo. These results support a role for sEV-associated signals in macrophage dysfunction during PEDV infection.

## Discussion

Immune surveillance at mucosal surfaces is essential for limiting viral infection, but effective mucosal defense depends on coordinated communication between epithelial cells and immune effector cells. During intestinal infection, epithelial-derived cytokines and chemokines can recruit monocytes and macrophages into the mucosa; however, successful viral control requires these recruited cells to retain key effector functions. Under physiological conditions, macrophages contribute to mucosal defense by clearing pathogens, presenting antigens, and supporting epithelial immune responses. Here, we identify an epithelial sEV-associated mechanism that contributes to macrophage dysfunction during PEDV infection, providing mechanistic insight into PEDV mucosal pathogenesis. Although PEDV infection activates epithelial PRR, interferon, cytokine, and chemokine programs, macrophage effector programs related to migration, phagocytosis, lysosomal activity, and antigen presentation show reduced transcriptional activity. Our co-culture and secretome-fractionation experiments further suggest that this dysfunction occurs within an inflammatory epithelial secretome but is not explained solely by major soluble inflammatory cytokines. Instead, sEVs released from PEDV-infected intestinal epithelial cells are internalized by macrophages and impair macrophage migration and phagocytosis in vitro; in vivo, PEDV-induced epithelial sEVs reduce mucosal CD68⁺ macrophage accumulation and bacterial uptake in a piglet sEV-transfer model. These findings extend the conventional view of PEDV immune evasion from direct interference with interferon or cytokine signaling to sEV-associated modulation of epithelial–macrophage communication. Together, these findings suggest that PEDV-induced remodeling of epithelial sEV cargo contributes to macrophage dysfunction during infection.

sEVs are nanosized (30–150 nm) bilayered vesicles enriched with proteins, nucleic acids, and lipids that mediate intercellular communication and regulate diverse biological processes. Extensive studies have established that tumor-derived sEVs play pivotal roles in shaping the immune landscape of the tumor microenvironment, where they modulate immune cell activation, antigen presentation, and cytokine signaling to facilitate tumor immune evasion^26,27^. In recent years, accumulating evidence has revealed that viruses similarly exploit host-derived sEVs to establish immunosuppressive microenvironments that promote viral persistence and immune escape. For example, EV71-derived sEVs package replication-competent viral RNA and miR-146a to enable receptor-independent transmission and suppress type I interferon^28^; EBV-derived sEVs carrying LMP1 and galectin 9 remodel antigen-presenting cell and T cell responses^29^; and CVB3 uses sEVs to deliver infectious particles into receptor-negative immune cells, thereby escaping neutralizing antibodies and receptor blockade^30^. Compared with soluble factors or single signaling pathways, sEV lipid bilayers protect cargo from degradation, while membrane proteins facilitate uptake and host-derived components may reduce immune recognition^31,32^. Leveraging these features, PEDV-induced sEVs may deliver immunoregulatory molecules to macrophages and sustain vesicle-associated signaling within the intestinal mucosal environment. These properties may allow sEV-associated signals to persist locally and may be relevant to PEDV mucosal pathogenesis, viral persistence, and disease control. However, their direct effects on viral shedding, disease severity, secondary infection susceptibility, and vaccine efficacy remain to be tested in future in vivo studies.

Within PEDV-induced sEVs, we identified miR-190a as a functionally validated cargo that contributes to impaired macrophage migration—an essential effector function of innate immunity. Since its identification, miR-190a has been studied primarily in the context of tumor biology, where it has been implicated in cancer-related processes including cell proliferation and metastasis. In contrast, its role in immune cells remains largely unexplored^33,34^. Our findings link sEV-delivered miR-190a to TMOD3-dependent actin remodeling and macrophage migration. TMOD3 is an actin-regulatory pointed-end capping protein that controls actin filament length, stability, and turnover^35,36^. Although pointed-end capping can restrict filament elongation, proper TMOD3 function is also required to preserve actin-network integrity; loss of TMOD3 may disturb filament-length control, resulting in abnormal actin elongation, filament breakage, and impaired lamellipodial dynamics^37^. Because macrophage migration requires coordinated F-actin remodeling to support polarization, protrusive activity, and directional movement^38,39^, repression of TMOD3 by sEV-delivered miR-190a provides a mechanistic explanation for the impaired F-actin remodeling and migratory dysfunction observed in PEDV-sEV-treated macrophages. Together, these findings support the miR-190a/TMOD3 axis as a mechanistically defined contributor to PEDV-sEV-induced impairment of macrophage migration.

In addition to impairing migration, PEDV-induced sEVs also suppress macrophage phagocytosis. This process requires receptor–ligand engagement to trigger F-actin polymerization and membrane remodeling, driving pseudopodia extension and phagocytic cup formation. We found that sEVs from PEDV-infected cells inhibited cup assembly and reduced particle uptake. Mechanistic analyses identified a long noncoding RNA, lncRNA TS2083, a candidate contributor to this phagocytic defect. Absent from major databases (NONCODE, GENCODE and Ensembl), lncRNA TS2083 appears to represent a previously unannotated transcript. Functional studies showed that lncRNA TS2083 interacts with ANXA2 in the cytoplasm and promotes its ubiquitin-mediated degradation, thereby impairing phagocytosis. ANXA2 is a Ca²⁺-dependent membrane-binding protein that links phospholipids to F-actin and is critical for pseudopodia extension and phagocytic cup stability by coordinating actin dynamics and small GTPase localization^23^. Thus, lncRNA TS2083-induced ANXA2 degradation provides a mechanistically supported explanation for part of the phagocytic defect induced by PEDV sEVs. Although studies on lncRNA-mediated ubiquitination remain limited, accumulating evidence indicates that lncRNAs can modulate protein stability by serving as scaffolds for E3 ligases, altering protein conformation to expose lysine residues, or interfering with deubiquitinases^40,41^. LncRNA TS2083 may exploit one of these mechanisms to promote ANXA2 degradation, a hypothesis warranting further investigation.

Together with the miR-190a/TMOD3 pathway described above, these findings define two mechanistically supported sEV-associated ncRNA–cytoskeleton axes that affect distinct macrophage effector programs: migration and phagocytosis. The downstream cytoskeletal effectors identified in these axes, TMOD3 and ANXA2, showed high protein-sequence conservation among pig, human, and mouse (Supplementary Table S3), supporting the possibility that these cytoskeleton-related effector pathways may have broader cross-species relevance. Consistently, in a preliminary THP-1-derived human macrophage-like cell model, PEDV-sEV treatment induced a similar direction of functional change in migration and phagocytosis. These observations suggest that macrophage responses to sEV-associated immunoregulatory cues may not be restricted to porcine cells. However, whether the specific PEDV-sEV-associated ncRNA cargos exert equivalent functions across species remains to be determined. Thus, these axes provide a mechanistic framework for PEDV-sEV-mediated macrophage dysfunction, while future cargo-specific and in vivo studies will be required to define the relative contribution and cross-species relevance of individual vesicular components during PEDV infection.

The in vivo sEV-transfer experiment further extends these cell-based observations to the intestinal mucosal environment. In this model, intravenous administration of PEDV-induced epithelial sEVs followed by jejunal ligated-loop exposure to mCherry-labeled *E. coli* reduced CD68⁺ macrophage accumulation in villi and decreased bacterial internalization by individual macrophages. These findings indicate that PEDV-induced epithelial sEVs can transfer a macrophage-suppressive phenotype in vivo, affecting both mucosal macrophage recruitment and local phagocytic capacity under experimental conditions. Building on our demonstration that PEDV-induced sEVs suppress intestinal macrophage functions in vivo, we further detected serum sEVs enriched in miR-190a and lncRNA TS2083 at 24 h after oral infection. These sEVs also impaired the migratory and phagocytic capacity of alveolar macrophages. With their protective lipid bilayer, sEVs resist enzymatic degradation and remain stable over long distances, thereby serving as potential mediators of distal immune regulation across mucosal sites. Similar mechanisms have been described in other viral infections: sEVs from JEV-infected cells carry viral antigens, evade neutralizing antibodies, and traverse the blood–brain and blood–placental barriers to enhance viral dissemination and pathogenicity in mice^42^; sEVs released from HBV-infected hepatocytes deliver IFITM2 to dendritic cells, suppressing endogenous IFN-α production and reducing the antiviral efficacy of exogenous IFN-α^43^. Collectively, these findings indicate that PEDV infection is associated with circulating sEVs that can transmit macrophage-suppressive signals to distal immune cells. Previous studies have shown that although the small intestine is the primary site of infection, PEDV can also damage noncanonical tissues such as the spleen and liver and increase the susceptibility of extraintestinal mucosal sites, including the respiratory tract, to secondary pathogens^44,45^. Our findings suggest that circulating sEVs associated with PEDV infection may contribute to local-to-distal macrophage modulation and distal immune effects. At the cargo level, miR-190a/TMOD3 and lncRNA TS2083/ANXA2 represent mechanistically defined ncRNA–cytoskeleton axes.

**Fig. 9:**
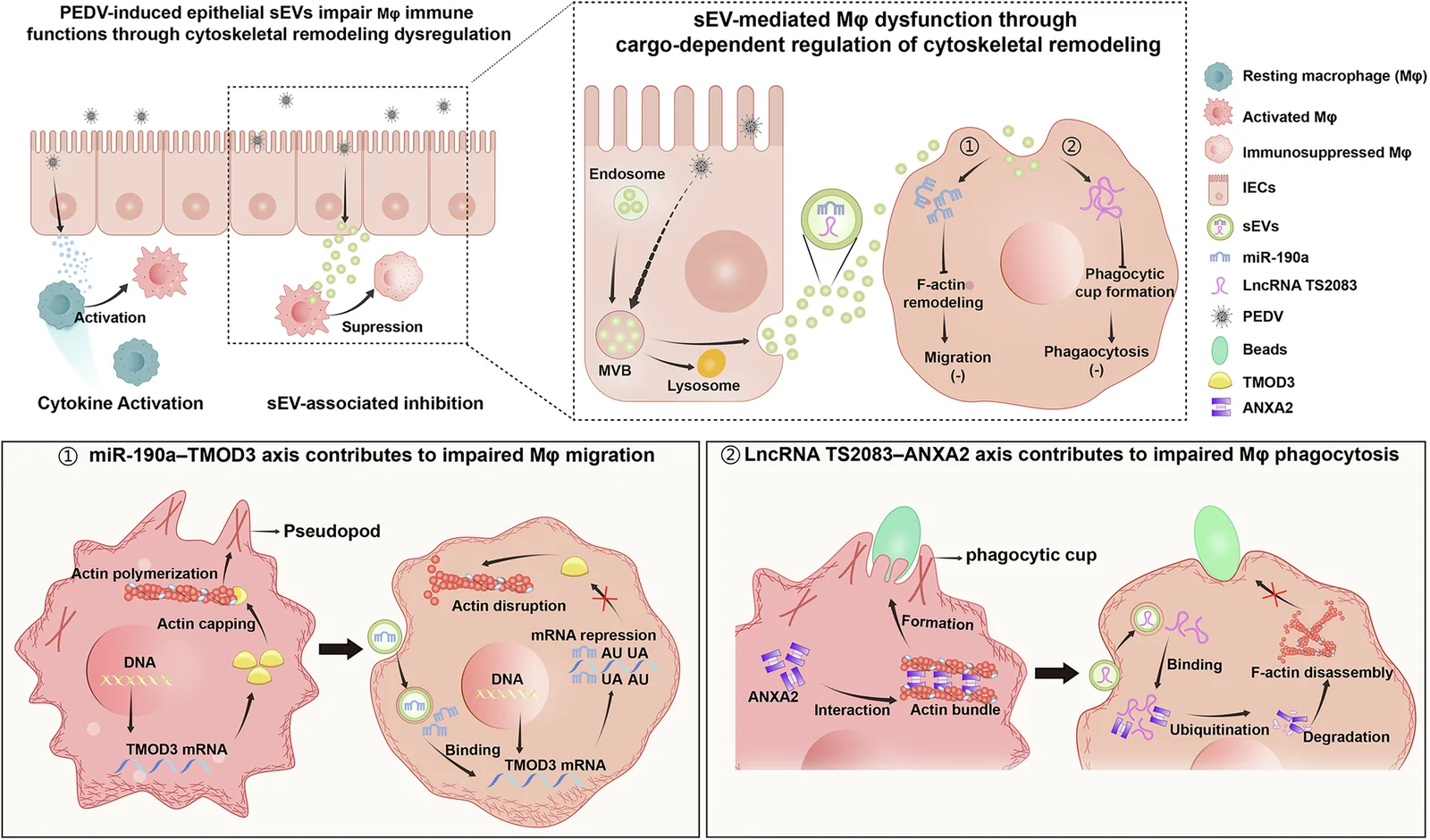
Schematic model of PEDV-induced epithelial sEVs contributing to macrophage dysfunction during intestinal infection. PEDV infection promotes the release of epithelial sEVs enriched in host non-coding RNAs. Among these cargos, miR-190a contributes to impaired macrophage migration by targeting the actin-regulatory protein TMOD3 and disrupting F-actin remodeling, whereas lncRNA TS2083 contributes to defective phagocytosis by promoting ubiquitin-mediated degradation of ANXA2 and impairing phagocytic cup formation. Together, these sEV-associated non-coding RNA pathways impair macrophage motility and phagocytic function during PEDV infection.

In conclusion, this study demonstrates that PEDV-induced intestinal epithelial sEVs contribute to local and distal macrophage dysfunction. These vesicles carry functional noncoding RNAs that act on distinct macrophage effector functions: miR-190a downregulates TMOD3, disrupting F-actin remodeling and thereby restricting migration, while lncRNA TS2083 promotes ubiquitin-mediated degradation of ANXA2, destabilizing phagocytic cups and suppressing phagocytosis (Fig. 9). Collectively, our findings expand the paradigm of PEDV immune evasion from the classical concept of interferon antagonism to sEV-mediated reprogramming of epithelial–macrophage communication and cytoskeletal effector programs, clarifying how PEDV affects mucosal macrophage function. Beyond defining a mechanism of PEDV-induced macrophage dysfunction, these findings provide a rationale for host-directed strategies aimed at restoring macrophage effector activity by targeting PEDV-induced sEV release or the miR-190a/TMOD3 and lncRNA TS2083/ANXA2 axes. Future in vivo studies will be needed to determine whether their modulation can reduce PEDV disease severity, viral shedding, or mortality.

## Methods

### Animals

All piglets used in this study were female three-way crossbred pigs. Three-day-old neonatal piglets used for the PEDV challenge experiments, two-week-old piglets used as donors for primary alveolar macrophage isolation, and ten-day-old piglets used for the intestinal ligated-loop and serum-sEV experiments were obtained from a commercial farm in Gaochun, Jiangsu Province, China. Antigen–antibody testing confirmed that all animals were negative for PEDV, transmissible gastroenteritis virus (TGEV), porcine reproductive and respiratory syndrome virus (PRRSV), porcine circovirus type 2 (PCV2), and porcine respiratory coronavirus (PRCV). To eliminate the influence of maternally derived antibodies, neonatal piglets were artificially reared throughout the experiments. After arrival at the animal facility, piglets were randomly assigned to experimental groups and housed by group in designated pens, with different treatment groups separated to prevent cross-contamination. Piglets were allowed to acclimatize for 24 h before experimentation to minimize stress associated with transport and environmental change.

All animal procedures were conducted in accordance with institutional guidelines and were approved by the Animal Experiment Ethics Committee of Nanjing Agricultural University (protocol no. NJAULLSC2023089). Sex was not included as an experimental variable, and no sex-based analysis was performed.

### Viruses, cell lines, and bacterial strains

The highly virulent porcine epidemic diarrhea virus (PEDV) strain CHYJ130330 used in this study was kindly provided by the Guangdong Haid Institute of Animal Husbandry and Veterinary Medicine and subsequently maintained in our laboratory. Its complete genome sequence has been published and is available in GenBank under accession number KJ020932.

The 3D4/21 porcine alveolar macrophage cell line (ATCC CRL-2843) and IPEC-DQ cells were maintained in our laboratory, whereas RAW 264.7 murine macrophage cells (ATCC TIB-71), HEK293T cells (ATCC CRL-3216), and THP-1 cells (ATCC TIB-202) were obtained from the American Type Culture Collection. IPEC-DQ is a PEDV-susceptible subclone derived from IPEC-J2 cells by limiting dilution and selected for enhanced susceptibility to PEDV infection, as previously described by Zhang et al.^46^. Before use, IPEC-DQ cells were validated for the expression of the intestinal epithelial markers EpCAM and villin and for greater PEDV susceptibility than the parental IPEC-J2 cells (Fig. S3B, E). The 3D4/21, IPEC-DQ, RAW 264.7, and HEK293T cells were cultured in DMEM, whereas THP-1 cells were cultured in RPMI 1640. Both media were supplemented with 10% fetal bovine serum, 100 U/mL penicillin, and 100 μg/mL streptomycin. All cells were maintained at 37 °C in a humidified atmosphere containing 5% CO₂. All cell lines were routinely confirmed to be free of mycoplasma contamination. Sex was not considered as an experimental variable in the cell-line experiments.

Primary alveolar macrophages were isolated from two-week-old female piglets. Briefly, piglets were euthanized by carotid exsanguination, and lungs were immediately excised under sterile conditions. Bronchoalveolar lavage fluid (BALF) was collected by gently infusing and withdrawing pre-chilled PBS through an endotracheal cannula while massaging the lung tissue. The BALF was centrifuged, and the supernatant was discarded to obtain a cell pellet, which was washed repeatedly with PBS by centrifugation. Cells were resuspended in RPMI-1640 medium and cultured at 37 °C. Non-adherent cells were removed by medium exchange, yielding highly purified alveolar macrophages.

The mCherry-expressing *Escherichia coli* (*E. coli*), kindly provided by Prof. Zhe Ma (College of Veterinary Medicine, Nanjing Agricultural University), was grown in LB medium at 37 °C, purified, and quantified by plate counting prior to use.

### Lentivirus, inhibitors and mimics, plasmid construction, and cell transfection

miRNA mimics, inhibitors, and siRNAs were purchased from Sangon Biotech (Shanghai, China). The lncRNA TS2083 probe was obtained from Ribobio (Guangdong, China), and U6 and 18S probes were purchased from GenePharma (Shanghai, China). Lentivirus carrying lncRNA TS2083 was provided by OBiO Technology (Shanghai, China). The laboratory-preserved pLVX-3flag-MCS-P2A vector was used to clone full-length porcine TMOD3 for stable overexpression, with the empty vector serving as a control. The UB-myc plasmid was obtained from GenePharma (Shanghai, China).

All oligonucleotides and plasmids were transfected into cells using Lipofectamine 3000 (Invitrogen, Carlsbad, CA, USA) following the manufacturer’s protocol. The sequences of miRNA mimics and inhibitors are provided in Supplementary Table 1.

### Reagents

The Dual Luciferase Reporter Gene Assay Kit (RG027) was purchased from Beyotime (Shanghai, China). RNA immunoprecipitation (RIP) RNA-Binding Protein Immunoprecipitation Kit (Bes5101) was obtained from BersinBio (Guangzhou, China). Magnetic RNA-Protein Pulldown Kit (20164), DMEM/F12 medium (12634010), and ActinGreen™ 488 ReadyProbes™ live-cell dye (R37110) were purchased from Thermo Fisher Scientific (Waltham, MA, USA). T7 High Yield RNA Transcription Kit (TR101) and BCA Protein Assay Kit (E112-01/02) were obtained from Vazyme (Nanjing, China). Fetal bovine serum (FBS, 10100147C) was purchased from Gibco (New York, NY, USA). Lipofectamine 3000 Transfection Kit (L3000015) and TRIzol Reagent (15596026CN) were purchased from Invitrogen (Carlsbad, CA, USA). Phosphate-buffered saline (PBS, SH30256.01) was purchased from Hyclone (Logan, UT, USA). HEPES (H0887), Collagenase II (V900892-100MG), EDTA (E8008), and DNase I (9003-98-9) were purchased from Sigma-Aldrich (St. Louis, MO, USA). Dispase II (4942078001) was obtained from Roche (Basel, Switzerland). LPS (L8880) was purchased from Solarbio (Beijing, China). Complement component C5a (C075) was purchased from Novoprotein (Suzhou, China). Fluorescently carboxylated microspheres (117687-5) were obtained from Polysciences (Warrington, PA, USA).

Anti-CD63 monoclonal antibody (ab134045; 1:200 dilution), anti-CD81 monoclonal antibody (ab109201; 1:200 dilution), anti-Calnexin monoclonal antibody (ab133615; 1:200 dilution) and anti-HSP70 polyclonal antibody (ab181606; 1:200 dilution) were purchased from Abcam (Cambridge, UK). Rabbit anti-TMOD3 (12521-1-AP; 1:200 dilution) and mouse anti-ANXA2 monoclonal antibody (66035-1-Ig; 1:200 dilution) were obtained from Proteintech (Wuhan, China). Horseradish peroxidase (HRP)-conjugated secondary antibodies (1:5000 dilution) were purchased from Cell Signaling Technology (Danvers, MA, USA). Fluorescently labeled secondary antibody (594-conjugated goat anti-rabbit IgG, 771384ES60; 1:200 dilution) (594-conjugated goat anti-mouse IgG, 33212ES60; 1:200 dilution) was purchased from YEASEN (Shanghai, China). The anti-PEDV N protein monoclonal antibody was custom-made by Jinsirui Biotechnology (Nanjing, China) and preserved in our laboratory (1:200 dilution).

### Virus challenge experiments

To investigate the impact of PEDV infection on the intestinal cellular transcriptome, an oral challenge experiment was performed. Three-day-old piglets of similar body weight were randomly assigned to a PEDV-infected group or a negative control group (*n* = 4 per group). Piglets in the PEDV group were inoculated via gastric tube with 1 mL of viral suspension at a titer of 1 × 10⁷ PFU/mL, whereas control piglets received an equal volume of PBS. At 36 h post-challenge, piglets in the PEDV group developed diarrhea and were euthanized. For scRNA-seq, jejunal mucosal samples from two independent piglets per group were processed separately for single-cell suspension preparation, library construction, and sequencing. Sample identity was retained throughout preprocessing and downstream analysis. The remaining intestinal segments were fixed in 4% paraformaldehyde, embedded in paraffin, and sectioned at 5 μm for histopathological examination and immunofluorescence staining.

### sEV isolation, characterization and internalization

IPEC-DQ cells were infected with PEDV at a multiplicity of infection (MOI) of 1 for 1 h at 37 °C, washed with PBS, and cultured in fresh DMEM/F12 at 37 °C in 5% CO₂ for an additional 24 h. Culture supernatants were sequentially centrifuged at 300 × *g* for 10 min and 2000 × *g* for 30 min, filtered through a 0.22-μm membrane, and ultracentrifuged at 110,000 × *g* for 70 min. The pellets were resuspended in PBS, washed by a second ultracentrifugation under the same conditions, and finally resuspended in PBS. Because the biogenesis of these vesicles was not directly determined, the isolated vesicle preparations are operationally referred to as small extracellular vesicles (sEVs) according to their isolation procedure, morphology, particle size distribution, and marker profile. To prepare sEV-depleted conditioned medium as a non-vesicular secretome control, the supernatant remaining after ultracentrifugation was carefully collected without disturbing the sEV pellet. The sEV-depleted conditioned medium was volume-normalized to the corresponding total conditioned medium and used for macrophage migration and phagocytosis assays. Matched donor-cell lysates were collected from the same IPEC-DQ cultures used for sEV isolation and analyzed by Western blotting for GAPDH to assess donor-cell input. For macrophage treatment experiments, sEV preparations from MOCK-, WIV PEDV-, and PEDV-treated epithelial cells were quantified using the BCA assay. Unless otherwise indicated, macrophages were treated with equal total protein amounts from sEV preparations rather than equal volumes of conditioned medium.

For transmission electron microscopy (TEM), freshly prepared sEVs were placed onto copper grids and negatively stained with 2% phosphotungstic acid for 2 min, and morphology was examined using a Hitachi H-7500 electron microscope. Particle size distribution and concentration of independent sEV preparations were determined by nanoparticle tracking analysis (NTA) using a ZetaView instrument (Particle Metrix, Germany) calibrated with standard microspheres, and data were processed with ZetaView 8.05.14 SP7 software. Total particle yield was calculated by normalizing particle concentration to the final resuspension volume. For Western blot characterization, sEV samples were lysed on ice with RIPA buffer, quantified by BCA, and analyzed for sEV-associated markers and negative markers.

For downstream macrophage treatment and functional assays, sEVs obtained by ultracentrifugation were further enriched using anti-CD63 immunomagnetic beads, and bead-bound sEV preparations were collected with a magnetic stand. Unless otherwise indicated, the term sEV preparations refers to vesicles isolated by ultracentrifugation followed by anti-CD63 immunomagnetic enrichment. For fluorescence labeling, sEVs were incubated with the DiI membrane dye (Beyotime, China) for 20 min, and excess dye was removed by centrifugation and washing. DiI-labeled sEVs were subsequently incubated with macrophages for the indicated durations, and internalization was visualized using a Nikon A1 confocal microscope (Nikon, Japan).

### Single-cell RNA-seq library construction and sequencing

Jejunal mucosal tissues from individual piglets were processed separately for single-cell RNA sequencing. Tissues were washed in ice-cold PBS and enzymatically dissociated using HEPES, EDTA (Sigma, E8008), Collagenase II (Sigma, V900892-100MG), and Dispase II (Roche, 4942078001). After erythrocyte removal, cell viability and cell counts were assessed by acridine orange/propidium iodide (AO/PI) staining, and debris was removed when necessary using Miltenyi reagents (Miltenyi, 130-109-398/130-090-101). Live cells were washed twice with RPMI-1640 and resuspended in RPMI-1640 supplemented with 2% fetal bovine serum (FBS) at a final concentration of 1 × 10⁶ cells/mL.

Single-cell RNA-seq libraries were prepared using the SeekOne® DD Single Cell 3′ Library Preparation Kit (SeekGene, catalog no. K00202). Briefly, an appropriate number of cells from each individual sample was mixed with reverse-transcription reagents and loaded into the sample wells of a SeekOne® chip S3. Barcoded hydrogel beads and partitioning oil were added into the corresponding wells of the chip, and emulsion droplets were generated. Reverse transcription was performed at 42 °C for 90 min, followed by inactivation at 85 °C for 5 min.

After droplet breaking, cDNA was purified and amplified by PCR. Amplified cDNA was cleaned, fragmented, end-repaired, A-tailed, and ligated to sequencing adaptors. Indexed PCR was then performed to enrich 3′ poly(A)-containing cDNA fragments incorporating both cell barcodes and unique molecular identifiers (UMIs). Indexed libraries were cleaned using VAHTS DNA Clean Beads (Vazyme, N411-01), quantified with a Qubit fluorometer (Thermo Fisher Scientific, Q33226), and quality-assessed using a Bio-Fragment Analyzer (Bioptic, Qsep400). Final libraries were sequenced on an Illumina NovaSeq X Plus platform with paired-end 150-bp read length.

### Processing the single-cell RNA sequencing data

Raw sequencing reads from the in-house scRNA-seq dataset were processed with fastp to trim primer and adapter sequences and remove low-quality bases. Clean reads were then processed using SeekSoul tools (SeekGene, v1.2.1) for cellular barcode demultiplexing, alignment to the pig reference genome (*Sus scrofa* 11.1, Ensembl release 103), and generation of feature–barcode matrices. The resulting matrices were imported into the Seurat R package (v4.1.1) for downstream analyses.

Low-quality cells were filtered according to the number of detected genes, total UMI counts, and the proportion of mitochondrial-gene transcripts. Cells with outlier gene numbers or UMI counts were removed using sample-specific distribution-based filtering, and cells with high mitochondrial-gene proportions were excluded using the median absolute deviation (MAD)-based criterion. After filtering, we normalized the data with NormalizeData, selected 2000 highly variable genes with FindVariableFeatures using the variance-stabilizing transformation (vst) method, and scaled the expression values with ScaleData.

Sample identity was retained throughout quality control, dimensionality reduction, clustering, cell-type annotation, differential expression analysis, and visualization. Harmony was used for sample integration and batch correction, primarily for dimensionality reduction, clustering, and visualization. Differential expression analysis and pseudobulk analysis were performed using non-Harmony-corrected expression matrices. Principal component analysis (PCA) was performed, shared nearest-neighbor graphs were constructed using FindNeighbors, and clusters were identified using FindClusters. Uniform manifold approximation and projection (UMAP) was used for visualization.

Cell types were manually annotated based on canonical marker genes and cross-checked with reported lineage-specific features of intestinal epithelial, stromal, and immune cell populations. Where necessary, major cell populations were further subclustered to improve cell-type resolution. Cell-type composition across conditions was summarized using the relative proportion of each annotated cell population and the ratio of observed to expected cells (ROE). ROE was calculated from a cell type × condition contingency table to visualize relative enrichment or depletion patterns among major cell populations. ROE values greater than 1 indicate relative enrichment, whereas ROE values less than 1 indicate relative depletion. These cell-composition and ROE summaries were used to describe condition-associated shifts in major intestinal cell populations.

### Cell–cell communication analysis

Cell–cell communication analysis was performed using CellChat (v2.1.0) with the CellChatDB v2 database. The analysis was based on normalized single-cell expression matrices, and cell groups containing fewer than 10 cells were excluded. Interaction strength was inferred using the truncatedMean method with the trim parameter set to 0.25, based on the mass-action model implemented in CellChat. We focused primarily on interactions between epithelial cells and myeloid populations, including macrophages, monocytes, and dendritic cells. Condition-associated interaction patterns were compared between groups and used to guide interpretation of epithelial–immune communication during PEDV infection.

### Functional enrichment analysis

Functional enrichment analysis was performed using clusterProfiler (v4.2.0). Gene Ontology (GO) and Kyoto Encyclopedia of Genes and Genomes (KEGG) over-representation analyses were conducted using enrichGO and enrichKEGG, respectively. Gene set enrichment analysis (GSEA) was performed using the GSEA function in clusterProfiler based on ranked gene lists. Gene sets were obtained from the Molecular Signatures Database (MSigDB, v7.0). GO, KEGG, and GSEA analyses were used for pathway-level interpretation of transcriptional programs across annotated cell populations. Where required, porcine genes were mapped to corresponding orthologous gene symbols before enrichment analysis. Protein sequences of TMOD3 and ANXA2 from pig, human, and mouse were retrieved from public protein databases. Pairwise sequence alignments were performed using Clustal Omega or BLASTP, and alignment coverage, percentage identity, and percentage similarity were summarized in Supplementary Table 3.

### Sample-aware pseudobulk analysis

To assess whether the major transcriptional patterns observed at the cell level were directionally retained at the sample level, we performed a sample-aware pseudobulk analysis. For each major cell type, raw count matrices from cells derived from the same independent piglet sample were aggregated by gene to generate sample-level pseudobulk count matrices. EdgeR was then used to estimate directional expression changes between conditions, which were compared with the major trends observed in the cell-level analysis.

### Reanalysis of public scRNA-seq dataset

To evaluate whether the major transcriptional trends observed in the in-house scRNA-seq dataset were supported by an independent PEDV infection dataset, we reanalyzed the public dataset GSE175411. This dataset contains jejunal cells from three-day-old piglets collected 24 h after oral infection with the PEDV variant AH2012/12 (GenBank: KU646831), together with age-matched uninfected controls. Raw FASTQ files were processed with Cell Ranger (10x Genomics) using standard pipelines, and the resulting gene expression matrices were imported into Seurat for downstream analysis. Cells with mitochondrial-gene proportions greater than 15% or fewer than 500 detected transcripts were excluded. Quality control, normalization, dimensionality reduction, clustering, cell-type annotation, and comparative analyses were performed in Seurat following the same overall workflow used for the in-house dataset. Downstream analyses focused on intestinal epithelial and myeloid subsets, including pathway-level transcriptional comparisons and epithelial–myeloid communication patterns. Selected downstream reanalysis and visualization were performed using the Omicsmart platform (Gene Denovo), which integrates publicly available bioinformatics analysis packages. The reanalysis was used to compare concordant transcriptional trends and epithelial–immune communication patterns between datasets.

### sEV RNA sequencing

sEVs were isolated from MOCK-treated, WIV PEDV-treated, and PEDV-infected IPEC-DQ cells. Total RNA was extracted from purified sEVs using TRIzol reagent (Invitrogen, USA). The extracted RNA was divided for miRNA and lncRNA library preparation.

### miRNA library preparation and analysis

Three microlitres of total RNA were used for small RNA library preparation using the KC-Digital™ Small RNA Library Prep Kit for Illumina® (Catalog No. DR08602, Wuhan Seqhealth Co., Ltd., China) according to the manufacturer’s instructions. This library preparation strategy incorporates an 8-nucleotide unique molecular identifier (UMI) to label individual small RNA molecules before PCR amplification, thereby reducing amplification bias and enabling accurate removal of PCR duplicates. The resulting cDNA libraries were size-selected by 6% polyacrylamide gel electrophoresis (PAGE), and fragments of approximately 160 bp were excised, purified, quantified using a Qubit 3.0 fluorometer (Thermo Fisher Scientific), and sequenced on an Illumina HiSeq X Ten platform in paired-end 150-bp (PE150) mode.

Raw sequencing reads were filtered to remove low-quality reads using FASTX-Toolkit (v0.0.13.2), and adapter sequences were trimmed using Cutadapt (v1.15). UMI-based deduplication was performed using in-house scripts to remove PCR duplicates and generate consensus reads. The deduplicated reads were aligned to the *Sus scrofa* reference genome (Sscrofa11.1, GCF_000003025.6) using Bowtie (v1.1.2). Known miRNAs were identified by alignment to miRBase, and candidate miRNAs not annotated in miRBase were predicted using miRDeep2 (v2.0.0.8). Differentially abundant miRNAs were identified using edgeR (v3.12.1), with a false discovery rate (FDR) ≤ 0.05 and |log₂ fold change| ≥ 1. Predicted target genes of differentially abundant miRNAs were subjected to GO and KEGG pathway enrichment analyses.

### lncRNA library preparation and analysis

For lncRNA sequencing, ribosomal RNA was depleted using the Ribo-off rRNA Removal Kit (Vazyme, China). Strand-specific libraries were subsequently constructed using the KC™ Digital Chain mRNA Library Preparation Kit (Wuhan Seqhealth Co., Ltd., China) according to the manufacturer’s instructions and sequenced on the DNBSEQ-T7 platform (BGI Intelligent Manufacturing) in paired-end 150-bp (PE150) mode.

Clean reads were mapped to the *Sus scrofa* reference genome (Sscrofa11.1, GCF_000003025.6), and transcript assembly was performed using StringTie (v1.3.2). Candidate lncRNA transcripts were retained according to the following criteria: transcript length ≥200 nt, at least two exons, supported by at least three mapped reads, and no overlap with annotated transcripts. Coding potential was evaluated using CPC2, CPAT (v1.2.4), CNCI (v2), and the Pfam database (v27.0). Transcripts predicted to be non-coding by all four methods were defined as novel lncRNAs. Differentially expressed lncRNAs were identified using edgeR (v3.12.1), with FDR ≤ 0.05 and |log₂ fold change| ≥ 1. Genes associated with differentially expressed lncRNAs were subjected to GO and KEGG pathway enrichment analyses.

### Macrophage transcriptome sequencing and analysis

Macrophages were treated with equal amounts of CD63-enriched sEVs derived from MOCK-treated, WIV PEDV-treated, or PEDV-infected IPEC-DQ cells. The amount of sEVs used for each treatment was normalized based on protein concentration determined by the bicinchoninic acid (BCA) assay. To investigate transcriptional responses of macrophages, total RNA was extracted using TRIzol reagent (Invitrogen, USA). RNA sequencing libraries were prepared, quantified using a Qubit 2.0 fluorometer (Thermo Fisher Scientific, USA), and sequenced on the Illumina HiSeq 2500 platform (Wuhan Seqhealth Co., Ltd., China). Raw sequencing reads were first processed using Trimmomatic (v0.36) to remove low-quality reads and trim adaptor-contaminated sequences. Clean reads were mapped to the *Sus scrofa* reference genome (Sscrofa11.1, GCF_000003025.6) using STAR (v2.5.3a) with default parameters. Reads mapped to exonic regions of each gene were counted using featureCounts (Subread v1.5.1; Bioconductor), and RPKM values were then calculated. Differentially expressed genes between groups were identified using edgeR (v3.12.1), with *P* < 0.05 and |log₂(fold change)| > 0.32 used as thresholds for statistical significance. GO and KEGG enrichment analyses of differentially expressed genes were performed using KOBAS (v2.1.1), with *P* < 0.05 considered statistically significant.

Macrophages were transduced with lentivirus overexpressing lncRNA TS2083 or the corresponding control vector. Total RNA was processed using the same library preparation, sequencing, and bioinformatic analysis workflow described above. Differentially expressed genes were identified and subjected to GO and KEGG pathway enrichment analyses. In parallel, ranked gene lists were used for GSEA, as described above.

### Macrophage functional assays

Macrophage functions were evaluated by assessing both migration and phagocytosis. For the migration assay, 3D4/21 cells or primary alveolar macrophages were seeded in the upper chambers of Transwell inserts, while lipopolysaccharide (LPS, 0.1 μg/mL) and complement component C5a (0.1 μM) were added to the lower chambers as chemotactic stimuli. After 24 h of incubation, non-migrated cells on the upper surface of the Transwell membrane were gently removed with a cotton swab. Cells that had migrated to the lower surface were fixed with 4% paraformaldehyde and stained with 0.1% crystal violet for 5–10 min at room temperature. The inserts were then washed thoroughly with phosphate-buffered saline (PBS), air-dried, and examined under a light microscope. For microscopy-based quantification, five randomly selected, non-overlapping fields were imaged from each Transwell insert under identical magnification and microscope settings. Migrated cells were quantified using ImageJ/Fiji after conversion to 8-bit grayscale and application of a consistent threshold. The Analyze Particles function was used to count migrated cells, and automated counts were manually checked to ensure accuracy. For each Transwell insert, five randomly selected, non-overlapping fields were imaged and averaged to generate one value per biological replicate.

Phagocytosis was assessed using carboxylated fluorescent microspheres^47,48^. Microspheres (117687-5, Polysciences) were opsonized in PBS containing 20% piglet serum under constant rotation at 4 °C for 16 h, centrifuged at 2000 × *g* for 5 min, washed, and resuspended in PBS at a final concentration of 1 × 10⁹ microspheres/mL. Macrophages were seeded in 24-well plates and serum-starved for 2 h at 37 °C to enhance phagocytic activity. Opsonized microspheres were then added and co-incubated with the cells for 20 min. After incubation, non-internalized microspheres were removed by washing with ice-cold PBS, and cells were fixed with 4% paraformaldehyde and counterstained with DAPI. Slides were mounted and analyzed by confocal laser scanning microscopy (CLSM-980, Carl Zeiss, France). For microscopy-based phagocytosis quantification, five randomly selected, non-overlapping fields were imaged from each biological replicate under identical microscope settings. Macrophages with clearly visible cell boundaries were analyzed, and bead uptake was quantified as bead-positive macrophages and/or bead number per macrophage, as indicated in the figure legends. Values from multiple fields were averaged to generate one value per biological replicate.

For flow-cytometric quantification of migrated macrophages, cells that migrated into the lower chamber of the Transwell system were collected, washed with PBS, and analyzed under identical acquisition settings. The number of migrated cells was determined by fixed-volume acquisition. For flow-cytometric analysis of phagocytosis, macrophages were incubated with fluorescent beads under the same conditions used for microscopy-based assays, thoroughly washed with ice-cold PBS to remove non-associated beads, and collected for analysis. Single macrophages were gated based on forward- and side-scatter profiles, and bead uptake was quantified as the percentage of bead-positive macrophages and/or bead-associated fluorescence intensity. For preliminary cross-species validation, THP-1 cells were differentiated into macrophage-like cells by treatment with phorbol 12-myristate 13-acetate (PMA; 100 ng/mL) for 24 h, followed by 24 h of rest in PMA-free medium before sEV treatment. THP-1-derived macrophage-like cells were then treated with MOCK-sEVs or PEDV-sEVs and analyzed using the same migration and bead-uptake assays described above.

For flow-cytometric quantification of F-actin, macrophages were treated under the same conditions used for the imaging assays, fixed with 1% paraformaldehyde, permeabilized with 0.1% Triton X-100, and stained with fluorescent phalloidin. After washing with PBS, cells were analyzed by flow cytometry under identical acquisition settings. Single cells were gated based on forward- and side-scatter profiles, and F-actin-associated fluorescence intensity was quantified.

IL-1β, IL-6, TNF-α, and PGE2 levels in epithelial conditioned media were measured using commercial ELISA kits according to the manufacturers’ instructions. To evaluate whether major soluble inflammatory cytokines contributed to PEDV-conditioned medium-induced macrophage dysfunction, conditioned medium was pre-incubated with neutralizing antibodies against IL-6 or TNF-α for 1 h at 37 °C before macrophage treatment. TNF-α neutralizing antibody (Alomone Labs, Jerusalem, Israel) was used at a final concentration of 500 ng/mL, and IL-6 neutralizing antibody (InvivoGen, San Diego, CA, USA) was used at a final concentration of 5 μg/mL. Matched IgG controls were used at the corresponding antibody concentrations. The antibody-treated conditioned medium was then applied to macrophages, and macrophage migration and bead uptake were assessed using the Transwell migration and phagocytosis assays described above.

### Confocal microscopy assays

For fluorescence in situ hybridization (FISH) and immunofluorescence (IF), macrophages were fixed with 4% paraformaldehyde and processed using probes for lncRNA TS2083 (Ribobio, China), U6 (GenePharm, China), and 18S (GenePharm, China) with a commercial FISH kit (F04401, GenePharm) following the manufacturer’s protocol. For IF staining, fixed cells were incubated with FITC-phalloidin for 30 min to label F-actin, washed with PBS, and then incubated overnight at 4 °C with rabbit anti-TMOD3 primary antibody (12521-1-AP, Proteintech; 1:200 dilution). After washing, cells were incubated for 30 min at room temperature with a fluorescently labeled secondary antibody (594-conjugated goat anti-rabbit IgG, 771384ES60, YEASEN; 1:200 dilution), washed again, and counterstained with DAPI (1 μg/mL, 5 min). Images were acquired using a Nikon A1 confocal microscope (Nikon, Japan). For combined RNA FISH and IF analysis of lncRNA TS2083 and ANXA2, macrophages were hybridized with the lncRNA TS2083 probe (RiboBio, China) and subsequently incubated overnight at 4 °C with mouse anti-ANXA2 monoclonal antibody (66035-1-Ig, Proteintech; 1:200), followed by Alexa Fluor 594-conjugated goat anti-mouse IgG (33212ES60, YEASEN; 1:200). Nuclei were counterstained with DAPI. For tissue-level assays, jejunal segments from piglets were embedded in OCT compound, cryosectioned, and analyzed using a CD68 probe (Sangon Biotech, China) with an in situ hybridization kit (F04401, GenePharm). Sections were subsequently incubated with anti-PEDV N protein monoclonal antibody (Jinsirui Biotechnology; 1:200 dilution), and IF staining was carried out as described for cellular samples.

For time-lapse confocal imaging, macrophages were seeded in 35-mm confocal dishes, transfected with miR-190a mimic at 40–50% confluence, and imaged using an LSM 980 confocal microscope with ZEN software (Carl Zeiss, Germany). Imaging was performed at 30-s intervals for 30 min, and LPS (1 μg/mL) was added at the start to enhance random migration. Cell trajectories were tracked in real time, and migration paths of 10–50 randomly selected cells were analyzed using the manual tracking plugin in ImageJ, with quantitative parameters calculated using Python scripts. For live-cell imaging of F-actin dynamics, macrophages transfected with miR-190a mimic or the corresponding control mimic were stained with ActinGreen™ 488 ReadyProbes™ (R37110, Thermo Fisher Scientific; 1:100 dilution) for 1 h, washed with PBS, and maintained in phenol red-free Opti-MEM. LPS (1 μg/mL) was added immediately before imaging. Time-lapse images were acquired using an LSM 980 confocal microscope with ZEN software at 10-s intervals for 20 min under identical acquisition settings. For representative kinetic analysis, fluorescence intensity was measured in a selected ventral region of interest (ROI) in one representative cell per condition following background subtraction. Fluorescence intensity at each time point (F_t) was normalized to the intensity at 0 min (F_0) and expressed as F_t/F_0, generating one representative trace per condition.

### Luciferase assay

Wild-type (WT) and mutant (MUT) pmirGLO reporter plasmids containing the TMOD3 3′-UTR were purchased from GenePharma (Shanghai, China). HEK293T cells were co-transfected with the reporter plasmids and either miR-190a mimic or negative control mimic using Lipofectamine 3000 (Invitrogen, USA). At 24 h after transfection, firefly and Renilla luciferase activities were measured using the Dual Luciferase Reporter Gene Assay Kit (RG027, Beyotime, Shanghai, China) according to the manufacturer’s instructions. Firefly luciferase activity was normalized to Renilla luciferase activity. Each experiment was performed in triplicate and repeated independently at least three times.

### Western blotting

Cells were lysed with protease-inhibitor-containing lysis buffer. Equal amounts of protein were separated by SDS-PAGE and transferred onto PVDF membranes. After blocking with 5% bovine serum albumin (BSA) in Tris-buffered saline containing 0.1% Tween 20 (TBST) for 1 h at room temperature, the membranes were incubated overnight at 4 °C with the following primary antibodies: rabbit anti-TMOD3 (12521-1-AP, Proteintech; 1:200) and mouse anti-ANXA2 monoclonal antibody (66035-1-Ig, Proteintech; 1:200). Following three washes with TBST, the membranes were incubated for 1 h at 37 °C with the corresponding HRP-conjugated secondary antibodies (1:5000). Antibody binding was detected using an enhanced chemiluminescence (ECL) system. Band densities were quantified and normalized to β-actin. A similar protocol was employed to detect sEV-associated proteins using specific antibodies against anti-CD63 monoclonal antibody (ab134045, Abcam), anti-CD81 monoclonal antibody (ab109201, Abcam), anti-Calnexin monoclonal antibody (ab133615, Abcam) and anti-HSP70 polyclonal antibody (ab181606, Abcam). For sEV Western blotting, either equal volumes of sEV preparations recovered from equal starting volumes of conditioned medium or equal amounts of sEV protein were loaded, as indicated in the figure legends. To further compare marker abundance under normalized loading conditions, equal amounts of sEV protein were loaded in the additional validation experiment. CD63, CD81, and HSP70 were detected as positive sEV-associated markers, and Calnexin was detected as a negative marker for endoplasmic reticulum contamination. Whole-cell lysates were used as positive controls for Calnexin. Band intensities were quantified using ImageJ and expressed relative to MOCK-sEVs (Figs. 2D, 5F, 7E, J, L, N and Fig. S5B). Source data are provided as a Source Data file.

### Quantitative reverse-transcription PCR (RT-qPCR)

To quantify viral load, IPEC-DQ cells were seeded into 12-well plates one day prior to infection and incubated at 37 °C with 5 % CO₂ overnight. When the monolayer reached 70% confluence, cells were inoculated with PEDV at an MOI of 1 and incubated at 37 °C for 1 h to allow virus adsorption. After removal of the inoculum, cells were gently washed three times with PBS to eliminate unbound virions, then maintained in DMEM/F-12 supplemented with 2% FBS. At the indicated time points post-infection, cytopathic effects were monitored by inverted microscopy and cell samples were harvested for RT-qPCR analysis.

Total RNA was extracted from sEVs and cells using TRIzol reagent (Invitrogen, USA). For mRNA and lncRNA detection, reverse transcription was performed with HiScript III RT SuperMix (Vazyme, China), and quantitative real-time PCR was carried out on an ABI 7500 system (Life Technologies, USA). Gene expression levels were calculated using the comparative Ct method and normalized to GAPDH. Specific primers for PEDV, miR-190a target genes, lncRNA TS2083, and ANXA2 are listed in Supplementary Table 2.

For miRNA quantification, total RNA from sEV samples was extracted with TRIzol reagent, and 10 μM cel-miR-39-3p was added as a spike-in control prior to lysis. RNA concentration and purity were measured with a NanoDrop spectrophotometer. First-strand cDNA was synthesized using the Tailing Reaction Kit (Sangon Biotech, China), in which Poly(A) tailing and reverse transcription were performed simultaneously. qPCR was conducted with the TB Green Premix Ex Taq kit (TaKaRa, China) on an Applied Biosystems 7500 Fast Real-Time PCR System. MiRNA sequences are provided in Supplementary Table 2.

### RNA–protein and protein–protein interaction assays

The cDNA of lncRNA TS2083 (sense and antisense strands; SynbioB, China) was transcribed in vitro using the T7 High Yield RNA Transcription Kit (TR101, Vazyme, China). The RNA transcripts were biotinylated using the Pierce™ Magnetic RNA–Protein Pull-Down Kit (Thermo Fisher Scientific, USA), incubated with RAW 264.7 cell lysates, and processed according to the manufacturer’s instructions. Proteins bound to the biotinylated RNA were eluted, quantified, and subjected to mass spectrometry and Western blot analysis to identify candidate interactors of lncRNA TS2083.

To test whether ANXA2 binds to lncRNA TS2083, RIP assays were carried out with the BersinBio RIP RNA-Binding Protein Immunoprecipitation Kit (Bes5101, BersinBio, China). Macrophages were lysed in RIP lysis buffer containing protease and RNase inhibitors. Cell lysates were incubated overnight at 4 °C with magnetic beads conjugated with IgG (negative control) or anti-ANXA2 monoclonal antibody (66035-1-Ig, Proteintech). Immunoprecipitated RNA was purified and detected by qRT-PCR using primers specific to lncRNA TS2083.

### Co‑immunoprecipitation (Co‑IP)

Pre-treated macrophages were lysed in RIP lysis buffer containing protease inhibitors. The lysate supernatant was collected following centrifugation. Subsequently, the lysate supernatant was incubated with magnetic beads pre-coupled with IgG (negative control), anti-myc antibody, or anti-ANXA2 monoclonal antibody at 4 °C for 12 h. After incubation, the bead-antibody-protein complexes were stringently washed four times with IP wash buffer to remove non-specifically bound proteins. Finally, bound protein complexes were eluted for subsequent Western blot analysis (Fig. 7O). Source data are provided as a Source Data file.

### In vivo evaluation of PEDV-induced sEVs on intestinal macrophage function

To assess the effects of PEDV-induced sEVs on intestinal macrophage migration and phagocytosis, six healthy 10-day-old piglets were randomly assigned to two groups (*n* = 3 per group). Piglets in the experimental group received an intravenous injection (via the marginal ear vein) of sEVs (50 μg/mL) derived from PEDV-infected intestinal epithelial cells, whereas control piglets received an equivalent dose of sEVs from PBS-treated epithelial cells. After 24 h of routine housing, all piglets underwent intestinal loop assays with mCherry-labeled *Escherichia coli* (1 × 10⁸ CFU/mL). Under anesthesia, the jejunum was exposed and ligated to form a closed loop into which the bacterial suspension was injected. Two hours later, piglets were euthanized, and ligated jejunal segments were collected. Intestinal macrophages were labeled in situ using a CD68-specific probe, and images were acquired with a Nikon A1 confocal microscope (Nikon, Japan).

In parallel, to determine whether characteristic sEVs could be detected in circulation, another six healthy 10-day-old piglets were randomly assigned to two groups (*n* = 3 per group). Piglets in the experimental group were orally inoculated with 1 mL of PEDV suspension (1 × 10⁷ PFU/mL), while control piglets received an equal volume of PBS. At 24 h post-inoculation, whole blood was collected and centrifuged at 2000 × *g* for 20 min to obtain serum. Serum-derived sEVs were isolated by ultracentrifugation followed by anti-CD63 immunomagnetic enrichment, and the expression levels of miR-190a and lncRNA TS2083 within serum-derived sEV preparations were determined by qPCR. For tissue imaging analyses, three non-consecutive sections were analyzed from each piglet, and three randomly selected, non-overlapping fields were imaged from each section. Quantitative values from individual fields were first averaged within each section and then averaged within each animal. The animal-level mean was used as the statistical unit. Microscopic fields and tissue sections were treated as technical subsampling and were not used as independent biological replicates.

### Cytotoxicity assay

Cytotoxicity of CHX and MG132 was assessed using the CCK-8 assay. Macrophages were seeded in 96-well plates and cultured overnight to 90% confluence. Cells were then treated with graded concentrations of CHX (1–500 µg/mL) or MG132 (1–50 µM) for 12 h, including blank and negative controls. After drug exposure, cells were gently washed with DMEM and replenished with fresh medium containing 10 µL CCK-8 reagent per well, followed by a 2 h incubation. Absorbance was measured at 450 nm using a microplate reader, and relative cell viability was calculated as a percentage of the untreated control.

### Imaging quantification

For microscopy-based quantification, multiple randomly selected, non-overlapping fields were acquired under identical imaging settings within each independently performed experiment. Field-level measurements were treated as technical subsamples and averaged to generate one experiment-level value for statistical analysis. For animal tissue imaging, section- and field-level measurements were averaged within each piglet, and each individual piglet was considered one biological replicate.

### Statistical analysis

Results are presented as mean ± standard deviation (SD). Statistical analyses were performed using SPSS 17.0 software (SPSS Inc., Chicago, IL, USA) and GraphPad Prism v9.0.0. Comparisons between two groups were performed using an unpaired two-tailed Student’s *t* test, and comparisons among multiple groups were performed using one-way ANOVA. For in vitro experiments, technical measurements such as microscopic fields, wells, or individual cells collected within the same independent biological experiment were averaged to generate one biological replicate-level value. Statistical comparisons were performed using independent biological replicate-level values unless otherwise stated. For animal experiments, each piglet was considered a single biological replicate. For plots with fewer than ten independent observations, all independent data points are shown. Unless otherwise indicated, experiments were performed with at least three independent biological replicates. Significance was set at *P < 0.05 and **P < 0.01. Investigators responsible for data collection and quantitative analysis were blinded to group allocation. Samples and image files were assigned coded identifiers, and group identities were revealed only after the analyses had been completed.

### Reporting summary

Further information on research design is available in the Nature Portfolio Reporting Summary linked to this article.

## Supplementary information

Supplementary Information

Description of Additional Supplementary Files

Supplemental Movie 1

Supplemental Movie 2

Reporting Summary

Transparent Peer Review file

## Source data

Source Data

## Data Availability

The scRNA-seq data from the intestinal mucosa of both healthy and PEDV-infected piglets have been deposited in the NCBI Gene Expression Omnibus (GEO) under the accession number GSE306895. The whole-transcriptome sequencing data of sEVs derived from intestinal epithelial cells of the control, WIV PEDV-treated, and PEDV-infected groups, together with the RNA-seq data of macrophages treated with these sEVs, are available in GEO under accession numbers GSE305997 and GSE305999. Furthermore, the transcriptome sequencing data for macrophages treated with lncRNA TS2083 have also been uploaded to the Sequence Read Archive (SRA) under accession PRJNA1309073. The mass spectrometry proteomics data for the lncRNA TS2083 interactome generated using RAW 264.7 cell lysates have been deposited with the ProteomeXchange Consortium via the iProX partner repository under dataset identifier PXD069460. Source Data are provided with this paper. All data supporting the findings of this study are available within the article, its Supplementary Information files, the Source Data file, and the public repositories listed above. Source data are provided with this paper.

## Code availability

Author-accessible scripts used for omics-related analyses in this study are publicly available on GitHub: https://github.com/apanhui/scRNA_hsa. The repository includes a detailed README.md file with execution instructions and workflow descriptions. Other platform-based omics analyses without author-generated custom code were performed as described in the Methods.
