# Supplementary material for: Small extracellular vesicle-mediated epithelial–macrophage crosstalk contributes to macrophage dysfunction during PEDV infection
Source: Nat Commun. 2026 Sep 18;17:11548. doi: 10.1038/s41467-026-77854-w (PMC13748656; doi:10.1038/s41467-026-77854-w)
Supplement: Supplementary file 1 [file 41467_2026_77854_MOESM1_ESM.pdf]

Supplementary information

Small extracellular vesicle-mediated epithelial–macrophage crosstalk contributes to macrophage dysfunction during PEDV infection

Qiu Zhong[1], Chengcheng Wang[1], Ruiling Liu[1], Yunlei Cao[1], Hui Zeng[1], Na Su[1], Rongfeng Tang[1], Wenqian Wang[1], Jiaxin Qi[1], Yuchen Li[1*], Qian Yang[1]

*Corresponding author: Yuchen Li; yuchenli2022@njau.edu.cn

This PDF file includes:

Figures S1 to S9

Other supplementary materials for this manuscript include the following:

Supplementary Movie 1

Supplementary Movie 2

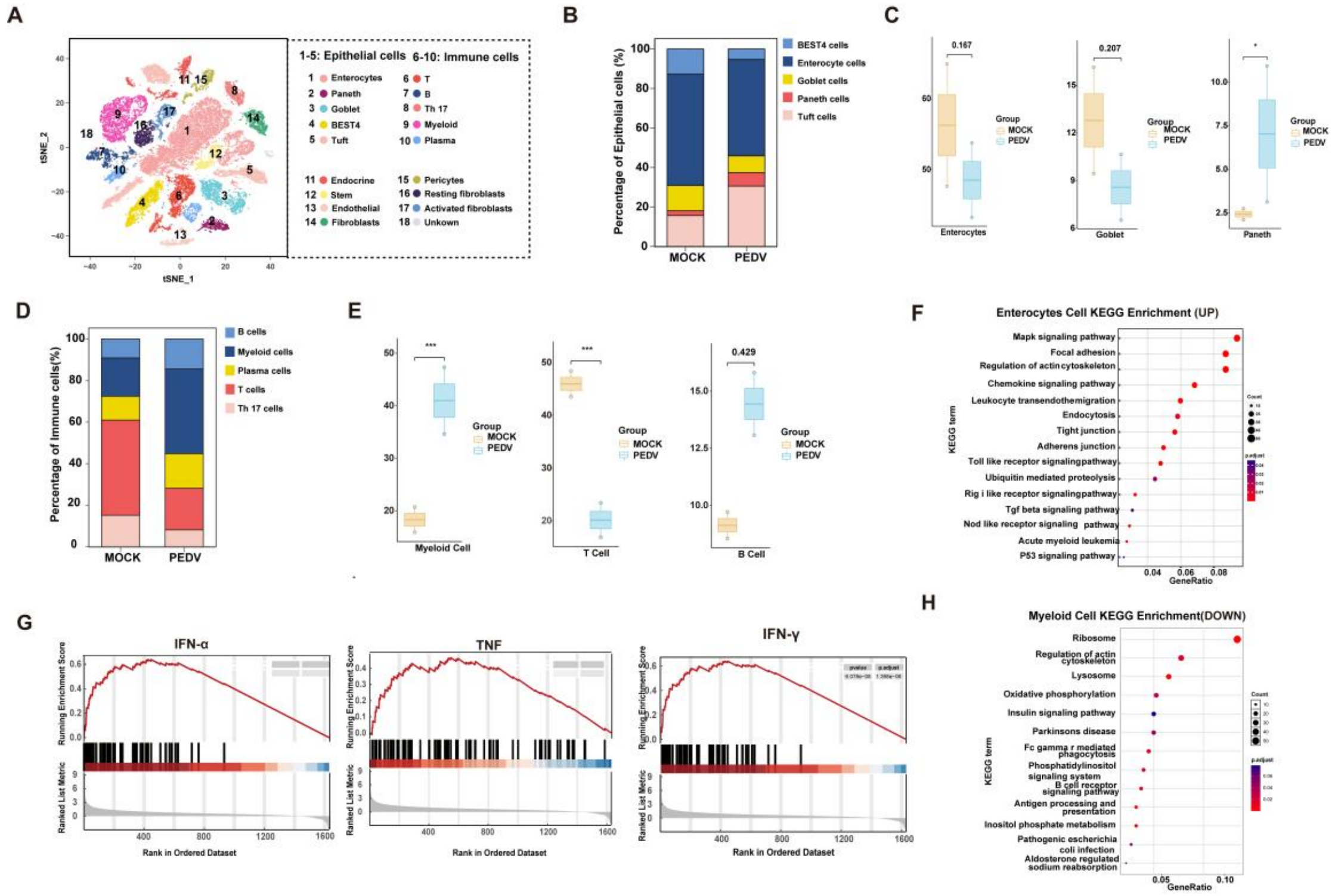

Fig. S1 Single-cell transcriptomic profiling of intestinal tissues from PEDV-infected piglets. (A) UMAP visualization. (B, D) Proportions of epithelial cell subsets (B) and immune cell subsets (D) in the jejunal mucosa of MOCK- and PEDV-infected piglets. (C, E) Relative cell-type composition and ROE summaries of major intestinal cell populations across conditions. ROE values were calculated from the observed and expected cell numbers for each cell population and used to visualize relative enrichment or depletion patterns. (F) KEGG enrichment analysis of upregulated genes in epithelial cells from PEDV-infected tissues. (G) GSEA enrichment plots of cytokine-related pathways, including IFN-α, IFN-γ, and TNF-α in epithelial cells after PEDV infection. (H) KEGG enrichment analysis of downregulated genes in jejunal myeloid cells after PEDV infection.

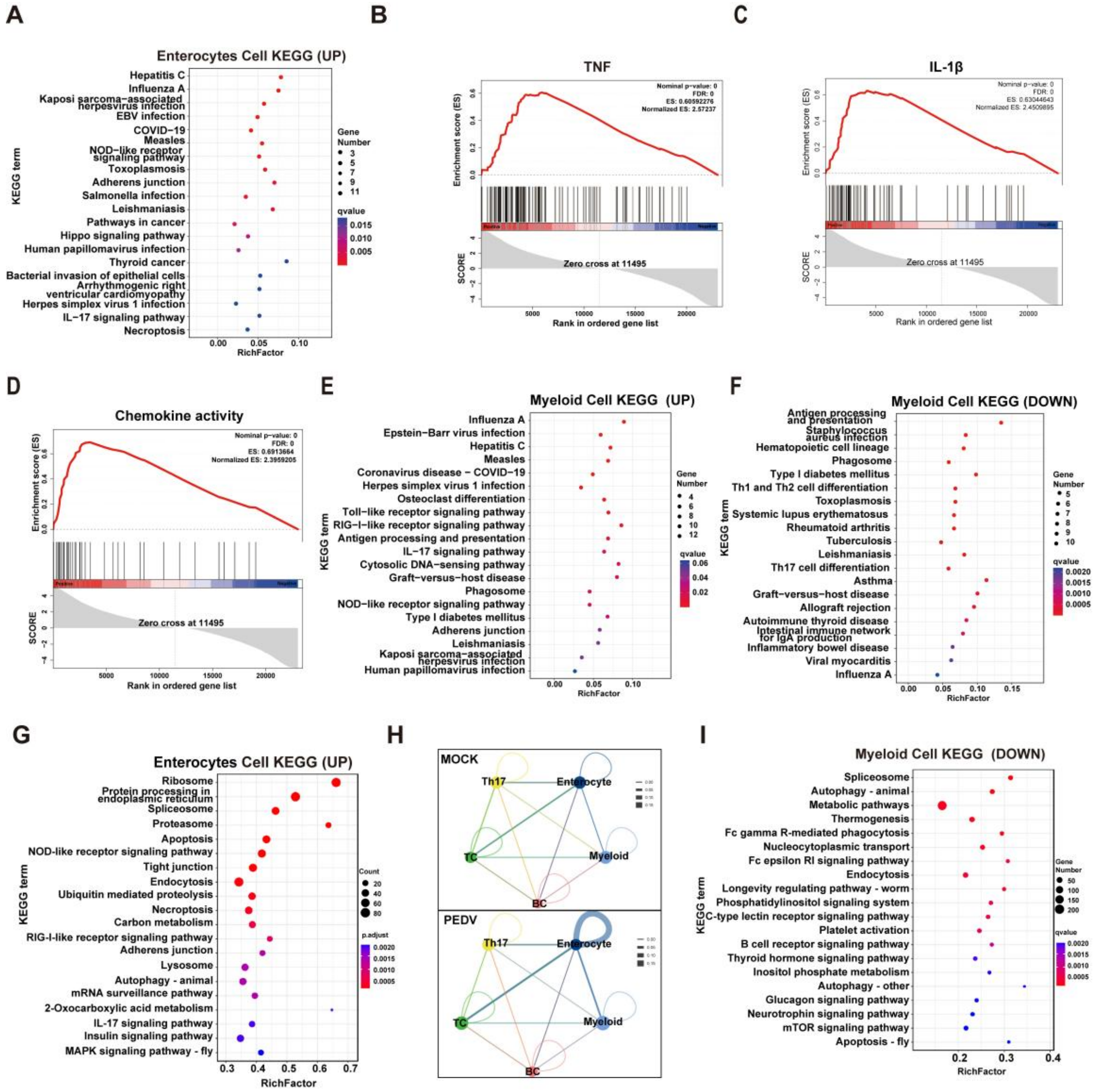

Fig. S2 Sample-aware pseudobulk analysis shows directional concordance with major single-cell transcriptional trends. (A-F) Sample-level pseudobulk analysis shows directional concordance with major single-cell transcriptional trends. (A) KEGG enrichment analysis of upregulated genes in epithelial cells from PEDV-infected tissues. (B-D) GSEA enrichment plots of cytokine-related pathways, including TNF (B), IL-1β (C), and Chemokine activity (D) in epithelial cells after PEDV infection. (E-F) KEGG enrichment analysis of upregulated (E) and downregulated (F) genes in jejunal myeloid cells after PEDV infection. (G–I) Validation analysis using a published dataset from piglets orally infected with another PEDV variant (AH2012/12, GenBank: KU646831). (G) KEGG enrichment analysis of upregulated genes in epithelial cells. (H) Ligand–receptor interaction strength between epithelial cells and myeloid populations (macrophages, DCs, monocytes). (I) KEGG enrichment analysis of downregulated genes in intestinal myeloid cells.

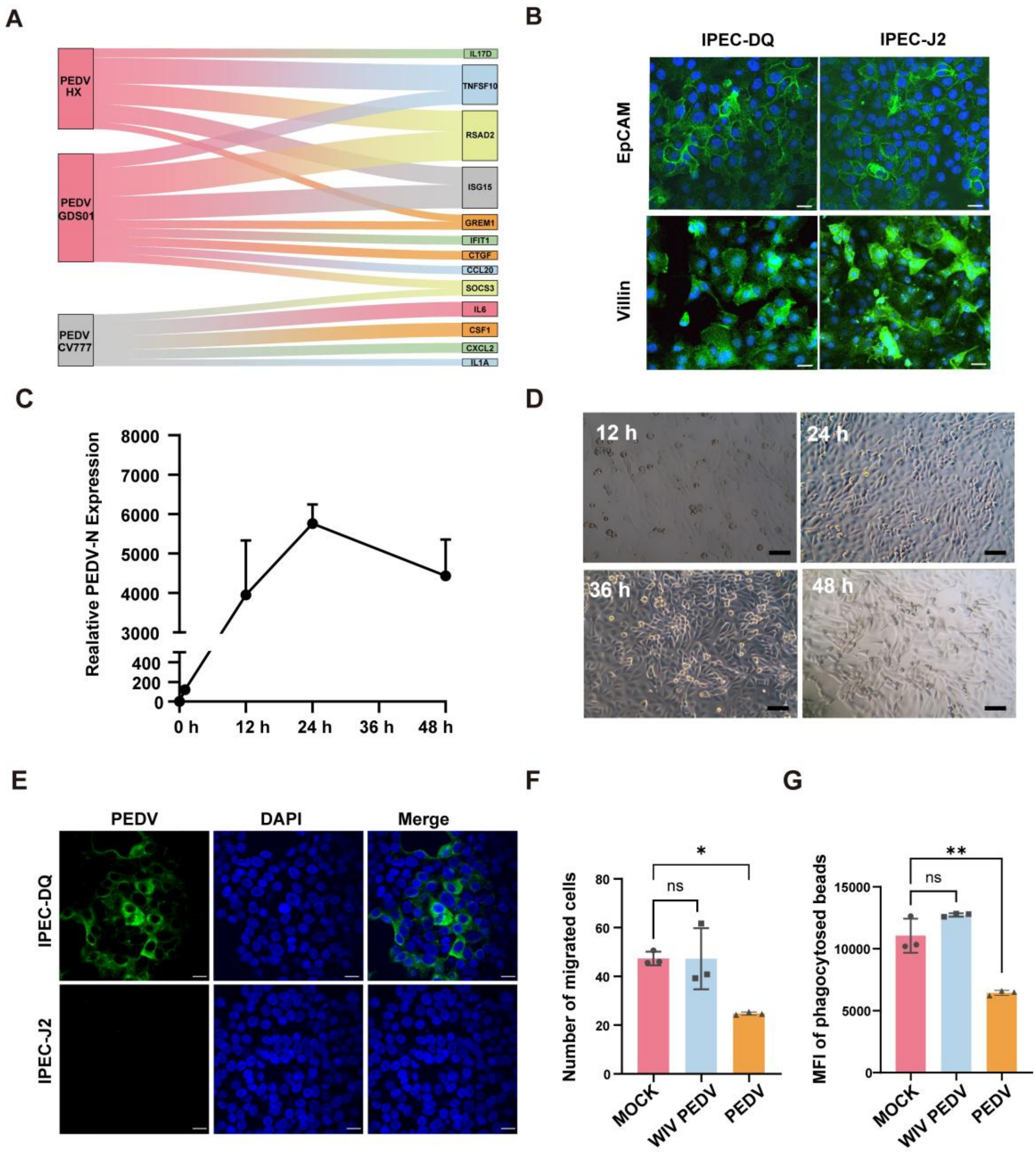

Fig. S3 PEDV infection induces inflammatory responses in intestinal epithelial cells. (A) Sankey plot showing secretion of cytokines and chemokines in intestinal epithelial cells infected with classical and variant PEDV strains. (B–E) Characterization of PEDV infection and cellular responses in IPEC-DQ cells. (B) Immunofluorescence staining of epithelial markers EpCAM and villin in IPEC-DQ and parental IPEC-J2 cells. Nuclei were counterstained with DAPI. Scale bar, 20 µm. IPEC-DQ cells were inoculated with PEDV, and samples were collected at different time points. (C) RT–qPCR quantification of PEDV N gene expression. (D) Representative light microscopy images showing morphological changes of IPEC-DQ cells. Scale bar, 50 µm. (E) Immunofluorescence detection of PEDV antigen in IPEC-DQ and IPEC-J2 cells after

PEDV infection. PEDV antigen is shown in green, and nuclei were counterstained with DAPI. Scale bar, 20 μm. (F, G) Flow-cytometric validation of the effects of conditioned medium from MOCK-, WIV PEDV-, or PEDV-treated epithelial cells on macrophage function. (F) Quantification of migrated macrophages, $n = 3$ biologically independent samples. (G) Quantification of bead uptake, shown as mean fluorescence intensity (MFI) ($n = 3$ biologically independent samples). Data are presented as mean ± SD from $n = 3$ independent experiments. Images shown in (B-E) are representative of three independent biological replicates. Statistical significance was determined by one-way ANOVA with Tukey's post-hoc test, with *P*-values denoted as follows: $^{*}P < 0.05$, $^{**}P < 0.01$. Exact *P* values are available in the source data.

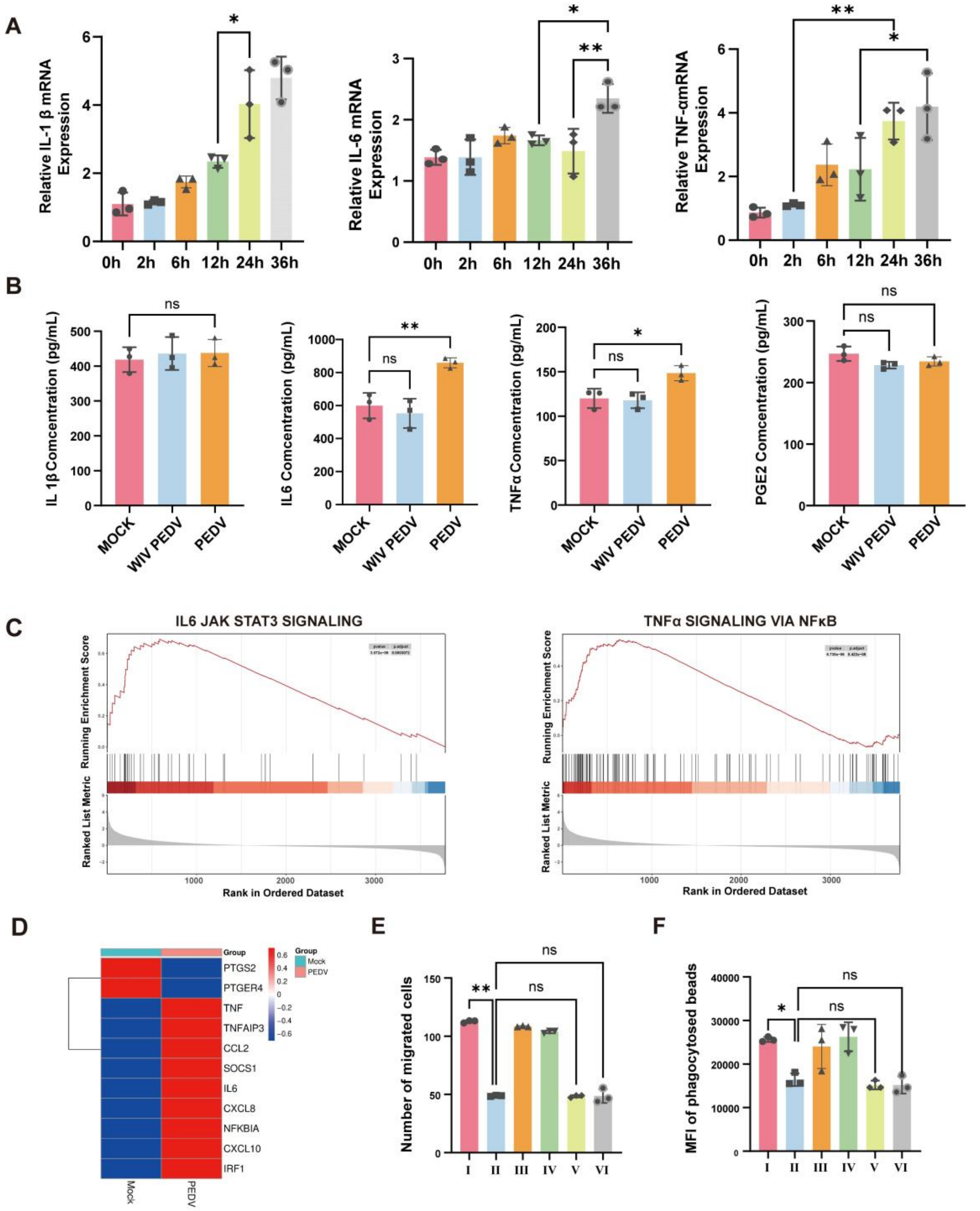

Fig. S4 Inflammatory activation does not fully explain PEDV-conditioned medium-induced macrophage dysfunction. (A) RT-qPCR analysis of IL-1β, IL-6, and TNF-α mRNA expression in intestinal epithelial cells at the indicated time points after PEDV infection. Expression levels were normalized to GAPDH, $n = 3$ biologically independent samples. (B) ELISA quantification of IL-1β, IL-6, TNF-α, and PGE2 concentrations in conditioned media from MOCK-, WIV PEDV-, or PEDV-treated

intestinal epithelial cells, $n = 3$ biologically independent samples. (C) Gene set enrichment analysis showing enrichment of IL-6/JAK/STAT3 signaling and TNF-α/NF-κB signaling signatures in macrophages treated with PEDV-conditioned medium. (D) Heatmap showing representative inflammatory-response genes in macrophages treated with MOCK- or PEDV-conditioned medium. (E, F) Effects of IL-6 or TNF-α neutralization on PEDV-conditioned medium-induced macrophage dysfunction. MOCK- or PEDV-conditioned medium was pre-incubated with matched IgG control, anti-IL-6 neutralizing antibody, or anti-TNF-α neutralizing antibody before macrophage treatment. Macrophage migration was quantified by flow cytometry (E), and bead uptake was quantified as bead-associated mean fluorescence intensity (MFI) (F), $n = 3$ biologically independent samples. Groups are defined as follows: I, MOCK-CM + IgG; II, PEDV-CM + IgG; III, MOCK-CM + anti-IL-6; IV, MOCK-CM + anti-TNF-α; V, PEDV-CM + anti-IL-6; VI, PEDV-CM + anti-TNF-α. Data are presented as mean ± SD from $n = 3$ independent experiments. Statistical significance was determined by one-way ANOVA with Tukey's post-hoc test, with *P*-values denoted as follows: $^{*}P < 0.05$, $^{**}P < 0.01$. Exact *P* values are available in the source data.

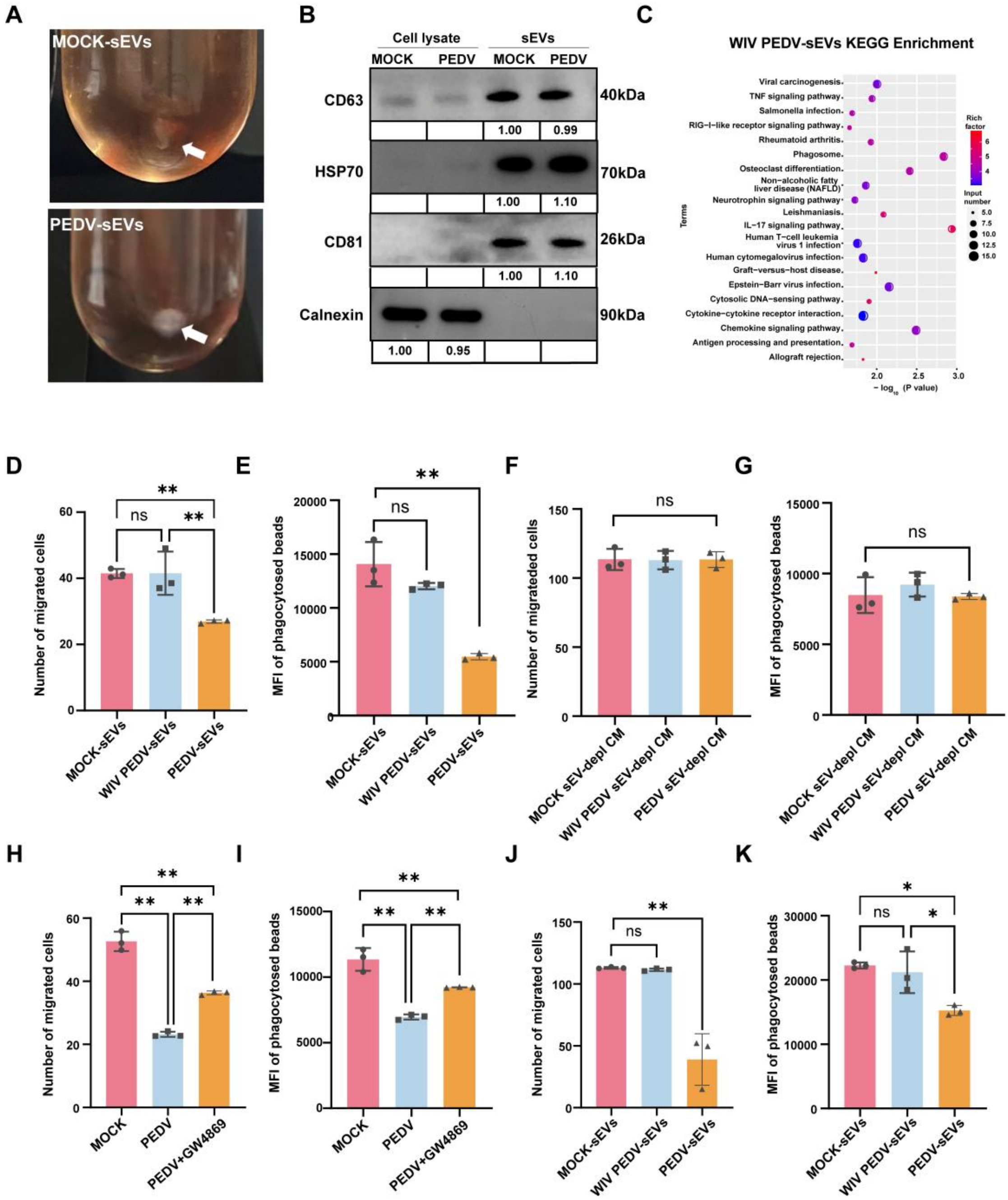

Fig. S5 Characterization and functional validation of epithelial sEVs and sEV-depleted conditioned medium. (A) Representative image of ultracentrifugation pellets showing sEV deposition at the bottom of tubes from PEDV-infected IPEC-DQ cell supernatants (arrows indicate sEV pellets). (B) Western blot analysis of sEV marker enrichment and Calnexin-negative characterization. Equal amounts of sEV protein were loaded for MOCK-sEV and PEDV-sEV preparations. Matched donor-cell lysates were included for comparison. CD63, CD81, and HSP70 were enriched in sEV

preparations, whereas Calnexin was detected in donor-cell lysates but not in sEV preparations. (C) KEGG enrichment analyses of downregulated genes in macrophages treated with sEVs from WIV PEDV-infected epithelial cells. (D, E) Flow-cytometric validation of macrophage migration and bead uptake after treatment with BCA-normalized MOCK-, WIV PEDV-, or PEDV-sEV preparations. (D) Quantification of migrated macrophages by flow cytometry. (E) Quantification of bead uptake by flow cytometry, shown as mean fluorescence intensity. (F-G) Flow-cytometric functional analysis of sEV-depleted conditioned medium. Conditioned media from MOCK-, WIV PEDV-, or PEDV-treated epithelial cells were subjected to ultracentrifugation to remove the sEV fraction, and the resulting sEV-depleted conditioned media were applied to macrophages. (F) Quantification of migrated macrophages by flow cytometry. (G) Quantification of bead uptake by flow cytometry, shown as mean fluorescence intensity. (H, I) Flow-cytometric validation of macrophage migration and bead uptake after treatment with conditioned medium from GW4869-treated donor epithelial cells. (J, K) Preliminary validation of PEDV-sEV effects on THP-1-derived human macrophage-like cells. (J) Flow-cytometric quantification of migrated cells after treatment with MOCK-sEVs, WIV PEDV-sEVs or PEDV-sEVs. (K) Flow-cytometric quantification of bead uptake after treatment with MOCK-sEVs, WIV PEDV-sEVs or PEDV-sEVs, shown as mean fluorescence intensity. Data are presented as mean ± SD from $n = 3$ independent experiments. Images shown in (A-B) are representative of three independent biological replicates. Statistical significance was determined by one-way ANOVA with Tukey's post-hoc test, with P-values denoted as follows: $*P < 0.05$, $**P < 0.01$. Exact P values are available in the source data.

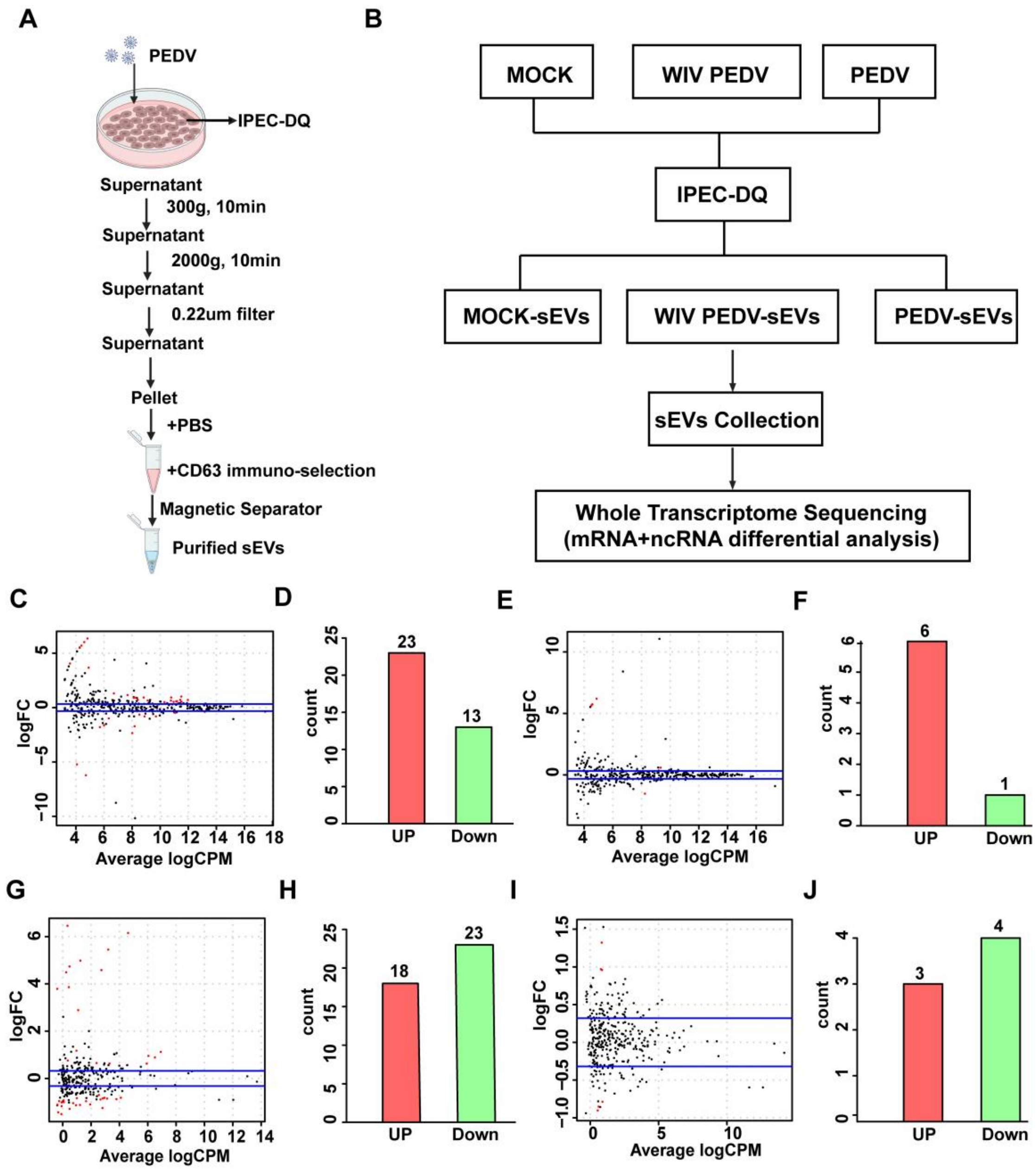

Fig. S6 Transcriptomic features of miRNAs and lncRNAs in PEDV-induced epithelial sEVs. (A) Workflow for sEV isolation and purification from PEDV-infected IPEC-DQ cell supernatants, including differential centrifugation, filtration, ultracentrifugation, and anti-CD63 immunomagnetic enrichment. (B) Experimental grouping for sEV-associated transcriptome sequencing, including MOCK, UV-inactivated PEDV (WIV PEDV), and PEDV-infected groups. (C, D) Differential expression analysis of miRNAs between MOCK and PEDV groups. (C) MA plot of logFC vs average logCPM. (D) Bar plot showing numbers of upregulated (red) and downregulated (green) miRNAs. (E, F) Differential expression analysis of miRNAs

between MOCK and WIV PEDV groups. (E) MA plot. (F) Bar plot showing numbers of upregulated and downregulated miRNAs. (G, H) Differential expression analysis of lncRNAs between MOCK and PEDV groups. (G) MA plot. (H) Bar plot showing numbers of upregulated and downregulated lncRNAs. (I, J) Differential expression analysis of lncRNAs between MOCK and WIV PEDV groups. (I) MA plot. (J) Bar plot showing numbers of upregulated and downregulated lncRNAs. Schematic in (A): Created in BioRender. Qin, X. (2026) https://BioRender.com/u2xdpiu.

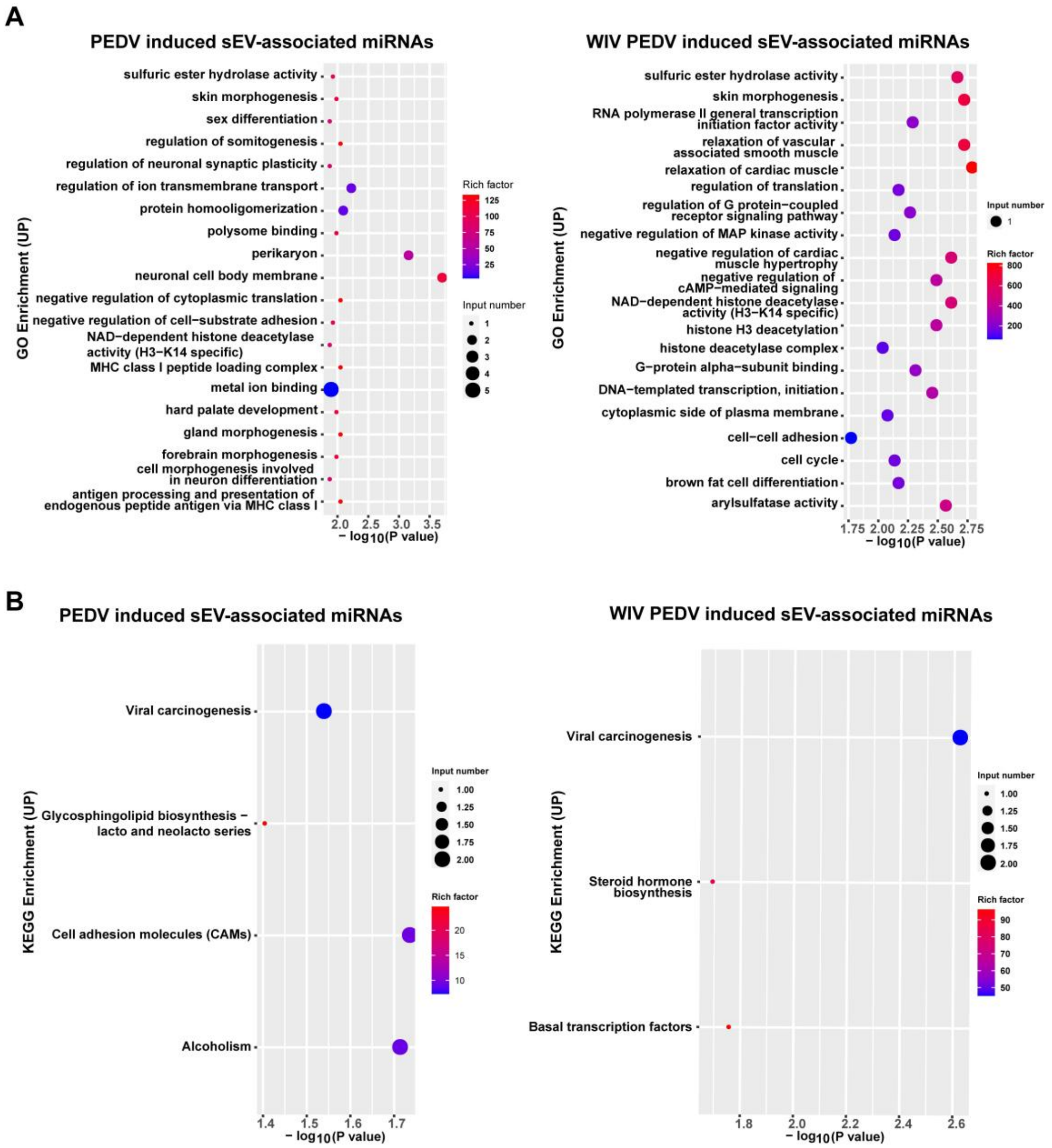

Fig. S7 GO and KEGG enrichment analyses of predicted target genes of differentially abundant sEV-associated miRNAs. (A, B) Predicted target genes of differentially abundant miRNAs in PEDV-sEVs and WIV PEDV-sEVs were subjected to GO and KEGG enrichment analyses. (A) GO enrichment analysis showing representative biological process, molecular function, and cellular component terms. (B) KEGG enrichment analysis showing representative enriched pathways, including pathways related to cell adhesion, glycosphingolipid biosynthesis, and transcriptional regulation. GO, Gene Ontology; KEGG, Kyoto Encyclopedia of Genes and Genomes.

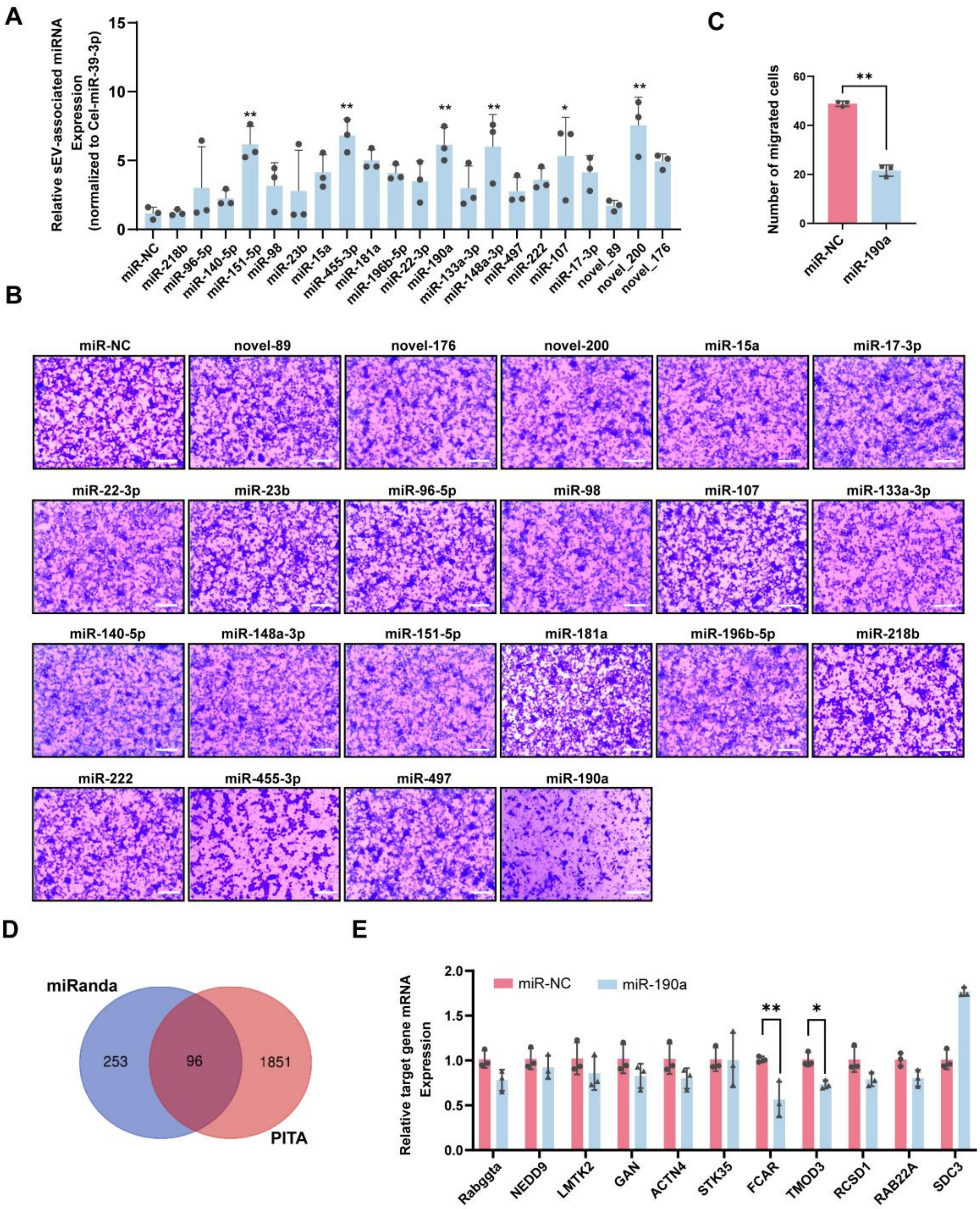

Fig. S8 Identification of migration-suppressive miRNAs and predicted targets in PEDV-induced epithelial sEVs. (A) RT–qPCR analysis of candidate miRNAs potentially involved in actin cytoskeleton regulation in sEVs derived from PEDV-infected epithelial cells, $n = 3$ biologically independent samples. (B) Representative images of Transwell migration assays showing macrophage migration after transfection with synthetic mimics of candidate miRNAs. Scale bar, 50 μm. Images are representative of three independent replicates. (C) Flow-cytometric validation of the

inhibitory effect of miR-190a on macrophage migration, $n = 3$ biologically independent samples. (D) Venn diagram of miR-190a target gene prediction using the miRanda and PITA algorithms, with 96 overlapping genes identified. (E) RT–qPCR analysis of candidate target genes following miR-190a overexpression in macrophages, $n = 3$ biologically independent samples. TMOD3 expression was significantly reduced, identifying it as a direct candidate target. Data are presented as mean ± SD from $n = 3$ independent experiments. Statistical significance was determined by one-way ANOVA with Šídák's multiple comparisons test, with P-values denoted as follows: *$P < 0.05$, **$P < 0.01$. Exact P values are available in the source data.

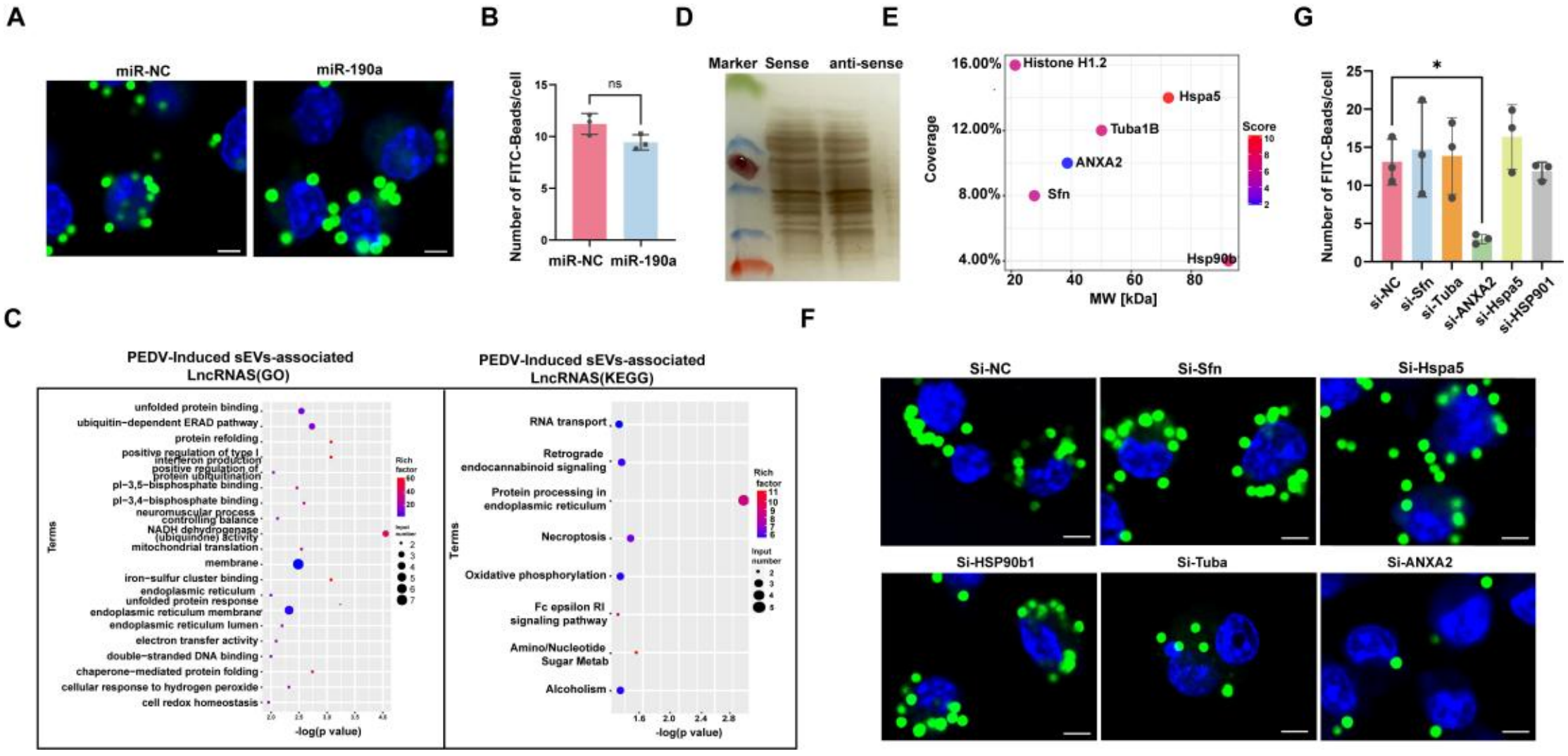

Fig. S9 sEV-associated lncRNA expression landscape and functional characterization of lncRNA TS2083-binding proteins. (A) Confocal microscopy showing phagocytosis of fluorescent beads (green) by macrophages after transfection with miR-190a mimic or control mimic; nuclei are counterstained with DAPI (blue). Scale bar, 5 µm. (B) Quantification of bead uptake per macrophage shown in (A, *n* = 3 biologically independent samples). For each biological replicate, five randomly selected, non-overlapping fields were imaged and averaged to generate one value per biological replicate. (C) GO and KEGG enrichment analysis of genes associated with differentially abundant sEV-associated lncRNAs. (D–E) Identification of candidate lncRNA TS2083–interacting proteins by RNA pull-down coupled with mass spectrometry using RAW 264.7 cell lysates. (D) SDS–PAGE followed by silver staining of proteins enriched by sense or antisense lncRNA TS2083 probes. (E) Mass spectrometry results showing representative high-confidence lncRNA TS2083–interacting proteins (coverage vs molecular weight). (F) Confocal microscopy showing phagocytosis of fluorescent beads (green) by macrophages after siRNA knockdown of candidate binding proteins; nuclei are counterstained with DAPI (blue). Scale bar, 5 µm. (G) Quantification of bead uptake per macrophage shown in (F, *n* = 3 biologically independent samples). For each biological replicate, five randomly selected, non-overlapping fields were imaged and averaged to generate one value per biological replicate. Data are presented as mean ± SD from n = 3 independent experiments. Images shown in (A, F) are representative of three independent biological replicates. Statistical significance was determined by one-way ANOVA with Tukey's post-hoc test, with *P*-values denoted as follows: *$P < 0.05$, **$P < 0.01$. Exact *P* values are available in the source data.

Table S1 Sequences of miRNA mimics, inhibitors, and siRNAs used in this study

Oligonucleotide	Guide strand	Sequence (5′–3′)
miR-190a		TGATATGTTTGATATATTAGG
miR-15a		TAGCAGCACATAATGGTTTGT
miR-222		AGCTACATCTGGCTACTGGGTCTC
miR-218b		TTGTGCTTGATCTAACCATGTG
novel_89		TCCCACATGGTCTAGCGG
novel_200		GGAGACAGGTGCTAAGAGCTCT
novel_176		TGAGGGGCAGAGAGCGAGAC
miR-107		AGCAGCATTGTACAGGGCTATCA
miR-98		TGAGGTAGTAAGTTGTATTGTT
miR-196b-5p		TAGGTAGTTTCCTGTTGTTGGG
miR-23b		ATCACATTGCCAGGGATTACCA
miR-151-5p		TCGAGGAGCTCACAGTCTAGT
miR-22-3p		AAGCTGCCAGTTGAAGAACTGT
miR-133a-3p		TTGGTCCCCTTCAACCAGCTG
miR-17-3p		ACTGCAGTGAAGGCACTTGTAG
miR-96-5p		TTTGGCACTAGCACATTTTTGCT
miR-140-5p		AGTGGTTTTACCCTATGGTAG
miR-148a-3p		TCAGTGCACTACAGAACTTTGT
miR-181a		AACATTCAACGCTGTCGGTGAGTT
miR-455-3p		GCAGTCCATGGGCATATAC
miR-497		CAGCAGCACACTGTGGTTTGT
**inhibitors**		
inhibitor-miR-190a		CCTAATATATCAAACATATCA
**siRNAs**		
Hspa5	Sense	GCGUCGGUGUGUUCAAGAATT
	Antisense	UUCUUGAACACACCGACGCTT
Hsp90b1	Sense	GAUGCUUUAGACAAGAUAATT
	Antisense	UUAUCUUGUCUAAAGCAUCTT
ANXA2	Sense	GAGUGUACAAGGAAAUGUATT
	Antisense	UACAUUUCCUUGUACACUCTT
Tuba1b	Sense	GGACAAGCUGCUGGAGAACTT
	Antisense	GUUCUCCAGCAGCUUGUCCTT
Sfn	Sense	GCAUCGAGGUGAUACAGAATT
	Antisense	UUCUGUAUCCACCUCGAUGCTT

Table S2 Primer sequences used for RT-qPCR

Gene	Primer	Sequence (5′-3′)
PEDV	Forward	CACCTCCTGCTTCACGTACA
	Reverse	AGCTCCACGACCCTGGTTAT
Rabggta	Forward	TGCGTGTGAACCCTAAGTCC
	Reverse	CTGTGCAGCTACAAATCGCC
FCAR	Forward	CCTGAATGAGCTCGTGTCCCT
	Reverse	CCTCATTTCCATGCAGCCATCG
STK35	Forward	CAGCTACGGCGTGGTTTATG
	Reverse	TAGTGCCAACTCCACGTTCTC
ACTN4	Forward	ATCACTGCTGAGGAACTGCG
	Reverse	AGGACTTGTAGTCAAGGGCG
SDC3	Forward	GTGCTCGTAGCCGTGATCGT
	Reverse	CTTCCAAGGTGTAGCTGCCTT
RCSD1	Forward	TACAGCCCCGAAAGTGACC
	Reverse	GCTTCTCGATCAGGGGTGAG
TMOD3	Forward	TGAGCCACCAAATCCAACCA
	Reverse	TGTTTCACATGCGTGTTGGC
RAB22A	Forward	CGGTTTGTGGAAGACAGTTTTGA
	Reverse	TGTAGCTCATTTTGGTACTGGACA
NEDD9	Forward	GGAGTTTGAGCGACAGCAGA
	Reverse	TGAAATGGGTCTCGCACTGG
LMTK2	Forward	GTTTGACCAGGAAACCCCCA
	Reverse	CGGAATTCATGCACCTCCCT
KIF1B	Forward	GGCAAAGTCATTTCGGCCTT
	Reverse	CCTCCTTCAACTCCCGAACC
GAN	Forward	GTGGATAGCACACGATGTAGAGA
	Reverse	CTGCACTCTTTGACGATTTCTCG
ANXA2	Forward	GTCCAGTGCATCCAGAACAAG
	Reverse	TCACTGCGAGAGACCATGA
LncRNA TS2083	Forward	AGGCTGGAGTACAGTGGCTATTC
	Reverse	GTTCGGCATCAATATGGTGACCTC
IL-1β	Forward	TCTGCATGAGCTTTGTGCAAAG
	Reverse	ACAGGGCAGACTCGAATTCAAC
IL-6	Forward	CCACTTCACAAGTCGGAGGCTT
	Reverse	CCAGCTTATCTGTTAGGAGA
TNF-α	Forward	CCTATGTCTCACGCCCTTCT
	Reverse	CCTGGTATGAGATAGCAAAT
GAPDH	Forward	ATGGTGAAGGTCGGTGTGAA
	Reverse	TGGAAGATGGTGATGGGCTT
**miRNA (Poly-A Tail)**		
miR-15a		GCGCTAGCAGCACATAATGGTTTGT
miR-107		AGCAGCATTGTACAGGGCTATCA
miR-98		GCCGTGAGGTAGTAAGTTGTATTGTT

miR-196b-5p	GGCTAGGTAGTTTCCTGTTGTTGGG
miR-23b	ATCACATTGCCAGGGATTACCA
miR-222	AGCTACATCTGGCTACTGGGTCTC
miR-151-5p	TCGAGGAGCTCACAGTCTAGT
miR-22-3p	AAGCTGCCAGTTGAAGAACTGT
miR-190a	GCCGGGCCTGATATGTTTGATA
miR-497	CAGCAGCACACTGTGGTTTGT
miR-140-5p	GCCAGTGGTTTTACCCTATGGTAG
miR-181a	AACATTCAACGCTGTCGGTGAGTT
miR-148a-3p	TCAGTGCACTACAGAACTTTGT
miR-96-5p	GGTTTGGCACTAGCACATTTTTGCT
miR-455-3p	GCAGTCCATGGGCATATACAC
miR-17-3p	ACTGCAGTGAAGGCACTTGTA
miR-133a-3p	TTGGTCCCCTTCAACCAGCTG
miR-218b	GCGCTTGTGCTTGATCTAACCATGTG
novel_89	TCCCACATGGTCTAGCGG
novel_176	TATGAGGGGCAGAGAGCGAGA
novel_200	GGAGACAGGTGCTAAGAGCTCT
novel_199	CTTGGCACCTAGTAAGCACTCA
cel-miR-39-3p	TCACCGGGTGTAAATCAGCTTG

Table S3 Pairwise protein sequence identity and similarity analysis of TMOD3 and ANXA2

Protein	Reference species	Reference accession no.	Compared species	Compared accession no.	Alignment coverage (%)	Identity	Similarity
TMOD3	Pig (*Sus scrofa*)	Q9N0Y9 / NP_999459	Human (*Homo sapiens*)	Q9NYL9	100	333/352 (94.6%)	341/352 (96.9%)
TMOD3	Pig (*Sus scrofa*)	Q9N0Y9 / NP_999459	Mouse (*Mus musculus*)	Q9JHJ0	100	315/352 (89.5%)	332/352 (94.3%)
ANXA2	Pig (*Sus scrofa*)	P19620	Human (*Homo sapiens*)	P07355	100	332/339 (97.9%)	334/339 (98.5%)
ANXA2	Pig (*Sus scrofa*)	P19620	Mouse (*Mus musculus*)	P07356	100	331/339 (97.6%)	333/339 (98.2%)

Pig protein sequences were used as the reference sequences for pairwise comparison. Identity and similarity values are shown as aligned residues/total aligned residues, with percentages in parentheses.
