# Supplementary material for: Small extracellular vesicle-mediated epithelial–macrophage crosstalk contributes to macrophage dysfunction during PEDV infection
Source: Nat Commun. 2026 Sep 18;17:11548. doi: 10.1038/s41467-026-77854-w (PMC13748656; doi:10.1038/s41467-026-77854-w)
Supplement: Supplementary file 2 [file 41467_2026_77854_MOESM2_ESM.pdf]

Description of Additional Supplementary Files

Title: Supplementary Movie 1

Description: Representative time-lapse imaging of macrophage migration after treatment with miR-NC. Images were acquired at 30-s intervals for 30 min, with LPS (1 μg/mL) added at the beginning of imaging to enhance random cell migration.

Title: Supplementary Movie 2

Description: Representative time-lapse imaging of macrophage migration following treatment with miR-190a. Imaging was performed at 30-s intervals for 30 min, and LPS (1 μg/mL) was added at the start to enhance random migration.
