# Supplementary material for: Small extracellular vesicle-mediated epithelial–macrophage crosstalk contributes to macrophage dysfunction during PEDV infection
Source: Nat Commun. 2026 Sep 18;17:11548. doi: 10.1038/s41467-026-77854-w (PMC13748656; doi:10.1038/s41467-026-77854-w)
Supplement: Supplementary file 3 [file 41467_2026_77854_MOESM5_ESM.pdf]

nature portfolio

Corresponding author(s): Yuchen Li

Last updated by author(s): Aug 10, 2026

Reporting Summary

Nature Portfolio wishes to improve the reproducibility of the work that we publish. This form provides structure for consistency and transparency in reporting. For further information on Nature Portfolio policies, see our Editorial Policies and the Editorial Policy Checklist.

Statistics

For all statistical analyses, confirm that the following items are present in the figure legend, table legend, main text, or Methods section.

n/a	Confirmed	
☐	☒	The exact sample size (*n*) for each experimental group/condition, given as a discrete number and unit of measurement
☐	☒	A statement on whether measurements were taken from distinct samples or whether the same sample was measured repeatedly
☐	☒	The statistical test(s) used AND whether they are one- or two-sided<br>*Only common tests should be described solely by name; describe more complex techniques in the Methods section.*
☒	☐	A description of all covariates tested
☒	☐	A description of any assumptions or corrections, such as tests of normality and adjustment for multiple comparisons
☐	☒	A full description of the statistical parameters including central tendency (e.g. means) or other basic estimates (e.g. regression coefficient) AND variation (e.g. standard deviation) or associated estimates of uncertainty (e.g. confidence intervals)
☐	☒	For null hypothesis testing, the test statistic (e.g. *F*, *t*, *r*) with confidence intervals, effect sizes, degrees of freedom and *P* value noted<br>*Give P values as exact values whenever suitable.*
☒	☐	For Bayesian analysis, information on the choice of priors and Markov chain Monte Carlo settings
☒	☐	For hierarchical and complex designs, identification of the appropriate level for tests and full reporting of outcomes
☒	☐	Estimates of effect sizes (e.g. Cohen's *d*, Pearson's *r*), indicating how they were calculated

Our web collection on statistics for biologists contains articles on many of the points above.

Software and code

Policy information about availability of computer code

Data collection	Confocal images were acquired using ZEN 2.3 lite for the LSM 980 system and [Nikon acquisition software and version] for the Nikon A1 system. Flow-cytometry data were acquired using CytExpert 2.6.0.105. Nanoparticle-tracking data were collected and processed using ZetaView 8.05.14 SP7.
Data analysis	Statistical analyses and graph generation were performed using SPSS v17.0 and GraphPad Prism v9.0.0. Western-blot densitometry and cell-migration trajectory analyses were performed using ImageJ v1.8.0. Fluorescence images were analyzed using ZEN v2.3 lite. Flow-cytometry data were analyzed using FlowJo [version] and CytExpert v2.6.0.105. Omics analyses were performed using the software and versions specified in the Methods

For manuscripts utilizing custom algorithms or software that are central to the research but not yet described in published literature, software must be made available to editors and reviewers. We strongly encourage code deposition in a community repository (e.g. GitHub). See the Nature Portfolio guidelines for submitting code & software for further information.

Data

Policy information about availability of data

All manuscripts must include a data availability statement. This statement should provide the following information, where applicable:

- Accession codes, unique identifiers, or web links for publicly available datasets
- A description of any restrictions on data availability
- For clinical datasets or third party data, please ensure that the statement adheres to our policy

The scRNA-seq data from the intestinal mucosa of both healthy and PEDV-infected piglets have been deposited in the NCBI Gene Expression Omnibus (GEO) under the accession number GSE306895 (https://ncbi.nlm.nih.gov/geo/query/acc.cgi?acc=GSE306895). The whole-transcriptome sequencing data of sEVs derived from intestinal epithelial cells of the control, WIV PEDV-treated, and PEDV-infected groups, together with the RNA-seq data of macrophages treated with these sEVs, are available in GEO under accession numbers GSE305997 (https://ncbi.nlm.nih.gov/geo/query/acc.cgi?acc=GSE305997) and GSE305999 (https://ncbi.nlm.nih.gov/geo/query/acc.cgi?acc=GSE305999). Furthermore, the transcriptome sequencing data for macrophages treated with lncRNA TS2083 have also been uploaded to the Sequence Read Archive (SRA) under accession PRJNA1309073(https://dataview.ncbi.nlm.nih.gov/object/PRJNA1309073?reviewer=qr0gnvnkuf7gdks0naoi3bq9cc). The mass spectrometry proteomics data of the interactome of lncRNA TS2083 have been deposited to the ProteomeXchange Consortium via the iProX partner repository with the dataset identifier PXD069460 (https://proteomecentral.proteomexchange.org/cgi/GetDataset?ID=PXD069460). Source Data are provided with this paper. All data supporting the findings of this study are available within the article, the Supplementary Information, the Source Data file, and the public repositories listed above. Author-generated scripts used for omics-related analyses are publicly available at https://github.com/apanhui/scRNA_hsa. The repository includes a README file describing the analytical workflow and execution instructions. Other omics analyses were performed using standard software and are described in the Methods.

Research involving human participants, their data, or biological material

Policy information about studies with human participants or human data. See also policy information about sex, gender (identity/presentation), and sexual orientation and race, ethnicity and racism.

Reporting on sex and gender	Not applicable in this study.
Reporting on race, ethnicity, or other socially relevant groupings	Not applicable in this study.
Population characteristics	Not applicable in this study.
Recruitment	Not applicable in this study.
Ethics oversight	Not applicable in this study.

Note that full information on the approval of the study protocol must also be provided in the manuscript.

Field-specific reporting

Please select the one below that is the best fit for your research. If you are not sure, read the appropriate sections before making your selection.

☒ Life sciences ☐ Behavioural & social sciences ☐ Ecological, evolutionary & environmental sciences

For a reference copy of the document with all sections, see nature.com/documents/nr-reporting-summary-flat.pdf

Life sciences study design

All studies must disclose on these points even when the disclosure is negative.

Sample size	Because this was an exploratory study with a lack of preliminary data for power analysis, the sample size was determined primarily based on resource limitations. We maximized the number of animals within the constraints of available resources (time, funding, and animal ethics guidelines), ultimately setting the sample size at ≥3 animals per group.
Data exclusions	There are no data excluded from analyses.
Replication	All experiments had at least 3 independent biological replicates. All attempts at replication were successful.
Randomization	Piglets were randomly assigned to experimental groups.
Blinding	Investigators responsible for data collection and quantitative analysis were blinded to group allocation. Group identities were revealed only after completion of the analyses.

Reporting for specific materials, systems and methods

We require information from authors about some types of materials, experimental systems and methods used in many studies. Here, indicate whether each material, system or method listed is relevant to your study. If you are not sure if a list item applies to your research, read the appropriate section before selecting a response.

Materials & experimental systems

n/a	Involved in the study
☐	☒ Antibodies
☐	☒ Eukaryotic cell lines
☒	☐ Palaeontology and archaeology
☐	☒ Animals and other organisms
☒	☐ Clinical data
☒	☐ Dual use research of concern
☒	☐ Plants

Methods

n/a	Involved in the study
☒	☐ ChIP-seq
☐	☒ Flow cytometry
☒	☐ MRI-based neuroimaging

Antibodies

Antibodies used

Rabbit anti-CD63 monoclonal antibody(Abcam, UK, ab134045, 1: 1000) ;
Rabbit anti-CD81 monoclonal antibody (Abcam, UK, ab109201, 1: 1000);
Rabbit anti-HSP70 polyclonal antibody (Abcam, UK, ab181606, 1: 1000) ;
Rabbit anti-Calnexin monoclonal antibody (Abcam,UK, ab133615, 1: 1000) ;
Rabbit anti-TMOD3 polyclonal antibody (Proteintech, China, 12521-1-AP, 1: 200);
Mouse anti-ANXA2 monoclonal antibody (Proteintech, China, 66035-1-Ig, 1: 200);
Rabbit horseradish peroxidase (HRP)-conjugated secondary antibodies (Cell Signaling Technology, USA,7074, 1:5000 dilution) ;
Mouse Horseradish peroxidase (HRP)-conjugated secondary antibodies (YEASEN , China, USA, 771499ES50,1:5000 dilution) ;
Goat Anti-Rabbit IgG H&L (Alexa Fluor ® 594) antibody (YEASEN , China, 771384ES60; 1:200);
Goat Anti-Mouse IgG H&L (Alexa Fluor ® 594) antibody (YEASEN , China, 33212ES60; 1:200);
Mouse anti-PEDV N protein monoclonal antibody was custom-made by Jinsirui Biotechnology (Nanjing, China) and preserved in our laboratory (1:200 dilution).

Validation

-Rabbit Anti-CD63 monoclonal antibody(Abcam, UK, ab134045, 1: 1000) : Verified reactivity against human; validated for Western Blot, IHC-P.
https://www.abcam.cn/products/primary-antibodies/cd63-antibody-epr5702-late-endosome-marker-ab134045
-Rabbit anti-CD81 monoclonal antibody (Abcam, UK, ab109201, 1: 1000) : Verified reactivity against human, mouse, rat; validated for Western Blot.
https://www.abcam.cn/products/primary-antibodies/cd81-antibody-epr4244-ab109201
-Rabbit anti-HSP70 polyclonal antibody (Abcam, UK, ab181606, 1: 1000) : Verified reactivity against human, mouse, rat; validated for Western Blot, Flow Cytometry (Intra), Flow Cytometry, IHC-P, ICC/IF.
https://www.abcam.cn/products/primary-antibodies/hsp70-antibody-epr16892-ab181606
-Rabbit anti-Calnexin monoclonal antibody (Abcam,UK, ab133615, 1: 1000):Verified reactivity against human;validated for IHC-P, Flow Cyt (Intra), Western Blot.
https://www.abcam.com/en-us/products/primary-antibodies/calnexin-antibody-epr36332-er-membrane-marker-ab133615
-Rabbit anti-TMOD3 monoclonal antibody (Proteintech, China, 12521-1-AP, 1: 200): Verified reactivity against human, mouse, rat; validated for WB, IHC, IP, ELISA.
https://www.ptgcn.com/products/TMOD3-Antibody-12521-1-AP.htm
-Mouse anti-ANXA2 monoclonal antibody (Proteintech, China, 66035-1-Ig, 1: 200) : Verified reactivity against human, mouse, rat; validated for WB, IHC, IF/ICC, FC (Intra), ELISA and More .
https://www.ptgcn.com/products/ANXA2-Antibody-66035-1-Ig.htm
-Rabbit Horseradish peroxidase (HRP)-conjugated secondary antibodies (Cell Signaling Technology, USA, 7074, 1:5000 dilution) : Verified reactivity against Rabbit; validated for WB.
https://www.cellsignal.cn/products/secondary-antibodies/anti-rabbit-igg-hrp-linked-antibody/7074
-Mouse Horseradish peroxidase (HRP)-conjugated secondary antibodies (YEASEN , China, USA, 771499ES50,1:5000 dilution) : Verified reactivity against Mouse; validated for ELISA,IP,WB.
https://www.yeasen.com/products/detail/1951306980551299073
-Goat Anti-Rabbit IgG H&L (Alexa Fluor ® 594) antibody (YEASEN , China, 771384ES60; 1:200): Verified reactivity against Rabbit; validated for IHC, IF/ICC, FC .
https://www.yeasen.com/products/detail/1951306972972191746
-Goat Anti-Mouse IgG H&L (Alexa Fluor ® 594) antibody (YEASEN , China, 33212ES60; 1:200);Verified reactivity against mouse IgG; validated for IHC, IF/ICC, FC .
https://www.yeasen.com/products/detail/462
-Mouse anti-PEDV N protein monoclonal antibody was custom-made by Jinsirui Biotechnology (Nanjing, China) and preserved in our laboratory (1:200 dilution).

Eukaryotic cell lines

Policy information about cell lines and Sex and Gender in Research

Cell line source(s)	The 3D4/21 porcine alveolar macrophage cell line (ATCC CRL-2843) and IPEC-DQ cells were maintained in our laboratory. IPEC-DQ is a PEDV-susceptible subclone derived from IPEC-J2 cells by limiting dilution. The RAW 264.7 (ATCC TIB-71), HEK293T cells (ATCC CRL-3216) and THP-1 cells (ATCC TIB-202) were obtained from the American Type Culture Collection.
Authentication	IPEC-DQ cells were verified by their expected morphology, EpCAM and villin expression, and susceptibility to PEDV infection. The 3D4/21 cell line was not available and therefore was not used in this study. All other cell lines were validated and authenticated.
Mycoplasma contamination	All cell lines tested negative for mycoplasma contamination.
Commonly misidentified lines (See ICLAC register)	Not applicable in this study.

Animals and other research organisms

Policy information about studies involving animals; ARRIVE guidelines recommended for reporting animal research, and Sex and Gender in Research

Laboratory animals	All piglets used in this study were female three-way crossbred pigs obtained from a commercial farm in Gaochun, Jiangsu Province, China. Three-day-old neonatal piglets were used for PEDV challenge experiments, two-week-old piglets were used as donors for primary alveolar macrophage isolation, and ten-day-old piglets were used for intestinal ligated-loop and serum-sEV experiments. All animals tested negative for PEDV, TGEV, PRRSV, PCV2 and PRCV. After arrival, piglets were randomly assigned to experimental groups, housed by group in designated pens, and allowed to acclimatize for 24 h before experimentation.
Wild animals	The study did not involve wild animals.
Reporting on sex	All piglets used in this study were female; therefore, sex was not evaluated as an experimental variable and no sex-based analysis was performed.
Field-collected samples	The study did not involve samples collected from the field.
Ethics oversight	All animal procedures were conducted in accordance with institutional guidelines and were approved by the Animal Experiment Ethics Committee of Nanjing Agricultural University (protocol no. NJAULLSC2023089)

Note that full information on the approval of the study protocol must also be provided in the manuscript.

Plants

Seed stocks	Not applicable in this study.
Novel plant genotypes	Not applicable in this study.
Authentication	Not applicable in this study.

Flow Cytometry

Plots

Confirm that:

☒ The axis labels state the marker and fluorochrome used (e.g. CD4-FITC).

☒ The axis scales are clearly visible. Include numbers along axes only for bottom left plot of group (a 'group' is an analysis of identical markers).

☒ All plots are contour plots with outliers or pseudocolor plots.

☒ A numerical value for number of cells or percentage (with statistics) is provided.

Methodology

Sample preparation	Macrophages were collected, washed with PBS, and analyzed by flow cytometry under identical acquisition settings. For

Sample preparation	phagocytosis analysis, macrophages were incubated with APC-labeled fluorescent beads, washed to remove non-associated beads, and detected by flow cytometry. For F-actin analysis, cells were fixed, permeabilized, stained with FITC-conjugated phalloidin, and analyzed by flow cytometry. The results were processed using FlowJo software.
Instrument	CytoFLEX S
Software	CytExpert Setup-2.6.0.105
Cell population abundance	Migrated macrophages were quantified by fixed-volume acquisition. APC-positive macrophage populations indicated fluorescent bead uptake, and the percentage of APC-positive cells and APC fluorescence intensity were quantified. FITC fluorescence intensity of phalloidin-stained macrophages was used to evaluate cellular F-actin levels.
Gating strategy	Cell populations were initially identified based on FSC and SSC profiles. Single-cell populations were subsequently selected for analysis. APC-positive and FITC-positive populations were identified for quantification of bead uptake and F-actin expression, respectively. The same gating strategy was applied to all experimental groups.

☒ Tick this box to confirm that a figure exemplifying the gating strategy is provided in the Supplementary Information.
