# Supplementary material for: Small extracellular vesicle-mediated epithelial–macrophage crosstalk contributes to macrophage dysfunction during PEDV infection
Source: Nat Commun. 2026 Sep 18;17:11548. doi: 10.1038/s41467-026-77854-w (PMC13748656; doi:10.1038/s41467-026-77854-w)
Supplement: Supplementary file 4 [file 41467_2026_77854_MOESM6_ESM.pdf]

nature portfolio

Peer Review File

Small extracellular vesicle-mediated epithelial–macrophage crosstalk contributes to macrophage dysfunction during PEDV infection

Corresponding Author: Dr Yuchen Li

This file contains all reviewer reports in order by version, followed by all author rebuttals in order by version.

Version 0:

Reviewer comments:

Reviewer #1

(Remarks to the Author)
This manuscript presents a comprehensive investigation integrating in vivo piglet infection, single-cell RNA-seq, epithelial–macrophage co-culture models, extracellular vesicle profiling, and ncRNA-mediated functional assays to characterize how PEDV induces mucosal and systemic immunosuppression. The study addressed an important question in enteric coronavirus pathogenesis and highlights a potentially novel exosome-mediated ncRNA mechanism. Although the dataset is extensive and the observations are compelling, several conceptual and mechanistic issues limit the strength of the conclusion that exosomal miR-190a and LncRNA TS2083 together constitute a unified and necessary mechanism of PEDV immune evasion.

Major comments
The authors proposed a mechanistic model in which PEDV-induced epithelial exosomes deliver miR-190a and LncRNA TS2083 to macrophages, thereby suppressing migration and phagocytosis through cytoskeletal regulation. While the authors presented strong in vitro data supporting the regulatory roles of these ncRNAs, the study does not directly demonstrate that they are essential mediators of macrophage dysfunction during in vivo PEDV infection. Exosomes contain a wide range of RNAs and proteins, and PEDV infection induces broad changes in epithelial signaling; therefore, the specific contributions of miR-190a and TS2083 cannot be established solely on the basis of cell-based perturbations. As a result, the proposed ncRNA–cytoskeleton axis appears more suggestive than definitive. The overall conclusion would be more balanced if supported by in vivo perturbation of these molecules or if framed as identifying likely contributors to PEDV-induced immunosuppression rather than definitive central determinants.

Minor comments
The authors used two different identifiers for the long noncoding RNA: LncRNA TS2083 and TS2038.

Reviewer #2

(Remarks to the Author)
This manuscript introducing an interesting discovery on PEDV-induced exosomes to suppress alveolar macrophages, thereby extending mucosal immunosuppression. The discovery could be essential to understand the coronavirus immune evasion at the molecular level and find the effective therapeutic targets. Overall, the work is original. However, more concerns are listed below to address before it can be further considered for publishing.

1. Following the ISEV guideline, the term of "exosome" needs to be specifically defined than using the term of "extracellular vesicles". In Figure 2B, the marker expression level was increased from PEDV-Exo than Mock Exo. It is unclear about the data explanation. Since the particle number from PEDV-Exo was increased, which could be due to the increased particle number, not marker expression level itself. Additionally, validation of purity and specificity of exosomes need to have more data evidence and characterization.

2.The statistical data analysis needs to be improved. For imaging analysis, the statistical method should be clearly described. If only 3 regions of sample or 3 samples were analyzed, it is not rigorous enough. It should clearly describe the

method used for quantifying the images.

3. Figure 3G, the image was not focused, and resolution quality needs to be improved.

4. it is not clear how the number of migrated cells was counted to generate the quantitative data.

5, it is unclear how the LncRNAs are more important in this manuscript, other type of RNAs should be investigated in the manuscript.

6. Although this manuscript was trying to prove the role of exosomes in the cellular functional assay, the secretome other than exosomes should be included as the control group in figure 1 H-L, Figure 2, Figure 3, and Figure 5.

Reviewer #4

(Remarks to the Author)
It is quite innovative that this study investigated exosome-mediated immune evasion mechanisms of porcine epidemic diarrhea virus (PEDV). Overall, the studies were designed well and comprehensive, and the paper was written well. Because only PEDV was studied, the title should be revised to be specific: change “enteric coronavirus” to “PEDV”. Otherwise, the authors need to provide data for at least one more enteric coronavirus. Also, age-dependent disease susceptibility is a characteristic for PEDV. It would be nice to investigate whether such exosomes are generated in adult pigs, which usually show mild clinical signs and no mortality after PEDV infection.
Specific comments:
1. Please spell out all abbreviations when a word first appears in the manuscript.
2. Fig. 6H. It seemed that miR-190a not only reduced TMOD3 abundance and disrupted its co-localization with actin fibers but also induced the aggregation of TMOD3. Please describe and explain it.
3. Please provide the ATCC# or the original resource of your IPEC-DQ cells.
4. P503. “virulent isolates (GenBank: JX002693)” just provides the M gene sequence of the PEDV Zhejiang08 strain. Is there complete genomic sequence available for this strain?
5. P527-528, Pigs “were housed individually for 24 h prior to experimentation to allow acclimatization and minimize stress.” Did it increase stress since pigs are social animals?

Reviewer #5

(Remarks to the Author)
The work “Exosome-mediated macrophage reprogramming drives mucosal and systemic immunosuppression in enteric coronavirus infection” is about the entry of the enteric alphacoronavirus porcine epidemic diarrhoea virus (PEDV) into epithelial cells and their subsequent production of extracellular vesicles (EVs). These EVs have a particular miRNA/lncRNA consistency, and the authors describe their effect on macrophage function. They suggest that this interaction suppresses pathways that cause the development of the disease, despite upregulation of IFN pathways. The work is technologically extensive, with a lot of data, and has a strong rhetorical foundation for investigation. However, I cannot approve this work for publication without the following revisions to the manuscript.

There are some questions on the translatability of this finding towards other species, like humans or mice. Can the authors show any translatability from these models? How much homogeneity of these targeted genes is there compared to other species? Even if it is only in vitro, showcasing that these types of dynamics can also play a role in human-related infections would strengthen the work immensely. This work is strong for the observations that the authors have obtained, but this is a clear part of the work that is lacking. Likewise, the authors introduce the economic burden of viruses like SARS-CoV-2 and PEDV, but despite all the knowledge acquired during this publication, there is no experiment that progresses to halting the disastrous effects of PEDV. However, since I am no expert in sus scrofa models, and the authors do show relations in vivo, this might be outside the possibilities of the model.
Major/minor revision:
1. The authors stated that they performed scRNA-seq: “Jejunal mucosa (pooled from two piglets) was collected for single-cell RNA sequencing (scRNA-seq)”. And performed subsequent analysis, even adding additional datasets: see “Methods - Processing the single cell RNA sequencing data”
a. I have issues with the experimental design of the generated dataset in this study. We see that samples were pooled prior to generating the data, which causes a loss of replication, meaning that the dataset is now an n=1 per condition – because we are unable to understand the balance between the two replicates and, therefore, the balance of any observation. This does not allow for any statistical analysis at the experimental level.
b. The differential abundance analysis of cell types (S1B/C) cannot be performed by an n=1/2.
c. I also have an issue with the inflation of observation that ensues in the analysis afterwards (GSEA for GO-terms or KEGG). Aside from the fact that there is no code supplied to go through the details of the analysis, the authors used cells as independent observations in the dataset. This leads to inflated significance, even when correcting for multiple testing – a common mistake with scRNA-seq or other single-cell experiments (PMC6785265).
A whole-tissue response from the treatment of the virus per piglet would create dependencies of responses between cells in the tissue.
i. To determine the correct response from the piglets, this experiment should be repeated with the appropriate number of repeats to perform statistical analysis. If this is not possible, the analysis should be rewritten to a validation of the results, with clear explanations about the limitations of the analysis and that the results should be interpreted with caution.

Alternatively, if the public dataset does have the required amount of experimental observations to perform a correct analysis, start with this and use the experiment as a replication.
ii. Fig. S1I–K graphs do not correspond with the graphs in the main text, making it difficult to compare them. Please pick one visualisation and use them for both (for instance in CellChat). Also please highlight the overlapping pathways in another colour so that the reader does not have to look for the overlap him/her self.
2. The authors go on to describe an impaired state of function in the macrophage, based on the analysis of scRNA-seq they derive a loss of migration and phagocytosis which is tested in vitro using microscopy. They attribute these effects to signalling derived from epithelial cells after incubation with PEDV
a. The authors take along a mock virus particle in their experimental design, which seems like an appropriate control.
i. To assure that this control is indeed correct, immune activation markers of inflammation from macrophages should be compared. I understand that the whole point is that the cargo of the virus differs and creates a different type of response, however, since inflammatory immune activation in macrophages change's functon(e.g. reducing phagocytic capacity via PGE2 and EP4 PMID: 40383415 , which are also involved in migration) it would be good to show that this effect stems from an effect beyond inflammatory activation of the macrophage.
b. Although the images look convincing for migration it would strengthen the story to repeat this work by other techniques that are less prone to technological bias, but rather give an overview of the whole sample.
i. I suggest detaching the cells after migration and counting them through a flow cytometry experiment.
c. Likewise, the phagocytosis of fluorescent particles is measured with counting beads in microscopy
i. Also here, I would suggest using flow cytometry to generate an overview of the whole sample to strengthen the observation.
ii. Minor note on microscopy images, please use magenta and green in all future displays of immunofluorescence images, as this is a colourblind friendly palette.
d. The two comments above (point 2b and 2c) also apply to all other experiments of a similar set up.
3. Next, after testing the PEDV indirectly on macrophages, the authors make an interesting conclusion: PEDV increases extracellular vesicle (EVs, or exosomes) production. Although the direct rational for this direction is lacking (maybe they read it somewhere? – would be nice to find a reference or the observation which led them to this direction for readability), the authors perform an impressive characterisation in Figure 2.
a. For 2B, it would be informative to add a control (for instance a housekeeping gene from the cells that produced the material) to show that the source of production is at the same level as MOCK.
b. 2C. It is unclear how the increase of particles is measured. It would make sense to perform an area under the curve analysis to show repeated experiments in a bar plot.
4. The authors go on to investigate the effects of these EVs on macrophages. They demonstrate uptake of EVs and show that there are transcriptional and functional changes upon treatment with these EVs.
a. The authors state that there is a difference between PEDV and WIV control in terms of transcriptional activation:
i. How did the authors quantify (and correct for) the EV-load per sample? If PEDV makes more EVs, then a higher response is to be expected. Is it load or a more potent effect due to composition? Also, considering their GW4869 control experiment.
1. Please put forward a clear hypothesis with regards to this in the text, so that the reader gets a grasp of what is the goal of the experiments. If the hypothesis is composition, consider switching first displaying differences in composition and then effects of function – for the sake of flow.
b. Can the authors consider other ways for visualisation of the effect size difference between induction of transcriptional pathway effects between PEDV and WIV control? This is difficult to read, especially because the comparison is between figures at two different locations.
5. Afters identifying these effects, the authors do experiments to understand the composition of the EVs that affect the macrophages. The authors found differences between active and inactive virus and start to screen a selection of the differentially expressed miRNAs finding miR-190a as a repressor of migration.
a. It is unclear what type of analysis was performed to determine the up or downregulation of miRNA, nor what was the rational for selection of migration. Did you use the pathway analysis for this? Then please make this clearer that this was the foundation for selection of the panel in the text.
b. Please highlight miR-190a in figure 4A, so that the reader can find it easier there to see if this migration effect is in accordance with differential expression, but does not seem to play a role in phagocytosis.
c. In figure 4B, please show individual experiments as dots, it is now not possible to estimate any other effects
i. For instance, miR-140b-5p effect size and errorbar look significantly higher than the NC and is also upregulated in PEDV. But there is no way to determine the consistency of this observation right now.
6. For the AFM part I cannot give any comments, as this is outside my area of expertise. Most noticeable is the fact that the experimental design changes suddenly to an LPS-induced model, to show inhibition of LPS effect, however, previously this was not the model, nor is there any comparison on a functional level, like TNFa production to gauge how broad the effect of miR-190a is in repressing inflammation.
7. Next the authors continue to investigate the effect of actin dynamics by miR-190a, they obtain beautiful images on disrupted dynamics and obtain mechanistic insight into how miR-190a affects the cell.
a. Can figure 6A be repeated with flow cytometry?
b. Please put a white arrow what is the focus point of your image in 6B for the control.
c. It is interesting to see that although the miRNA has a strong effect, the actual effect on the gene of interest in rather low, not even a 50% decrease. Can the authors speculate on this?

Reviewer #6

(Remarks to the Author)
The editor specifically requested that my review focus on the AFM data. Given my current workload, I have only been able to briefly skim the rest of the manuscript and have therefore restricted my comments to the AFM and nanoindentation experiments.

The study aims to demonstrate that exosomal miR-190a suppresses macrophage migration by modulating cellular mechanics, and the authors use both Chiaro nanoindentation and AFM-based force spectroscopy to quantify global and local stiffness as well as adhesion forces. While the hypothesis is interesting, the AFM and nanoindentation data, as presented, fall short of current standards for quantitative cell mechanics.

A number of critical methodological details are missing, making it impossible to properly evaluate or reproduce the measurements. For the AFM experiments, the cantilever spring constant, its calibration procedure, the maximum indentation depth, and the model used to extract Young's modulus are not provided. The method for determining the contact point, which is particularly important for soft, adhesive cells, is not described. Additionally, the physiological conditions during measurement, such as temperature, buffering, and cell viability maintenance, are not indicated.

Nanoindentation depths of up to 4 μm raise further concerns, as no correction or discussion of substrate effects is provided. Indentations of this magnitude can be heavily influenced by the stiff underlying substrate, meaning that the reported Young's modulus values may not reflect intrinsic cellular mechanics.

There is also a notable inconsistency between probe geometries and applied forces. Similar force values are reported for both the Chiaro and AFM measurements, despite the use of very different probe radii. This inconsistency complicates direct comparison between techniques and raises questions about the experimental design.
The analysis of AFM force curves also raises concerns. Approach curves are not shown, and the presence of adhesion and hysteresis suggests that a purely elastic Hertz-type model may not be appropriate. Without representative curves and a discussion of the suitability of the chosen mechanical model, it is difficult to assess the validity of the extracted moduli.
Furthermore, the identification of high-stiffness "hotspots" and their interpretation as relevant to front–rear mechanical gradients is speculative, as the spatial resolution, indentation depth, and force setpoints used for mapping are not provided.

The missing experimental details, potential substrate effects, and ambiguities in force curve analysis make it challenging to evaluate the reliability of the reported mechanical changes. Consequently, the conclusions linking miR-190a to modulation of macrophage migration through mechanical effects are not convincingly supported by the current data.

Version 1:

Reviewer comments:

Reviewer #2

(Remarks to the Author)
The second version after revision addressed my major concerns and current version of results are noteworthy and significant to the related field.
The work is original and support the conclusions with enough details and evidence.

Reviewer #4

(Remarks to the Author)
The authors have answered my questions and concerns. The revised manuscript is acceptable.

Reviewer #5

(Remarks to the Author)
The work of the authors of the paper "Small extracellular vesicle-mediated epithelial–macrophage crosstalk contributes to macrophage dysfunction during PEDV infection" has been satisfactory in their rebuttal. All changes, concerning experimental work as well as textual changes, have, in my opinion, significantly strengthened the work and improved its rigour. I only have one final comment to make with regard to the graph visuals in the figure. This will improve interpretability and maximise the authors' opportunity to showcase the scientific rigour and reproducibility they have demonstrated throughout the publication process.
Could the authors please rework all bar graphs in a way that displays the data per experiment? Since the authors do not disclose this information in the text consistently as well (n=?). So basically, all summary statistics bar charts, also those in the supplement, should contain more information. For inspiration, I would like to refer the authors to Figure 1 of the following link. https://rupress.org/jcb/article/219/6/e202001064/151717/SuperPlots-Communicating-reproducibility-and
At the very least, I would appreciate it if the authors showed their mean value per experiment as a dot in all bar graphs. This is an easy conversion in all GraphPad files to do, and will make all data more convincing.
I am also still missing all scripts that have contributed to their findings with regard to any omic analysis. Having this publicly available will help increase rigour and reproducibility for readers. With these changes, I approve this manuscript for publication.

Reviewer #6

(Remarks to the Author)
I appreciate the substantial effort made by the authors to address the methodological concerns raised in the previous review. The revised manuscript now contains additional details regarding probe geometry, fitting procedures, contact-point determination, and the limitations of Hertz-type analysis. The authors have also appropriately toned down several overinterpretations.

However, after examining the revised Figure 5, I remain concerned that the AFM and nanoindentation data do not yet meet current standards for quantitative cell-mechanics measurements.

1. Presentation of the nanoindentation data
The presentation of the force–indentation curves in panels B,E,I, H remains problematic.
The y-axis in panel B is presented in µN which appears unusual.
The contact point appears incorrectly positioned in several representative curves.
The fitting region shown in panel B is not sufficiently justified and the relationship between the selected fitting range and the actual contact point remains unclear.
In panel D, only summary statistics are shown. Given the substantial biological variability expected in live-cell measurements, all individual measurements should be displayed. The panel I is not clear. What is the blue line? Where is the contact point?

2. Quality and interpretability of the representative force curves
The representative curves shown in panels E, H and I exhibit shapes that are difficult to reconcile with standard force-spectroscopy measurements on living cells.
The curves display pronounced hysteresis, irregular transitions, and complex force responses that suggest substantial viscoelasticity, adhesion, or other non-Hertzian behaviour. While such behaviour is not unexpected for living cells, it raises an important issue: it becomes unclear whether these curves are suitable for extracting a meaningful elastic modulus using a Hertz–Sneddon framework.
In particular:
The representative AFM curves shown in panel H do not resemble force curves typically used for reliable modulus extraction.
The manuscript does not provide sufficient evidence that the selected fitting regions correspond to a regime where Hertz-type assumptions are reasonably valid.

3. Contact-point determination remains unclear
Although the authors state that the contact point has been indicated in panel I, the procedure remains insufficiently transparent.
The determination of the contact point is one of the most critical steps in modulus extraction, particularly for soft and highly adhesive biological samples. From the figure, it is not obvious how the contact point was identified, nor how sensitive the resulting modulus values are to this choice.
The figure would benefit from a much clearer illustration of:
the raw force curve,
the baseline,
the chosen contact point,
the fitting interval,
and the resulting model fit.
4. Validity of the modulus values shown in panel J
Most importantly, I remain unconvinced that the curves presented in panels H and I support reliable extraction of an elastic modulus.
The representative curves appear highly dominated by adhesion and hysteretic effects and do not display an obvious region where a simple Hertz–Sneddon model would provide a physically meaningful description of the cell response.
Consequently, the values reported in panel J should be interpreted with extreme caution. Based on the representative curves provided, it is difficult to assess whether the calculated apparent elastic moduli reflect genuine mechanical differences between groups or are instead influenced by fitting choices, contact-point selection, adhesion effects, or other model-dependent artefacts.

5. Cell-height argument
Finally, I remain unconvinced by the statement that the perinuclear cell-body region reaches approximately 16 µm in height. The orthogonal confocal views provided do not clearly support this value, and no quantitative height analysis is presented. Therefore, the possibility of substrate contributions during indentation cannot be fully excluded.

While the authors have substantially improved the methodological description and appropriately moderated the interpretation of the mechanical measurements, the representative force curves themselves remain difficult to interpret and do not convincingly demonstrate that reliable stiffness or elastic modulus values can be extracted. As a result, the mechanical component of Figure 5 remains, in my view, the weakest part of the manuscript and does not yet provide robust

support for the proposed mechanistic conclusions.

Open Access This Peer Review File is licensed under a Creative Commons Attribution 4.0 International License, which permits use, sharing, adaptation, distribution and reproduction in any medium or format, as long as you give appropriate credit to the original author(s) and the source, provide a link to the Creative Commons license, and indicate if changes were made.
In cases where reviewers are anonymous, credit should be given to 'Anonymous Referee' and the source.
The images or other third party material in this Peer Review File are included in the article's Creative Commons license, unless indicated otherwise in a credit line to the material. If material is not included in the article's Creative Commons license and your intended use is not permitted by statutory regulation or exceeds the permitted use, you will need to obtain permission directly from the copyright holder.
To view a copy of this license, visit https://creativecommons.org/licenses/by/4.0/

Reviewer #1 (Remarks to the Author):

This manuscript presents a comprehensive investigation integrating *in vivo* piglet infection, single-cell RNA-seq, epithelial–macrophage co-culture models, extracellular vesicle profiling, and ncRNA-mediated functional assays to characterize how PEDV induces mucosal and systemic immunosuppression. The study addressed an important question in enteric coronavirus pathogenesis and highlights a potentially novel exosome-mediated ncRNA mechanism. Although the dataset is extensive and the observations are compelling, several conceptual and mechanistic issues limit the strength of the conclusion that exosomal miR-190a and lncRNA TS 2083 together constitute a unified and necessary mechanism of PEDV immune evasion.

A: We sincerely thank the reviewer for recognizing the significance, originality, and broad scope of this work, and for providing thoughtful and constructive comments and suggestions, which have greatly helped us improve the manuscript. In response to these comments, we have performed additional experiments, carefully revised the manuscript, and addressed each point in detail below. These revisions and new data have substantially strengthened the study and further improved the clarity and rigor of the manuscript. We hope that the revised version more fully addresses the reviewer's concerns.

Major comments：

The authors proposed a mechanistic model in which PEDV-induced epithelial exosomes deliver miR-190a and lncRNA TS 2083 to macrophages, thereby suppressing migration and phagocytosis through cytoskeletal regulation. While the authors presented strong *in vitro* data supporting the regulatory roles of these ncRNAs, the study does not directly demonstrate that they are essential mediators of macrophage dysfunction during *in vivo* PEDV infection. Exosomes contain a wide range of RNAs and proteins, and PEDV infection induces broad changes in epithelial signaling; therefore, the specific contributions of miR-190a and TS 2083 cannot be established solely on the basis of cell-based perturbations. As a result, the proposed ncRNA–cytoskeleton axis appears more suggestive than definitive. The overall conclusion would be more balanced if supported by *in vivo* perturbation of these molecules or if

framed as identifying likely contributors to PEDV-induced immunosuppression rather than definitive central determinants.

A: We sincerely thank the reviewer for this important and insightful comment. We fully agree that, although our *in vitro* gain- and loss-of-function experiments and rescue assays support the regulatory roles of miR-190a and lncRNA TS2083, and our piglet sEV-administration model and serum-sEV experiments support the macrophage-suppressive activity of PEDV-induced sEVs *in vivo* and *ex vivo*, these data do not by themselves establish sEV-associated miR-190a and lncRNA TS2083 as exclusive or indispensable mediators of macrophage impairment during *in vivo* PEDV infection.

As the reviewer correctly points out, PEDV-induced sEVs contain diverse RNA and protein cargos, and PEDV infection also induces broad changes in epithelial and immune signaling. Therefore, the macrophage-suppressive phenotype observed during PEDV infection is likely to reflect a complex sEV-mediated immunoregulatory program rather than the action of only two ncRNA molecules. We have revised the manuscript accordingly to avoid overstating the causal scope of our findings.

Specifically, we now frame sEV-associated miR-190a and lncRNA TS2083 as functionally important and mechanistically supported contributors to PEDV-induced macrophage dysfunction, rather than as definitive central determinants or the sole necessary mechanism of PEDV immune evasion. In this revised interpretation, miR-190a mainly contributes to impaired macrophage migration through the TMOD3/F-actin/cellular mechanics axis, whereas lncRNA TS2083 contributes to defective phagocytosis through ANXA2 destabilization and impaired phagocytic-cup formation. We have also clarified that the specific *in vivo* contribution of each ncRNA remains to be directly tested.

We agree that *in vivo* perturbation of miR-190a and lncRNA TS2083 during PEDV infection would provide stronger causal evidence. However, specific depletion or blockade of individual sEV-associated ncRNAs in neonatal piglets would require the establishment and validation of a large-animal delivery system with sufficient tissue specificity, stability, biodistribution control, and functional readouts, which is beyond the scope of the current revision. We have now explicitly acknowledged this limitation in the Discussion and stated that future studies using *in vivo* ncRNA blockade, depletion,

or rescue approaches will be necessary to determine the relative contribution of miR-190a and lncRNA TS2083 to macrophage dysfunction during PEDV challenge in neonatal piglets.

In response to the reviewer's suggestion, we have revised the Abstract, Results, Discussion, and schematic model to use more balanced language. For example, we have replaced stronger claims such as "definitive central determinants" or "central mechanism" with more cautious wording such as "important contributors," "likely mediators," or "mechanistically supported components of PEDV-induced sEV-associated macrophage dysfunction." These revisions better align our conclusions with the strength of the experimental evidence (lines 11–13, 15–23, 87-89, 212-213, 243-250, 304-306, 309-322, 364, 377-381, 390-391, 403-406, 431-432, 433, 449, 452-454, 455, 458, 460, 463, 467-469, 501, 503, 509-511, 514-518, 519-521, 528-533 and 1424–1430). We are grateful to the reviewer for helping us define the appropriate scope of the study more precisely.

Minor comments:

The authors used two different identifiers for the long noncoding RNA: LncRNA TS2083 and TS2038.

A: We thank the reviewer for pointing out this inconsistency. After rechecking the transcript annotation, we confirmed that the correct identifier is TCONS_00002083, referred to throughout the manuscript as lncRNA TS2083. The instances of "TS 2038" were typographical errors introduced during manuscript preparation. In the revised manuscript, we have corrected all occurrences of "TS 2038" to "lncRNA TS2083" in the main text, Methods, figure legends, and supplementary materials. This correction only concerns nomenclature and does not affect the experimental results, data analysis, or conclusions.

Reviewer #2 (Remarks to the Author):

This manuscript introducing an interesting discovery on PEDV-induced exosomes to suppress alveolar macrophages, thereby extending mucosal immunosuppression. The discovery could be essential to understand the coronavirus immune evasion at the molecular level and find the effective therapeutic targets. Overall, the work is original. However, more concerns are listed below to address before it can be further considered for publishing.

A: We sincerely thank the reviewer for the positive evaluation of our work and for recognizing the originality and potential significance of this study. We greatly appreciate the reviewer's view that our findings on PEDV-induced epithelial sEVs may provide new insight into coronavirus immune evasion and may be helpful for identifying potential therapeutic targets. We are also grateful for the reviewer's thoughtful and constructive comments. In response, we have carefully revised the manuscript and addressed each of the concerns point-by-point below. We believe that these revisions have substantially improved the rigor, clarity, and overall presentation of the study.

1. Following the ISEV guideline, the term of "exosome" needs to be specifically defined than using the term of "extracellular vesicles". In Figure 2B, the marker expression level was increased from PEDV-Exo than Mock Exo. It is unclear about the data explanation. Since the particle number from PEDV-Exo was increased, which could be due to the increased particle number, not marker expression level itself. Additionally, validation of purity and specificity of exosomes need to have more data evidence and characterization.

A: We sincerely thank the reviewer for this important comment. We agree that the term "exosome" should be used cautiously unless vesicle biogenesis is directly demonstrated. Following the ISEV/MISEV recommendations, we have revised the terminology throughout the manuscript and now refer to the vesicles isolated in this study as small extracellular vesicles (sEVs). We have also added an operational definition of these vesicles based on our isolation method and characterization results, including TEM morphology, NTA size distribution, enrichment of CD63, CD81, and HSP70, and absence of the negative marker Calnexin.

Regarding Fig. 2B, we agree that the stronger CD63, CD81, and HSP70 signals in the PEDV-sEV group may reflect the increased total sEV yield or particle number rather than higher marker abundance per vesicle, because equal volumes of vesicle preparations were loaded in the original experiment. We have revised the text and figure legend to avoid interpreting these data as marker “upregulation.” To clarify this issue, we performed an additional western blot using equal amounts of sEV protein. As shown in the newly added Fig. S5B, CD63, CD81, and HSP70 signals were comparable between MOCK-sEVs and PEDV-sEVs under equal-protein loading conditions, supporting the interpretation that PEDV infection increases sEV production.

We further strengthened the characterization of the sEV-enriched preparations by detecting Calnexin as a negative marker. Calnexin was present in matched whole-cell lysates but was not detected in either MOCK-sEV or PEDV-sEV preparations, supporting enrichment of sEV-associated material and indicating no obvious contamination with Calnexin-positive endoplasmic reticulum-derived cellular components. These revisions and additional data improve the accuracy of our terminology, the interpretation of Fig. 2B, and the characterization of the isolated sEV-enriched preparations.

2. The statistical data analysis needs to be improved. For imaging analysis, the statistical method should be clearly described. If only 3 regions of sample or 3 samples were analyzed, it is not rigorous enough. It should clearly describe the method used for quantifying the images.

A: We sincerely thank the reviewer for this important comment. We agree that the original manuscript did not describe the image-quantification workflow and statistical units with sufficient clarity. In the revised manuscript, we have therefore expanded the Methods section and revised the relevant figure legends to clearly define how imaging data were acquired, quantified, and statistically analyzed.

For tissue imaging analyses, multiple non-consecutive sections were examined from each independent animal/sample, and several randomly selected, non-overlapping fields were imaged from each section. Quantitative values from individual fields were first averaged within each section and then averaged within each animal/sample. The

resulting single mean value for each animal/sample was used as the statistical unit. Thus, microscopic fields and sections were treated as technical subsampling to improve measurement reliability, not as independent biological replicates.

For *in vitro* cell-imaging assays, multiple randomly selected, non-overlapping fields were acquired from each independent experiment or biological replicate. The values from these fields were averaged to generate one value per independent replicate, and statistical comparisons were performed using these biological replicate-level values. We have revised the figure legends to specify the number of sections, fields, cells where applicable, and independent biological replicates used for each imaging-based quantification.

We also clarified the image-analysis methods in the revised Methods section (lines 816–824, 834-839, 1010-1014, 1035-1041 and 1051-1056). Briefly, images were acquired under identical microscope settings within each experiment and quantified using ImageJ/Fiji with consistent thresholding and background-subtraction criteria. Depending on the assay, migrated macrophages, bead uptake per cell, CD68-positive macrophage signals, and *E. coli*–macrophage co-localization were quantified from predefined regions of interest. Statistical analyses were performed using biological replicates as the units of analysis, with unpaired two-tailed Student's t-test or one-way ANOVA as appropriate. These revisions clarify that our statistical analyses were based on independent biological replicates rather than repeated counting of individual fields or sections. We thank the reviewer for this helpful suggestion, which has improved the rigor and transparency of our imaging quantification and statistical analysis.

3. Figure 3G, the image was not focused, and resolution quality needs to be improved.
A: We thank the reviewer for this helpful comment. We have replaced the representative image in Fig. 3G with a clearer image selected from the original dataset under the same experimental condition. Only uniform brightness and contrast adjustments were applied to the entire image, and no local or selective image manipulation was performed. This change improves figure presentation quality and does not affect the quantitative results, statistical analysis, or conclusions.

4. it is not clear how the number of migrated cells was counted to generate the quantitative data.

A: We thank the reviewer for pointing out that the method used to count migrated cells was not sufficiently described in the original manuscript. We have now revised the Methods section and the relevant figure legends to clarify the Transwell migration quantification procedure.

Briefly, after the migration assay, non-migrated cells on the upper surface of the Transwell membrane were gently removed, and cells that had migrated to the lower surface were fixed with 4% paraformaldehyde and stained with crystal violet. For each insert, five randomly selected, non-overlapping fields were imaged under the same magnification and microscope settings. Migrated cells were quantified using ImageJ/Fiji. Images were converted to 8-bit grayscale, a consistent threshold was applied to identify stained cells, and the "Analyze Particles" function was used to count migrated cells. When necessary, touching cells were separated using the watershed function, and the automated counts were visually checked to ensure accuracy.

For statistical analysis, the counts from multiple fields within the same insert were averaged to generate one value for that insert. The values from independent experiments were then used as biological replicates for statistical comparison. Individual microscopic fields were treated as technical subsampling and were not used as independent replicates. We have added this information to the revised Methods section (lines 810-824 and 1051-1056).

We appreciate the reviewer's helpful suggestion, which has improved the clarity and rigor of our migration assay quantification.

5. it is unclear how the LncRNAs are more important in this manuscript, other type of RNAs should be investigated in the manuscript.

A: We sincerely thank the reviewer for raising this important point. We apologize that the rationale for focusing on lncRNA TS2083 was not sufficiently clear in the original manuscript. We did not intend to suggest that lncRNAs are generally more important than other classes of RNA cargo in PEDV-induced sEVs. Rather, our focus on lncRNA TS2083 was based on a phenotype-driven screening strategy.

In our sEV RNA profiling, PEDV infection altered multiple classes of host ncRNAs, including miRNAs and lncRNAs. We first analyzed differentially expressed miRNAs because miRNAs are well-established post-transcriptional regulators and because enrichment analysis of predicted miRNA targets was associated with cell migration and cytoskeletal regulation. This led us to identify miR-190a as a functional mediator of macrophage migration suppression. However, our subsequent functional assays showed that miR-190a did not sufficiently explain the strong inhibitory effect of PEDV-induced sEVs on macrophage phagocytosis. This prompted us to examine additional sEV-associated cargos.

We then analyzed the differentially expressed lncRNAs and identified lncRNA TS2083 based on its enrichment in PEDV-induced sEVs and its association with immune and phagocytosis-related pathways. Functional assays further demonstrated that lncRNA TS2083 contributed to defective macrophage phagocytosis through the ANXA2/F-actin/phagocytic-cup pathway. Thus, lncRNA TS2083 was selected not because lncRNAs were assumed to be more important than other RNA types, but because it provided a mechanistically testable explanation for the phagocytosis defect that was not accounted for by miR-190a.

In response to the reviewer's comment, we have revised the Results section to clarify this stepwise screening logic (lines 214-216, 223-227, 309-310 and 317-322). We have also revised the discussion to acknowledge that PEDV-induced sEVs contain diverse cargos and that other RNA species, such as circRNAs, mRNAs, and additional small RNAs, as well as non-RNA cargos such as proteins, may also contribute to macrophage dysfunction (lines 502-504 and 509-511). A systematic functional screen of all sEV cargo classes was beyond the scope of the current study and will be an important direction for future investigation.

We are grateful to the reviewer for this helpful suggestion, which allowed us to better clarify why lncRNA TS2083 was selected and to more accurately define the scope of our conclusions.

6. Although this manuscript was trying to prove the role of exosomes in the cellular functional assay, the secretome other than exosomes should be included as the control

group in Fig 1H-L, Fig 2, Fig 3, and Fig 5.

A: We sincerely thank the reviewer for this important and constructive suggestion. We agree that, to more rigorously attribute the macrophage-suppressive activity of PEDV-infected epithelial-cell secretome to the sEV fraction, it is important to compare sEV preparations with the non-vesicular secretome remaining after sEV depletion.

In response to this comment, we prepared sEV-depleted conditioned supernatants from MOCK-, WIV-PEDV-, and PEDV-treated epithelial cells and tested their effects on macrophage migration and phagocytosis. The sEV-depleted supernatant from PEDV-infected epithelial cells showed markedly reduced suppressive activity compared with the corresponding total conditioned medium or PEDV-sEV preparations, and its effect was not significantly different from the MOCK or WIV-PEDV controls. These results support the conclusion that the major macrophage-suppressive activity in the PEDV-infected epithelial-cell secretome is associated with the sEV fraction rather than being driven solely by soluble non-vesicular secreted factors (Fig. S5F, G).

We have added these new data to the revised manuscript as a functional control for the sEV attribution experiments. These results also complement the GW4869 experiment in Fig. 3, in which inhibition of sEV release from donor epithelial cells attenuated the suppressive effects of PEDV-infected conditioned medium on macrophage migration and phagocytosis. Together, the purified sEV transfer experiments, the sEV-depleted supernatant control, and the GW4869 inhibition assay provide convergent evidence that PEDV-induced macrophage dysfunction is primarily mediated by sEV release and transfer.

Regarding the specific figures mentioned by the reviewer, Fig. 1H–L was designed to establish that the total secretome from PEDV-infected epithelial cells suppresses macrophage migration and phagocytosis. Fig. 2 was designed to characterize purified sEVs and examine their direct transcriptional reprogramming effects on macrophages. Fig. 3 is the functional attribution step in which purified sEVs, sEV-release inhibition, and now sEV-depleted supernatants are compared. Fig. 5 focuses on the downstream intracellular mechanical mechanism after miR-190a delivery and is therefore not a direct secretome-fractionation assay. Accordingly, we incorporated the sEV-depleted supernatant control into the functional attribution experiments and revised the text and

figure legends to clarify the experimental logic across these figures.

We have also added the preparation method for sEV-depleted supernatants to the Methods section and revised the Results and Discussion accordingly. In the Discussion, we now acknowledge that although the new data support a predominant role of the sEV fraction, soluble non-vesicular factors may still have auxiliary or context-dependent effects and should not be completely excluded (lines 212-213, 196-201, 395-397 and 633-642).

We greatly appreciate the reviewer's suggestion, which has strengthened the causal attribution of the macrophage-suppressive phenotype to PEDV-induced sEVs and improved the rigor of the revised manuscript.

Reviewer #4 (Remarks to the Author):

It is quite innovative that this study investigated exosome-mediated immune evasion mechanisms of porcine epidemic diarrhea virus (PEDV). Overall, the studies were designed well and comprehensive, and the paper was written well. Because only PEDV was studied, the title should be revised to be specific: change “enteric coronavirus” to “PEDV”. Otherwise, the authors need to provide data for at least one more enteric coronavirus. Also, age-dependent disease susceptibility is a characteristic for PEDV. It would be nice to investigate whether such exosomes are generated in adult pigs, which usually show mild clinical signs and no mortality after PEDV infection.

A: We sincerely thank the reviewer for the positive assessment of the novelty, experimental design, and writing of our manuscript. We agree that the original title was overly broad because our study focused specifically on PEDV and did not examine other enteric coronaviruses. Accordingly, we have revised the title to make it PEDV-specific and consistent with the scope of our data.

We also appreciate the reviewer’s important comment regarding the age-dependent susceptibility of PEDV infection. To partially address this age-related question, we performed an additional pilot age-comparison experiment using 30-day-old piglets, which represent an older and less susceptible age group than neonatal piglets, but not adult pigs. In this experiment, PEDV challenge in 30-day-old piglets resulted in limited intestinal infection, as indicated by markedly lower PEDV-N RNA levels and minimal detectable PEDV antigen compared with neonatal piglets (Fig. R1A–D). Consistently, serum-derived sEVs from PEDV-challenged 30-day-old piglets did not show significant enrichment of miR-190a or lncRNA TS2083 and did not significantly suppress macrophage migration or bead uptake (Fig. R1E–H).

We agree that 30-day-old piglets do not represent adult pigs. Therefore, we do not present these data as evidence for adult PEDV infection. Instead, we present them as a pilot age-boundary analysis showing that the sEV-associated macrophage-suppressive phenotype is attenuated when intestinal infection is inefficient in older piglets. These data suggest that the generation of macrophage-suppressive, ncRNA-containing sEVs may depend on efficient intestinal epithelial infection and may vary with host age. Because this pilot experiment was intended to define the age-related boundary of our

conclusions rather than to serve as a main mechanistic dataset, we did not include these data as a main result in the revised manuscript. We acknowledge this limitation and to note that future age-stratified studies, particularly in adult pigs with mild or subclinical PEDV infection, will be needed to determine whether similar sEV-associated ncRNA cargos and macrophage-suppressive activity are generated.

We thank the reviewer for this helpful suggestion, which allowed us to refine the scope of the title and to better define the age-related boundary of our conclusions.

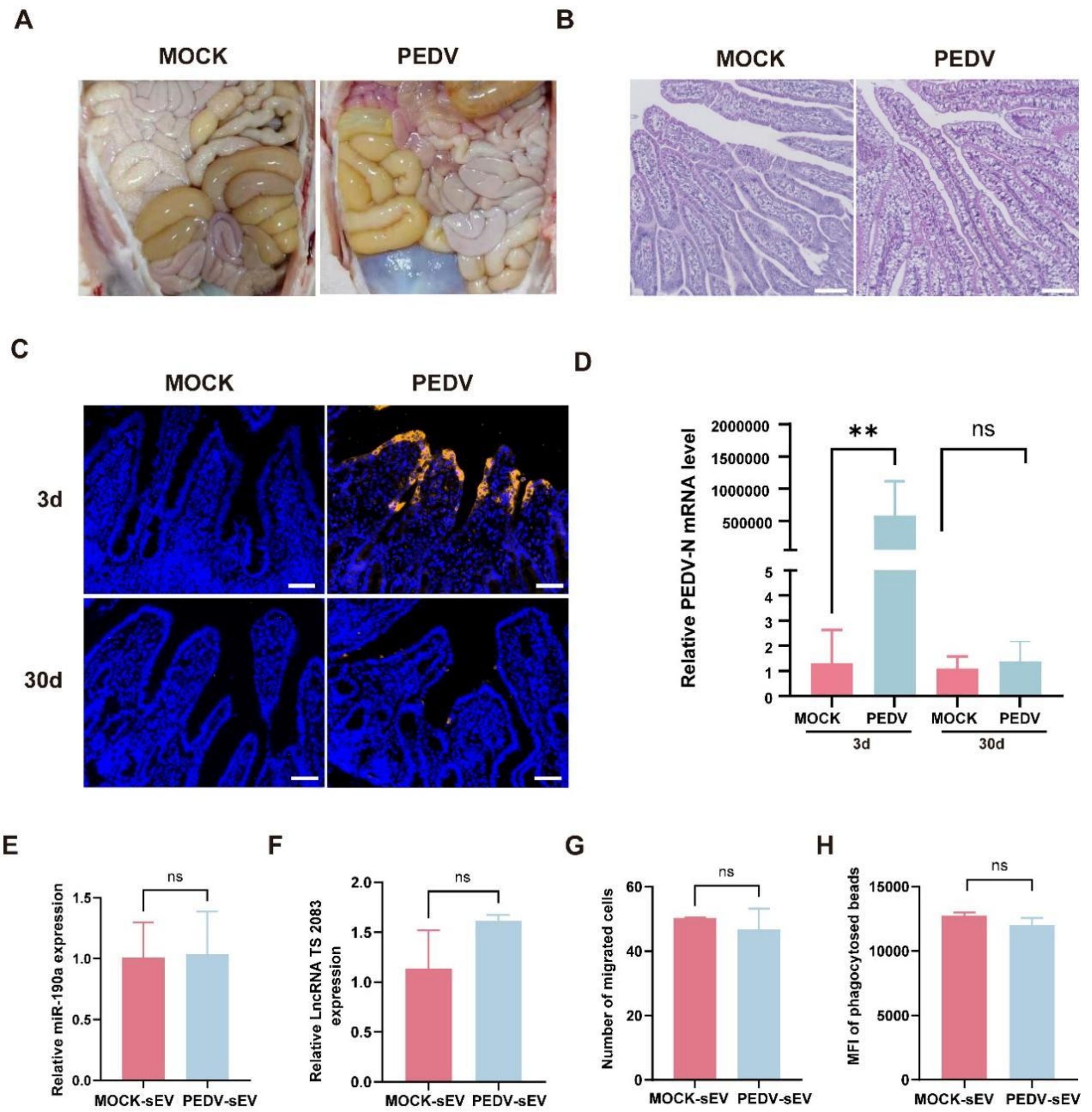

Fig. R1 Evaluation of PEDV infection and serum sEV-associated macrophage responses in 30-day-old piglets. (A) Representative gross appearance of intestinal tissues from 30-day-old piglets after mock or PEDV challenge. (B) Representative hematoxylin and eosin (H&E) staining of jejunal tissues from 30-day-old piglets after

mock or PEDV challenge. Scale bar, 100 µm. (C) Immunofluorescence detection of PEDV antigen in jejunal sections from 3-day-old and 30-day-old piglets after mock or PEDV challenge. PEDV antigen is shown in orange, and nuclei were counterstained with DAPI (blue). Scale bar, 50 µm. (D) RT–qPCR analysis of PEDV-N RNA levels in jejunal tissues from 3-day-old and 30-day-old piglets after mock or PEDV challenge. (E, F) RT–qPCR analysis of miR-190a (E) and lncRNA TS2083 (F) levels in serum-derived sEVs from 30-day-old piglets after mock or PEDV challenge. (G, H) Flow-cytometric analysis of macrophage responses after treatment with serum-derived sEVs from 30-day-old piglets. (G) Quantification of migrated macrophages. (H) Quantification of bead-associated fluorescence, shown as mean fluorescence intensity (MFI).Data are presented as mean ± SD. Statistical significance was determined using unpaired two-tailed Student's *t*-test. ns, not significant; $*P < 0.05$, $**P < 0.01$.

Specific comments:

1. Please spell out all abbreviations when a word first appears in the manuscript.

A: We thank the reviewer for this helpful comment. We have carefully reviewed the entire manuscript and revised all abbreviations so that each term is spelled out at its first occurrence, followed by the abbreviation in parentheses. We have also standardized abbreviation usage throughout the revised manuscript to improve clarity and consistency.

2. Fig. 6H. It seemed that miR-190a not only reduced TMOD3 abundance and disrupted its co-localization with actin fibers but also induced the aggregation of TMOD3. Please describe and explain it.

A: We thank the reviewer for this careful observation and helpful suggestion. We agree that, in Fig. 6H, miR-190a treatment not only reduced the overall TMOD3 signal and decreased its co-localization with F-actin fibers, but also caused the remaining TMOD3 signal to appear more punctate and disorganized. Previous studies have shown that Tmod3 is a pointed-end actin-capping protein that binds actin/tropomyosin and regulates actin dynamics[1]. In non-muscle cells, tropomodulins help stabilize actin–tropomyosin filaments, and disruption of tropomodulin function can destabilize contractile actin structures and shift cytoskeletal organization toward more disordered actin networks[2]. In addition, Tmod3 has been reported to show cytoplasmic localization during mouse oocyte maturation, supporting the view that Tmod3 can display context-

dependent intracellular distribution patterns[3]. Therefore, we interpret the phenotype in Fig. 6H as a loss of organized F-actin–associated TMOD3 distribution with disorganized punctate residual staining, rather than definitive evidence of bona fide protein aggregation. Because we did not perform biochemical assays specifically designed to detect protein aggregation, such as detergent-insoluble fractionation, aggregate-specific staining, or other aggregation assays, we avoided interpreting this punctate staining as protein aggregation. Instead, we have revised the Results to describe this phenotype more accurately as "loss of organized F-actin–associated TMOD3 distribution with disorganized punctate residual TMOD3 staining (298-301, 304-306 and 422-444)." We thank the reviewer for this important observation, which helped us improve the accuracy of our morphological description and interpretation of the TMOD3 staining pattern in Fig. 6H.

3. Please provide the ATCC# or the original resource of your IPEC-DQ cells.

A: We thank the reviewer for this helpful comment. The IPEC-DQ cells used in this study were not obtained from ATCC and therefore do not have an ATCC catalog number. IPEC-DQ is a PEDV-susceptible subclone derived from the porcine intestinal epithelial cell line IPEC-J2 by limiting dilution, as previously described by Zhang et al[4]. In the revised manuscript, we have added the original source, derivation method, culture conditions, and routine quality-control information for IPEC-DQ cells in the Materials and Methods section. We also clarified that the cells were maintained in our laboratory, routinely tested to be mycoplasma-free, and validated for PEDV susceptibility before use. This revision improves the traceability and completeness of the cell-line information (Fig. S3B, E and lines 580-587).

4. P503. "virulent isolates (GenBank: JX002693)" just provides the M gene sequence of the PEDV Zhejiang08 strain. Is there complete genomic sequence available for this strain?

A: We thank the reviewer for carefully pointing out this accession-number issue. We apologize that the PEDV strain used in this study was mistakenly described in the original manuscript as another laboratory-maintained strain, Zhejiang08, and was

incorrectly associated with accession no. JX002693. This was a manuscript-preparation error. The strain actually used throughout this study was the highly virulent PEDV strain CHYJ130330, kindly provided by the Guangdong Haid Institute of Animal Husbandry and Veterinary Medicine. The complete genome sequence of CHYJ130330 has been reported previously[5] and is available in GenBank under accession no. KJ020932. We have corrected the Materials and Methods section and removed the incorrect references to Zhejiang08/JX002693 from the revised manuscript and relevant supplementary materials (lines 576-580). This correction only concerns the reported strain name, source, and accession number, and does not affect the experimental design, data analysis, or conclusions, as the same laboratory virus stock was used consistently throughout all infection, sEV-induction, and in vivo piglet challenge experiments.

5. P527-528, Pigs "were housed individually for 24 h prior to experimentation to allow acclimatization and minimize stress." Did it increase stress since pigs are social animals?
A: We thank the reviewer for this important comment. We agree that pigs are social animals and that individual housing may increase stress. The original wording was inaccurate. In the actual experiments, piglets were not housed individually; after arrival and random assignment, they were housed by experimental group in designated pens, with different treatment groups separated to prevent cross-contamination. The 24-h acclimatization period was used to allow adaptation to the new environment, feeding, and drinking conditions. We have revised the Materials and Methods section accordingly (lines 607-611). The sentence has been changed to: "After arrival at the animal facility, piglets were housed by experimental group in designated pens and allowed to acclimatize for 24 h before experimentation to minimize stress associated with transport and environmental change."

Reviewer #5 (Remarks to the Author):

The work "Exosome-mediated macrophage reprogramming drives mucosal and systemic immunosuppression in enteric coronavirus infection" is about the entry of the enteric alphacoronavirus porcine epidemic diarrhoea virus (PEDV) into epithelial cells and their subsequent production of extracellular vesicles (EVs). These EVs have a particular miRNA/lncRNA consistency, and the authors describe their effect on macrophage function. They suggest that this interaction suppresses pathways that cause the development of the disease, despite upregulation of IFN pathways. The work is technologically extensive, with a lot of data, and has a strong rhetorical foundation for investigation. However, I cannot approve this work for publication without the following revisions to the manuscript.

A: We sincerely thank the reviewer for the careful evaluation of our manuscript and for recognizing the technological breadth, originality, and rationale of the study. We also greatly appreciate the reviewer's constructive comments, which have helped us improve the rigor, clarity, and balance of the manuscript. In response, we have carefully revised the manuscript, clarified several methodological and interpretive issues, and refined the scope of our conclusions to better align with the experimental evidence. The detailed revisions are described in our point-by-point responses below. We believe that these changes have improved the overall quality and rigor of the manuscript, and we address each comment in detail below.

There are some questions on the translatability of this finding towards other species, like humans or mice. Can the authors show any translatability from these models? How much homogeneity of these targeted genes is there compared to other species? Even if it is only *in vitro*, showcasing that these types of dynamics can also play a role in human-related infections would strengthen the work immensely. This work is strong for the observations that the authors have obtained, but this is a clear part of the work that is lacking. Likewise, the authors introduce the economic burden of viruses like SARS-CoV-2 and PEDV, but despite all the knowledge acquired during this publication, there is no experiment that progresses to halting the disastrous effects of PEDV. However, since I am no expert in sus scrofa models, and the authors do show relations *in vivo*, this might be outside the possibilities of the model.

A: We sincerely thank the reviewer for this thoughtful and important comment. We agree that the cross-species translatability of our findings and their potential therapeutic relevance should be discussed more carefully in the revised manuscript.

To address this point, we performed a preliminary validation using PMA-differentiated THP-1-derived macrophage-like cells to examine whether PEDV-induced epithelial sEVs could induce a similar direction of functional change beyond the porcine system. PEDV-sEV treatment reduced migration and phagocytic activity in human macrophages, suggesting that macrophage functional responses to sEV-associated immunoregulatory signals may not be restricted to porcine cells (Fig. S5J, K).

To further evaluate the potential cross-species relevance of the downstream effector pathways, we compared the protein sequences of TMOD3 and ANXA2 among pig, human, and mouse. The analysis showed high conservation of both proteins across these mammalian species, and the results have been added as Supplementary Table S3. These data support the possibility that the downstream cytoskeleton-related effector pathways may have broader relevance. However, we have clarified that the conservation and functional equivalence of the specific PEDV-sEV-associated ncRNA cargos remain to be determined, and we do not claim that the complete PEDV-induced ncRNA cargo mechanism operates in human coronavirus infection.

Regarding the reviewer's point about human-related infections, we agree that testing similar sEV-mediated macrophage-reprogramming mechanisms in human viral infection models would substantially strengthen the translational significance of this work. However, PEDV is a porcine enteric coronavirus, and the current study was designed to define the mechanism of PEDV-induced macrophage dysfunction in its natural porcine host context. We therefore present the human macrophage experiment as preliminary evidence for cross-species macrophage responsiveness to sEV-associated immunoregulatory signals, rather than as direct evidence that the same mechanism occurs during human coronavirus infection. We have revised the Discussion accordingly and moderated broader extrapolations to human viral diseases.

We also agree that therapeutic intervention is an important next step. Although the present study was primarily designed to define the mechanism of PEDV-induced

immune suppression rather than to test antiviral therapy *in vivo*, our results provide several potential intervention points. Inhibition of sEV release, blockade of miR-190a, and restoration of TMOD3 or ANXA2 expression partially or substantially reversed the corresponding macrophage dysfunction phenotypes in our experimental systems. These findings suggest that sEV-associated ncRNAs and their downstream cytoskeletal targets may serve as candidate directions for future intervention studies. Nevertheless, we now explicitly state that *in vivo* therapeutic efficacy, including whether these interventions can reduce PEDV disease severity, viral shedding, or mortality, remains to be determined. We sincerely thank the reviewer for this valuable suggestion, which helped us better define the translational potential, therapeutic implications, and current limitations of our study.

Major/minor revision:

1. The authors stated that they performed scRNA-seq: “Jejunal mucosa (pooled from two piglets) was collected for single-cell RNA sequencing (scRNA-seq)”. And performed subsequent analysis, even adding additional datasets: see “Methods - Processing the single cell RNA sequencing data”

a. I have issues with the experimental design of the generated dataset in this study. We see that samples were pooled prior to generating the data, which causes a loss of replication, meaning that the dataset is now an n=1 per condition – because we are unable to understand the balance between the two replicates and, therefore, the balance of any observation. This does not allow for any statistical analysis at the experimental level.

A: We sincerely thank the reviewer for raising this important concern regarding the experimental design of our scRNA-seq dataset. We apologize for the confusion caused by the inaccurate phrase “pooled from two piglets” in the original Methods section, and would like to clarify that the jejunal mucosal samples used for scRNA-seq were not pooled before library construction. For each condition, samples from two independent piglets were separately processed for single-cell suspension preparation, library construction, and sequencing. Therefore, the scRNA-seq dataset generated in this study contains two independent biological samples per condition, corresponding to two

independent scRNA-seq libraries per group, rather than one pooled sample per group.

We have corrected this description in the revised Materials and Methods section. In addition, the deposited raw scRNA-seq files and sample metadata in the NCBI GEO database (accession: GSE306895) confirm that these libraries were derived from intestinal samples of individual piglets rather than pooled samples. The corresponding sample IDs are GSM9211945 (M_3_1), GSM9211946 (M_3_2), GSM9211947 (V_3_1), and GSM9211948 (V_3_2).

The revised Materials and Methods section now states:

"For scRNA-seq, jejunal mucosal samples from two independent piglets per group were processed separately for single-cell suspension preparation, library construction, and sequencing."

At the same time, we fully agree with the reviewer that, even with two independent biological replicates per condition, the sample size remains limited for robust experimental-level statistical inference. To further address the reviewer's concern regarding cell-level pseudoreplication, we added a sample-aware pseudobulk sensitivity analysis, using each independent piglet sample as the aggregation and analysis unit. The major directional changes observed in the original cell-level analysis were generally consistent with the pseudobulk sensitivity analysis. However, given the limited number of biological replicates, we now interpret these results more cautiously and describe them as supportive transcriptional trends rather than definitive experimental-level statistical proof.

Accordingly, in the revised manuscript, we have reframed the scRNA-seq analysis as an exploratory and hypothesis-generating component, primarily used to identify major transcriptional trends, candidate cell populations, and potential epithelial–immune communication patterns. We no longer present the scRNA-seq data as the sole basis for strong animal-level statistical conclusions. Importantly, the central conclusions of this study do not rely solely on scRNA-seq statistical inference. Instead, the scRNA-seq data guided the identification of macrophage dysfunction and epithelial–immune communication changes, which were subsequently tested by independent functional assays, sEV-transfer experiments, and molecular rescue experiments.

We have revised the Results, Methods, and Discussion accordingly to clarify the sample structure, reduce potential overinterpretation of cell-level analyses, and more explicitly define the limitations of the scRNA-seq component (Fig. S2A-F, lines 106-109, 123-128, 134-136, 401-406, 622-627, 669-692, 752-758 and 1084-1088).

b. The differential abundance analysis of cell types (S1B/C) cannot be performed by an n=1/2.

A: We thank the reviewer for raising this important point regarding cell-type composition analysis. We agree that, with only two independent biological samples per condition, the cell-composition changes shown in Fig. S1B/C should not be interpreted as a formal differential abundance analysis. In the revised manuscript, we now define Fig. S1B/C as descriptive cell-composition summaries, intended to show overall trends in the relative proportions of major cell populations across conditions. Accordingly, we have revised stronger wording such as "decreased" or "increased" to more cautious terms, including relative cell-composition trends and relative enrichment/depletion trends. To better visualize these cell-composition trends, we added ROE analysis, defined as the ratio of observed to expected cells. The ROE results were consistent with the overall direction observed in the original cell-proportion summaries, showing relative enrichment of myeloid populations in the PEDV-infected group and relative depletion of epithelial and secretory cell populations (Fig. S1C, Fig. S1E and lines 106-109). We thank the reviewer for this important suggestion. This comment helped us better define the statistical scope of the cell-composition analysis and improve the rigor of the revised manuscript.

c. I also have an issue with the inflation of observation that ensues in the analysis afterwards (GSEA for GO-terms or KEGG). Aside from the fact that there is no code supplied to go through the details of the analysis, the authors used cells as independent observations in the dataset. This leads to inflated significance, even when correcting for multiple testing – a common mistake with scRNA-seq or other single-cell experiments (PMC6785265). A whole-tissue response from the treatment of the virus per piglet would create dependencies of responses between cells in the tissue.

A: We thank the reviewer for this important and technically insightful comment. We fully agree that cells derived from the same animal are not independent biological observations, and that treating individual cells as independent units in differential expression analysis and downstream GO/KEGG/GSEA enrichment may inflate statistical significance.

In response, we revised both the analysis strategy and the interpretation of the scRNA-seq results. We retained sample identity throughout the scRNA-seq workflow and added a sample-aware pseudobulk sensitivity analysis. In this analysis, cells from each major cell type were aggregated by individual piglet sample to generate sample-level pseudobulk profiles, and each piglet sample was used as the analysis unit. The pseudobulk analysis supported the main directional trends observed in the original cell-level analysis, including increased epithelial antiviral/inflammatory transcriptional programs and reduced macrophage effector transcriptional programs. However, because the number of biological replicates per group remains limited, we now interpret these results as trend-level support rather than definitive experimental-level statistical proof.

Accordingly, we have reduced the emphasis on the statistical significance of cell-level differential expression and GO/KEGG/GSEA enrichment results. These analyses are now described as transcriptional and functional trends, rather than strict sample-level statistical conclusions. For example, wording such as "confirming systematic impairment" has been revised to more cautious wording such as "consistent with reduced transcriptional programs."

To improve transparency and reproducibility, we expanded the Materials and Methods section to include the publicly available software packages, functions, versions, and key parameters used for scRNA-seq preprocessing, quality control, clustering, cell-type annotation, differential expression analysis, sample-aware pseudobulk sensitivity analysis, CellChat analysis, GO/KEGG/GSEA enrichment analysis, and public dataset reanalysis. We also clarified that sample identity was retained throughout preprocessing and downstream analysis (lines 622–692, 694–727, 728–737, 738–751, 752–758, 759–776 and 1084-1088).

We further clarified that no new custom software or algorithm was developed in this

study. All computational analyses were performed using publicly available software packages or online platforms, with versions, key parameters, and analysis settings described in the Methods. The analysis scripts and workflow files used to execute these analyses can be provided to the editors and reviewers upon reasonable request.

These revisions make the statistical scope of the scRNA-seq analyses explicit. We now present this component as exploratory and hypothesis-generating evidence used to identify major transcriptional trends and guide downstream functional experiments, rather than as standalone definitive animal-level statistical inference.

i. To determine the correct response from the piglets, this experiment should be repeated with the appropriate number of repeats to perform statistical analysis. If this is not possible, the analysis should be rewritten to a validation of the results, with clear explanations about the limitations of the analysis and that the results should be interpreted with caution. Alternatively, if the public dataset does have the required amount of experimental observations to perform a correct analysis, start with this and use the experiment as a replication.

A: We thank the reviewer for this important and constructive suggestion. We fully agree that, in an ideal setting, additional biological replicates would provide a stronger basis for experimental-level statistical inference from scRNA-seq data. However, repeating the neonatal piglet infection experiment and generating additional scRNA-seq libraries under the same experimental conditions is not feasible within the current revision period.

Following the reviewer's recommendation, we have therefore revised the scRNA-seq component as an exploratory and hypothesis-generating analysis, rather than presenting it as the primary basis for strict sample-level statistical inference. In the revised manuscript, the scRNA-seq data are used mainly to identify major cellular and transcriptional trends, candidate cell populations, potential epithelial–immune interaction patterns, and macrophage effector-function changes that could be further tested experimentally.

To further evaluate whether the major cell-level transcriptional changes showed consistent directions at the sample level, we added a sample-aware pseudobulk

sensitivity analysis. In this analysis, cells from each major cell type were aggregated by individual piglet sample, and the resulting sample-level profiles were compared with the original cell-level trends. This analysis supported the main directional changes highlighted in the original analysis. Nevertheless, given the limited number of biological replicates per group, we interpret the pseudobulk results as a sensitivity analysis for trend consistency, rather than as definitive experimental-level statistical proof (lines 123-128 and 752-758).

We also retained the reanalysis of the public dataset GSE175411 as independent supportive evidence. However, we have revised the manuscript to present this dataset as supporting concordant trends, rather than as replacing our own dataset as the primary analysis or proving the universality of the mechanism. Accordingly, we have moderated statements in the Results and Discussion that previously implied broad generalization (lines 129-131, 134-136 and 759-776).

Importantly, the central mechanistic conclusions of this study do not rely solely on scRNA-seq statistical inference. The scRNA-seq observations were used to guide subsequent analyses, and the main conclusions are supported by independent macrophage functional assays, sEV-transfer experiments, ncRNA perturbation assays, and molecular rescue experiments. We have revised the Methods, Results, and Discussion to clarify this interpretation and its limitations.

ii. Fig. S1I–K graphs do not correspond with the graphs in the main text, making it difficult to compare them. Please pick one visualisation and use them for both (for instance in CellChat). Also please highlight the overlapping pathways in another colour so that the reader does not have to look for the overlap him/her self.

A: We thank the reviewer for this helpful and specific suggestion. We agree that, in the original manuscript, Fig. S1I–K and the corresponding main-text figures used different visualization formats, which made direct comparison between our in-house scRNA-seq dataset and the public dataset GSE175411 more difficult.

In the revised manuscript, we have reformatted Fig. S1F–H and Fig. S2H using a visualization style more consistent with the corresponding main-text analyses. Specifically, we harmonized the graph type, pathway ordering, color scale, and labeling

strategy so that the two datasets can be compared more directly.

We have also highlighted overlapping pathways or signaling modules between the two datasets using a distinct color or annotation in the revised figure. Pathways detected in both analyses are now visually emphasized, whereas non-overlapping pathways are shown using the standard background color. This allows readers to identify concordant pathways directly without having to manually search for overlaps across panels.

In parallel, we revised the corresponding Results text to describe the public dataset reanalysis as evidence for concordant trends, rather than as definitive proof that macrophage functional suppression is universal across all PEDV infection contexts (lines 129-131). We thank the reviewer for this suggestion, which improved the clarity, readability, and comparability of the public dataset analysis.

2. The authors go on to describe an impaired state of function in the macrophage, based on the analysis of scRNA-seq they derive a loss of migration and phagocytosis which is tested *in vitro* using microscopy. They attribute these effects to signalling derived from epithelial cells after incubation with PEDV.

a. The authors take along a mock virus particle in their experimental design, which seems like an appropriate control.

i. To assure that this control is indeed correct, immune activation markers of inflammation from macrophages should be compared. I understand that the whole point is that the cargo of the virus differs and creates a different type of response, however, since inflammatory immune activation in macrophages change’s functon(e.g. reducing phagocytic capacity via PGE2 and EP4 PMID: 40383415 , which are also involved in migration) it would be good to show that this effect stems from an effect beyond inflammatory activation of the macrophage.

A: We thank the reviewer for this important and insightful comment. We agree that inflammatory activation of macrophages can influence effector functions such as migration and phagocytosis. Therefore, it is important to distinguish whether the macrophage dysfunction observed in our study is caused simply by general

inflammatory activation or involves sEV-mediated functional reprogramming.

To address this issue, we performed additional analyses of inflammatory mediators and macrophage inflammatory-response signatures in our system. First, we measured inflammatory cytokines in conditioned medium from PEDV-infected epithelial cells. The results showed increased levels of IL-6 and TNF-α, whereas PGE2 was not significantly elevated. These data indicate that PEDV infection induces an inflammatory secretory environment, but does not support a prominent PGE2-associated suppressive inflammatory axis in our system.

Second, we analyzed the inflammatory-response features of macrophages treated with conditioned medium from PEDV-infected IPEC-DQ cells (PEDV-CM). GSEA showed enrichment of IL-6/JAK/STAT3 and TNF-α/NF-κB inflammatory transcriptional signatures in PEDV-CM-treated macrophages, suggesting that these macrophages were exposed to and responded to inflammatory cues derived from PEDV-infected epithelial cells. Thus, the macrophages were not inactive or simply hyporesponsive; rather, inflammatory activation and impairment of effector functions such as migration and phagocytosis coexisted under PEDV-CM treatment.

To further evaluate whether the major soluble inflammatory cytokines IL-6 and TNF-α were sufficient to explain the macrophage dysfunction induced by PEDV-conditioned medium, we performed cytokine-neutralization assays. ELISA analysis showed that IL-6 reached approximately 0.8–0.9 ng/mL and TNF-α reached approximately 0.14–0.16 ng/mL in PEDV-conditioned medium. Based on these measured cytokine levels, TNF-α neutralizing antibody was used at 500 ng/mL and IL-6 neutralizing antibody was used at 5 μg/mL before macrophage treatment, with matched IgG controls included under the same treatment conditions. These antibody concentrations substantially exceeded the measured cytokine concentrations in the conditioned medium. The antibody information and treatment conditions have been added to the Materials and Methods section, and the corresponding data are shown in Fig. S4A–F. Neutralization of IL-6 or TNF-α did not markedly restore macrophage migration or bead uptake after PEDV-conditioned medium treatment. These findings indicate that, although PEDV-conditioned medium induces inflammatory cytokine production and activates macrophage inflammatory-response programs, IL-6- or TNF-α-mediated soluble

inflammatory signaling alone is insufficient to account for the migration and phagocytosis defects induced by PEDV-conditioned medium (lines 860-870).

Together with the PEDV-sEV transfer experiments, sEV-release inhibition, and ncRNA rescue assays, these results further support the conclusion that PEDV-induced epithelial sEVs mediate macrophage functional reprogramming in an inflammatory context.

In summary, our data support the interpretation that PEDV infection of epithelial cells generates an inflammatory secretory environment that activates macrophage inflammatory responses, while PEDV-induced epithelial sEVs deliver specific cargo molecules that reprogram macrophage function and suppress immune effector activities such as migration and phagocytosis. We thank the reviewer again for this valuable suggestion, which helped us more clearly distinguish inflammatory activation from functional suppression and strengthened the rigor of our conclusion.

b. Although the images look convincing for migration it would strengthen the story to repeat this work by other techniques that are less prone to technological bias, but rather give an overview of the whole sample.

i. I suggest detaching the cells after migration and counting them through a flow cytometry experiment.

c. Likewise, the phagocytosis of fluorescent particles is measured with counting beads in microscopy

i. Also here, I would suggest using flow cytometry to generate an overview of the whole sample to strengthen the observation.

ii. Minor note on microscopy images, please use magenta and green in all future displays of immunofluorescence images, as this is a colourblind friendly palette.

d. The two comments above (point 2b and 2c) also apply to all other experiments of a similar set up.

A: We thank the reviewer for this constructive suggestion. We agree that microscopy-based assays provide important information on cell morphology, spatial distribution, and phagocytic events, but may also be influenced by field selection.

Therefore, in the revised manuscript, we added flow cytometry-based validation for

the key and most representative macrophage migration, phagocytosis, and F-actin assays. Specifically, flow-cytometric validation was added for the effects of epithelial conditioned medium on macrophage migration and bead uptake (Fig. S3F, G); for purified sEVs, sEV-depleted conditioned medium, GW4869-treated conditioned medium, and THP-1-derived macrophage-like cell validation (Fig. S5D–K); for the functional contribution of sEV-associated miR-190a to macrophage migration (Fig. 4I); for F-actin-associated fluorescence and migration rescue after miR-190a inhibition or TMOD3 overexpression (Fig. 6B, L); for lncRNA TS2083-induced suppression of bead uptake (Fig. 7E); for ANXA2 rescue of the lncRNA TS2083-induced phagocytosis defect (Fig. 8D); and for the effects of serum-derived sEVs from PEDV-infected piglets on alveolar macrophage migration and bead uptake (Fig. 9K, L). These population-level flow-cytometric analyses were consistent with the microscopy-based observations and further support that PEDV-conditioned medium, PEDV-induced epithelial sEVs, and the identified sEV-associated ncRNA axes suppress macrophage migration and phagocytosis (lines 840-859).

For downstream mechanistic experiments, we retained microscopy-based assays as important readouts because they provide spatial information on F-actin remodeling, lamellipodia formation, phagocytic-cup structure, bead localization, and RNA/protein co-localization, which cannot be adequately captured by flow cytometry alone. We have updated the Methods and relevant figure legends to clarify the corresponding flow-cytometric and microscopy-based quantification approaches.

In addition, we appreciate the reviewer’s suggestion regarding colorblind-friendly image display. Where applicable, we have adjusted immunofluorescence and confocal images to a magenta-green color palette and updated the corresponding figure legends. We thank the reviewer again for this helpful suggestion, which strengthened the population-level validation of the functional assays and improved the accessibility of the figure presentation.

3. Next, after testing the PEDV indirectly on macrophages, the authors make an interesting conclusion: PEDV increases extracellular vesicle (EVs, or exosomes) production. Although the direct rational for this direction is lacking (maybe they read it somewhere? –would be nice to find a reference or the observation which led them to

this direction for readability), the authors perform an impressive characterisation in Figure 2.

a. For 2B, it would be informative to add a control (for instance a housekeeping gene from the cells that produced the material) to show that the source of production is at the same level as MOCK. b. 2C. It is unclear how the increase of particles is measured. It would make sense to perform an area under the curve analysis to show repeated experiments in a bar plot.

A: We thank the reviewer for these important and constructive comments. We agree that the rationale for moving from the observation that conditioned medium from PEDV-infected epithelial cells suppresses macrophage function to the analysis of sEVs was not sufficiently explained in the original manuscript. We have therefore clarified this logic in the revised manuscript.

Our focus on sEVs was based on an interesting observation from our initial experiments. On one hand, PEDV-infected intestinal epithelial cells secreted increased levels of inflammatory mediators, including IL-1β, IL-6, and TNF-α, indicating the formation of an inflammatory secretory environment. On the other hand, conditioned medium from these cells markedly suppressed macrophage migration and phagocytosis. This coexistence of inflammatory activation and impaired macrophage effector function suggested that, in addition to classical soluble cytokines and chemokines, PEDV infection might induce other suppressive intercellular communication mediators that contribute to macrophage functional reprogramming.

Because sEVs are important carriers of intercellular communication during viral infection and can deliver immunoregulatory RNA and protein cargos, we further examined changes in epithelial cell-derived sEVs after PEDV infection. We found that PEDV infection increased epithelial sEV release/yield. Moreover, purified PEDV-sEVs directly reproduced the inhibitory effects on macrophage migration and phagocytosis, whereas inhibition of sEV release attenuated these suppressive phenotypes. Therefore, we considered PEDV-induced epithelial sEVs to be important contributors to macrophage functional suppression and proceeded to investigate their molecular mechanisms.

Regarding Fig. 2B, we agree with the reviewer that stronger sEV-associated marker

signals alone should not be directly interpreted as upregulation of these markers. To further clarify this point, we added the GAPDH results from the corresponding donor IPEC-DQ cell lysates that were examined in the original experiment. The GAPDH signals in donor-cell lysates from the MOCK and PEDV-infected groups were comparable, indicating similar donor-cell input for sEV collection. Because the original Fig. 2B was designed to characterize sEV-associated markers rather than donor-cell input controls, this GAPDH result was not shown in the initial submission. Following the reviewer's suggestion, we have now included this result in the revised Fig. 2D and added the corresponding explanation in the "Results" and "Figure legend".

In addition, we performed BCA-based protein-normalized Western blotting of MOCK-sEVs and PEDV-sEVs. Under equal sEV-protein loading conditions, CD63, CD81, and HSP70 did not show a consistent increase in PEDV-sEVs. Together with the donor-cell input control, protein-normalized sEV Western blotting, and NTA particle quantification, these results support the interpretation that the stronger marker signals in Fig. 2B more likely reflect increased epithelial sEV release/yield after PEDV infection rather than uniform upregulation of sEV marker abundance. Accordingly, we have revised the manuscript to avoid describing these data as sEV marker "expression upregulation."

Regarding Fig. 2C, we agree that the original manuscript mainly showed representative NTA size-distribution curves, which did not sufficiently present quantitative differences in sEV production. We have therefore added NTA quantification from independent repeated experiments in the revised manuscript (Fig. 2C) to more directly compare particle concentration/yield between the MOCK and PEDV groups. By presenting both representative size-distribution curves and quantitative statistics from repeated experiments, we now more clearly distinguish between the size-distribution characteristics of sEVs and the increase in sEV release/yield after PEDV infection.

We have revised the Results, Methods, and Fig. 2 legend accordingly (lines 170-185, 643-648, 655-656, 922-930 and 1236-1243). These changes improve the clarity of the rationale for studying sEVs and strengthen the quantitative support for PEDV-induced epithelial sEV release.

4. The authors go on to investigate the effects of these EVs on macrophages. They demonstrate uptake of EVs and show that there are transcriptional and functional changes upon treatment with these EVs.

a. The authors state that there is a difference between PEDV and WIV control in terms of transcriptional activation:

i. How did the authors quantify (and correct for) the EV-load per sample? If PEDV makes more EVs, then a higher response is to be expected. Is it load or a more potent effect due to composition? Also, considering their GW4869 control experiment.

ii. Please put forward a clear hypothesis with regards to this in the text, so that the reader gets a grasp of what is the goal of the experiments. If the hypothesis is composition, consider switching first displaying differences in composition and then effects of function – for the sake of flow.

b. Can the authors consider other ways for visualisation of the effect size difference between induction of transcriptional pathway effects between PEDV and WIV control? This is difficult to read, especially because the comparison is between figures at two different locations.

A: We thank the reviewer for raising this important point. We fully agree that the original manuscript needed to more clearly distinguish the contribution of increased sEV output, that is, the quantity/load effect, from changes in sEV cargo composition, that is, the composition effect, in shaping macrophage transcriptional and functional responses. In response, we have clarified how sEV load was quantified and standardized, refined the comparison between PEDV-sEVs and WIV-PEDV-sEVs, and revised the working hypothesis and presentation order in the manuscript.

First, regarding sEV-dose standardization, we have now clarified in the revised manuscript that, in all downstream macrophage treatment experiments, the amounts of MOCK-sEVs, WIV-PEDV-sEVs, and PEDV-sEVs were standardized based on total sEV protein measured by BCA assay. Thus, macrophages were treated with equal amounts of sEV protein, rather than with equal volumes of conditioned medium. Therefore, the comparison of the transcriptional and functional effects of PEDV-sEVs

and WIV-PEDV-sEVs was performed under standardized sEV dosing conditions. Under these conditions, PEDV-sEVs still induced stronger macrophage transcriptional regulation and stronger suppression of migration and phagocytosis than WIV-PEDV-sEVs. This indicates that the stronger effect of PEDV-sEVs cannot be explained solely by increased sEV release after PEDV infection, but is closely associated with changes in sEV cargo composition (lines 180-183 and 645-648).

To further support this interpretation, we added and emphasized quantitative analysis of key sEV cargos. These results indicate that the stronger macrophage-suppressive activity of PEDV-sEVs is not simply a consequence of applying a larger amount of vesicle-containing conditioned medium, but reflects greater functional potency of the PEDV-sEV preparation under the same standardized sEV-protein input. Together with the sEV RNA-seq results and the subsequent functional validation of the miR-190a/TMOD3 and lncRNA TS2083/ANXA2 axes, these data support the conclusion that PEDV affects epithelial sEV biology at two levels: it increases sEV release/yield and remodels sEV cargo composition, thereby enriching immunosuppressive ncRNA cargos. Thus, the stronger immunosuppressive activity of PEDV-sEVs likely reflects both increased sEV release/yield at the epithelial-secretome level and enhanced functional potency of PEDV-sEVs under protein-normalized treatment conditions, rather than increased sEV output alone (lines 193-196, 216-224 and 309-322).

Regarding the GW4869 experiment, we have further clarified its evidential scope in the revised manuscript. The GW4869 experiment mainly demonstrates that sEV release contributes to the macrophage-suppressive phenotype induced by PEDV-conditioned medium. In contrast, the stronger effect of PEDV-sEVs under equal sEV-protein treatment conditions, together with the enrichment of key ncRNA cargos, supports the contribution of altered sEV composition to macrophage suppression. In other words, the GW4869 experiment addresses whether sEV release participates in the phenotype, whereas the comparison between PEDV-sEVs and WIV-PEDV-sEVs under equal sEV-protein treatment conditions evaluates differences in sEV composition and functional potency (lines 201-203).

Following the reviewer’s suggestion regarding manuscript flow, we also revised the presentation of this section to make the relationship among increased sEV release/yield,

altered cargo composition, and macrophage functional consequences clearer (lines 180-211, 214-224, 230-232 and 309-322). In the revised manuscript, before discussing the macrophage functional effects in detail, we first summarize the cargo-composition differences among MOCK-sEVs, WIV-PEDV-sEVs, and PEDV-sEVs, and clarify that the subsequent functional assays were performed using equal sEV-protein treatment. This allows readers to first understand how PEDV-sEVs differ in composition and then interpret why they produce stronger macrophage transcriptional and functional suppression at the same treatment dose.

Finally, in response to the reviewer's suggestion regarding visualization, we have reorganized the comparison of transcriptional pathway effects induced by PEDV-sEVs and WIV-PEDV-sEVs into a unified display in the revised manuscript (Fig. 2I). The revised figure uses the same pathway order, color scale, and effect-size presentation, allowing readers to directly compare the effects of PEDV-sEVs and WIV-PEDV-sEVs on the same pathways in one location, rather than cross-referencing figures in different sections.

We thank the reviewer for this important suggestion. It helped us better define the quantitative and compositional basis of PEDV-sEV-mediated immunoregulation and improved the clarity of the experimental hypothesis, result organization, and pathway-level visualization in the revised manuscript.

5. Afters identifying these effects, the authors do experiments to understand the composition of the EVs that affect the macrophages. The authors found differences between active and inactive virus and start to screen a selection of the differentially expressed miRNAs finding miR-190a as a repressor of migration.

a. It is unclear what type of analysis was performed to determine the up or downregulation of miRNA, nor what was the rational for selection of migration. Did you use the pathway analysis for this? Then please make this clearer that this was the foundation for selection of the panel in the text.

A: We thank the reviewer for pointing out this important issue. We agree that the original manuscript did not clearly describe how differentially abundant sEV-associated

miRNAs were identified, nor did it sufficiently explain why macrophage migration was selected as the initial functional screening readout.

In the revised manuscript, we have clarified that the differential abundance of sEV-associated miRNAs was determined from RNA sequencing analysis of MOCK-sEVs, WIV-PEDV-sEVs, and PEDV-sEVs. Differential miRNA analysis was performed using edgeR based on normalized miRNA count data, with FDR ≤ 0.05 and $|\log2FC| \geq 1$ used as the screening criteria. We have added these analysis details and selection thresholds to the Methods, Results, and relevant figure legends (lines 218-224, 243-250 and 789-792).

We selected macrophage migration as the initial functional readout for the miRNA mimic panel based on two considerations. First, the predicted target genes of the differentially abundant miRNAs were enriched in cell adhesion, cell migration, and actin/cytoskeleton-related pathways. Second, our preceding functional assays showed that PEDV-sEVs significantly suppressed macrophage migration. Therefore, we prioritized the Transwell migration assay for the initial functional screening of candidate miRNAs. This screen identified miR-190a as the candidate miRNA with the strongest inhibitory effect on macrophage migration (lines 230-232).

These revisions clarify the rationale linking sEV miRNA profiling to the miRNA mimic functional screen.

b. Please highlight miR-190a in figure 4A, so that the reader can find it easier there to see if this migration effect is in accordance with differential expression, but does not seem to play a role in phagocytosis.

A: We thank the reviewer for this helpful suggestion. We agree that highlighting miR-190a in Fig. 4A will make it easier for readers to connect its differential abundance in PEDV-sEVs with the subsequent functional screening results.

Following the reviewer's suggestion, we have highlighted miR-190a in the revised Fig. 4A using a red bold label. We have also updated the figure legend to indicate that miR-190a is a differentially enriched miRNA in PEDV-sEVs and was subsequently identified in the mimic screen as a candidate miRNA with a strong inhibitory effect on macrophage migration (lines 1278-1279).

In addition, we have clarified in the Results that miR-190a is mainly associated with the macrophage migration defect induced by PEDV-sEVs, whereas it did not sufficiently explain the phagocytosis defect in our functional assays. This distinction motivated our subsequent analysis of other sEV-associated cargos, including lncRNA TS2083, to investigate the mechanism underlying phagocytosis suppression.

c. In figure 4B, please show individual experiments as dots, it is now not possible to estimate any other effects.

i. For instance, miR-140b-5p effect size and errorbar look significantly higher than the NC and is also upregulated in PEDV. But there is no way to determine the consistency of this observation right now.

A: We thank the reviewer for this helpful suggestion. We agree that the original Fig. 4B, which presented the miRNA mimic screening results only as bar plots with error bars, did not allow readers to clearly assess the consistency of each candidate miRNA across independent experiments. Following the reviewer's suggestion, we have redrawn Fig. 4B to include individual data points for each independent experiment, while retaining the mean and error bars. In the revised figure, each dot represents one independent experiment, allowing the effects of individual candidate miRNAs on macrophage migration and their reproducibility to be visualized more clearly.

We have also clarified in the Results that Fig. 4B represents a Transwell migration functional screen after miRNA mimic transfection, rather than the differential abundance of miRNAs in sEVs. In addition, we have revised the Results to avoid describing miR-190a as the only candidate miRNA. Instead, we now describe miR-190a as the candidate miRNA with the most pronounced inhibitory effect on macrophage migration in the screen (lines 237-240 and 1278-1281). We thank the reviewer again for this valuable suggestion, which improved the transparency and interpretability of the screening results in Fig. 4.

6. For the AFM part I cannot give any comments, as this is outside my area of expertise. Most noticeable is the fact that the experimental design changes suddenly to an LPS-

induced model, to show inhibition of LPS effect, however, previously this was not the model, nor is there any comparison on a functional level, like TNFa production to gauge how broad the effect of miR-190a is in repressing inflammation.

A: We thank the reviewer for raising this important concern. We agree that the transition from the PEDV-sEV/miR-190a context to the LPS-based AFM/nanoindentation experiments was not sufficiently explained in the original manuscript, which may have made the purpose of this experiment unclear. We have clarified this point in the revised manuscript.

We would like to clarify that LPS was not used in Fig. 5 as a surrogate model for PEDV infection, nor was this experiment designed to evaluate whether miR-190a broadly suppresses inflammatory responses. Rather, LPS was used as a commonly applied macrophage activation stimulus that induces cytoskeletal remodeling and cellular mechanical changes related to migration. Previous AFM-based work showed that LPS-stimulated RAW264.7 macrophages undergo detectable morphological, ultrastructural, and biomechanical changes, including increased adhesion force and stiffness, together with redistribution of intracellular F-actin structures[6]. Therefore, Fig. 5 was designed to establish a reproducible macrophage migration-associated mechanical response and to test whether miR-190a weakens the cellular stiffness, adhesion force, and F-actin remodeling required for macrophage migration. We have clarified this experimental purpose in the revised manuscript and avoided interpreting the LPS-based assay as a PEDV infection model or a general anti-inflammatory assay (lines 257-260 and 1298-1299).

We thank the reviewer for this helpful suggestion. This comment allowed us to more accurately define the scope of Fig. 5 and to improve the logical connection between the LPS-based mechanical assay and the PEDV-sEV/miR-190a/TMOD3 mechanism.

7. Next the authors continue to investigate the effect of actin dynamics by miR-190a, they obtain beautiful images on disrupted dynamics and obtain mechanistic insight into how miR-190a affects the cell.

a. Can figure 6A be repeated with flow cytometry?

A: We thank the reviewer for this helpful suggestion. To provide population-level

validation of the F-actin phenotype shown in Fig. 6A, we performed additional flow cytometry analysis under the same treatment conditions. Macrophages were stained for F-actin, and the overall F-actin-associated fluorescence intensity was quantified. The flow cytometry results showed a reduced F-actin-associated signal after miR-190a treatment, consistent with the decreased F-actin enrichment and impaired lamellipodia formation observed by confocal microscopy.

We retained confocal imaging as the main presentation for Fig. 6A because it provides spatial information on cell-peripheral F-actin enrichment, lamellipodia formation, and cytoskeletal remodeling, which cannot be captured by flow cytometry alone. The new flow cytometry data have been added to the revised manuscript, and the corresponding Methods and figure legend have been updated (Fig. 6B).

b. Please put a white arrow what is the focus point of your image in 6B for the control.

A: We thank the reviewer for this careful suggestion. In the revised Fig. 6B, we have added white arrows to the control images to indicate the leading-edge/lamellipodial region that represents the focus of the time-lapse F-actin remodeling analysis. We have also updated the figure legend accordingly, so that readers can more clearly identify the region of dynamic F-actin remodeling shown in this panel.

c. It is interesting to see that although the miRNA has a strong effect, the actual effect on the gene of interest in rather low, not even a 50% decrease. Can the authors speculate on this?

A: We thank the reviewer for raising this important point. We agree that the relatively modest downregulation of TMOD3 by miR-190a requires further explanation, given the pronounced defects in F-actin remodeling and macrophage migration.

To further support this interpretation, we performed an additional TMOD3 siRNA dose-gradient knockdown experiment using 1 nM and 2 nM of TMOD3-targeting siRNA. Increasing the siRNA dose reduced TMOD3 protein abundance in a graded manner and was accompanied by decreased F-actin-associated phalloidin signals and reduced macrophage migration (Fig. R2). These data indicate that even partial reduction of TMOD3 is sufficient to impair macrophage cytoskeletal remodeling and motility, supporting the interpretation that the miR-190a-induced decrease in TMOD3 can

produce a pronounced migration phenotype.

Previous studies have shown that TMOD3/tropomodulins function as actin filament pointed-end capping proteins that stabilize actin–tropomyosin filaments and maintain the balance between protrusive and contractile actin structures. Impaired Tmod function can destabilize actin–tropomyosin filaments, disrupt stress fiber organization, and induce broader cytoskeletal remodeling[2]. Therefore, we propose that macrophage migration-associated cytoskeletal remodeling is sensitive to changes in TMOD3 abundance. Even partial downregulation of TMOD3 by miR-190a may weaken TMOD3-mediated stabilization of Tpm/F-actin structures, causing local cytoskeletal disorganization to ultimately manifest as suppressed cell migration.

We have also corrected the functional description of TMOD3 in the revised manuscript and now define it as a pointed-end actin-capping protein, which more accurately reflects its known biological function (lines 291-292 and 442-444). We thank the reviewer for this helpful comment, which allowed us to better explain the relationship between partial TMOD3 downregulation and the pronounced migration phenotype.

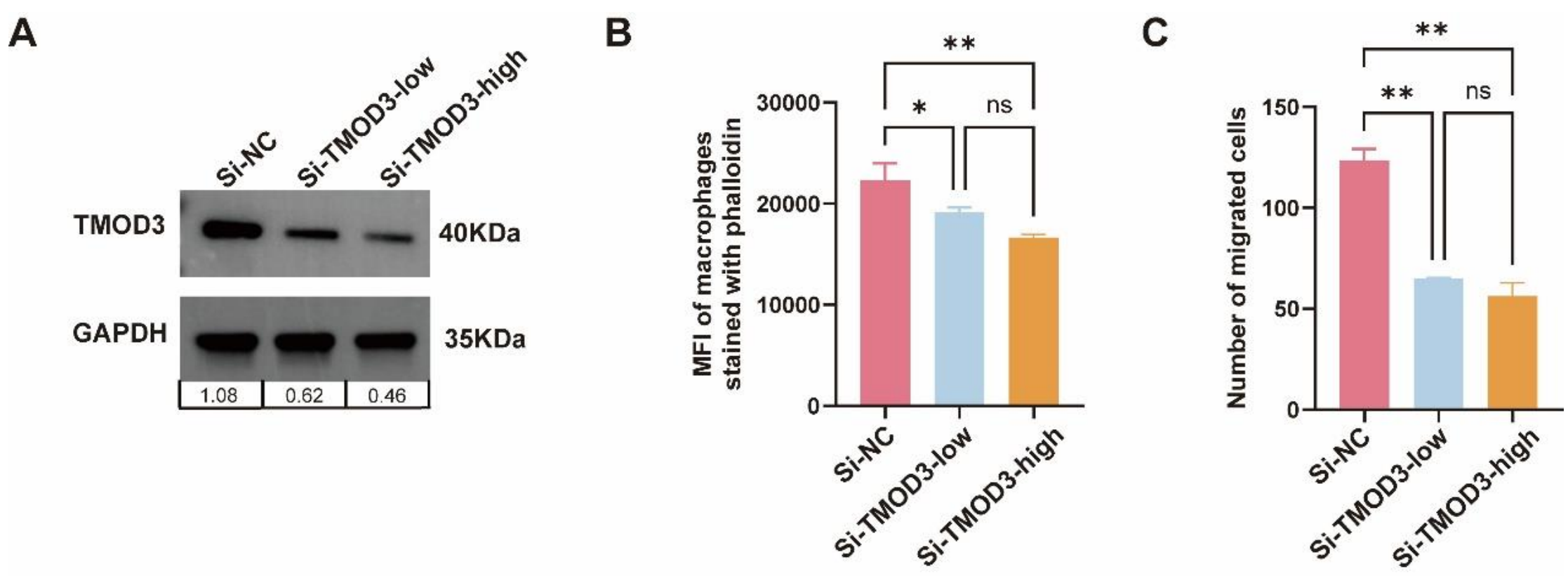

Fig. R2 TMOD3 knockdown impairs macrophage cytoskeletal organization and migration capacity

(A) Western blot analysis of TMOD3 protein expression in macrophages transfected with negative control siRNA or TMOD3-targeting siRNA at high and low concentrations. (B) Immunofluorescence staining of F-actin in macrophages after TMOD3 knockdown, showing the mean fluorescence intensity (MFI) of phalloidin staining. (C) Flow cytometric quantification of migrated macrophages following

TMOD3 silencing. All data are presented as mean ± SD. Statistical comparisons were performed using one-way ANOVA with post hoc multiple-comparison tests. $*P < 0.05$, $**P < 0.01$.

Reviewer #6 (Remarks to the Author):

The editor specifically requested that my review focus on the AFM data. Given my current workload, I have only been able to briefly skim the rest of the manuscript and have therefore restricted my comments to the AFM and nanoindentation experiments.

We sincerely thank the reviewer for the expert and careful evaluation of the AFM and nanoindentation components of our manuscript. We fully understand the reviewer's concern regarding the rigor required for quantitative cell-mechanics analysis, and we agree that the original manuscript did not provide sufficient methodological detail, force-curve presentation, model justification, or interpretive boundaries for the AFM/Chiaro measurements.

In the revised manuscript, we have systematically revised this section. First, we have added key methodological parameters for both Chiaro nanoindentation and AFM force-curve measurements, including probe geometry, spring constant, calibration procedure, measurement environment, loading conditions, indentation depth or fitting range, contact-point determination, force-curve analysis, and the number of cells analyzed. For Chiaro nanoindentation, we clarified the loading and unloading portions of the force–indentation curves used for apparent stiffness and adhesion-force estimation. For AFM force-curve analysis, we clarified the retract-curve fitting region used to estimate local apparent elastic modulus and revised the figure legend accordingly.

We have also revised the interpretation of the mechanical data. In the revised manuscript, the Chiaro and AFM measurements are now described as apparent mechanical parameters used for within-platform relative comparisons among treatment groups measured under the same experimental conditions. We no longer interpret these values as absolute intrinsic material constants of live cells, nor do we directly compare absolute force or modulus values between the Chiaro and AFM platforms.

In addition, we have removed or weakened overinterpretations from the original manuscript, including terms such as "high-stiffness hotspots," "AFM surface mapping," and "front–rear mechanical gradient." Because our AFM data were obtained from force-curve measurements in defined cellular regions rather than from true whole-cell mechanical mapping, the revised manuscript now describes these results more

cautiously: LPS treatment increased local apparent elastic modulus within defined cellular regions, whereas miR-190a treatment attenuated this increase.

Together, these revisions clarify the scope of the AFM/Chiaro data. We now present these measurements as supportive mechanical evidence showing that miR-190a affects macrophage migration-associated cellular mechanics. These data are interpreted together with the F-actin remodeling assays, macrophage migration experiments, and miR-190a/TMOD3 rescue experiments to support the mechanistic conclusion of the study. We address each of the reviewer's specific concerns in detail below.

1. The study aims to demonstrate that exosomal miR-190a suppresses macrophage migration by modulating cellular mechanics, and the authors use both Chiaro nanoindentation and AFM-based force spectroscopy to quantify global and local stiffness as well as adhesion forces. While the hypothesis is interesting, the AFM and nanoindentation data, as presented, fall short of current standards for quantitative cell mechanics.

A: We sincerely thank the reviewer for this important and technically insightful comment. We agree that the original manuscript did not provide sufficient methodological details for AFM measurements, and we have therefore revised the Materials and Methods section to improve clarity, reproducibility, and transparency (lines 990-1014).

In the revised manuscript, AFM force–distance measurements were performed using a BioScope Resolve atomic force microscope (Bruker Corp., USA) in liquid at 37 °C. Live macrophages were maintained in PBS during measurement, and only cells with intact morphology and stable adhesion were selected. A spherical probe with a tip radius of 5.16 μm was used. The cantilever had a length of 115 μm, width of 15 μm, thickness of 580 nm, resonance frequency of 19.8 kHz, and spring constant of 0.102 N/m. The cantilever spring constant was based on the manufacturer-calibrated value for the probe, and deflection sensitivity was calibrated on a clean glass substrate before and after each experiment. Baseline correction was applied to all force curves prior to analysis.

To minimize substrate effects, force curves were collected from raised regions around the perinuclear area, avoiding cell edges and thin peripheral regions. The probe

approach speed was set to 1 μm/s, the trigger force was 1 nN, and the maximum indentation depth was limited to 500 nm. Both approach and retract branches were recorded during measurements. Force–distance data were analyzed using NanoScope Analysis 3.0. The contact point was determined from the transition between the baseline and the rising slope of the approach curve and was uniformly corrected.

For AFM force-curve analysis, representative approach and retract curves were recorded. The local apparent elastic modulus was estimated by fitting the selected region of the retract curve above the baseline using the Hertz–Sneddon model for a spherical indenter, assuming a Poisson's ratio of 0.5. The negative-force adhesion region during retraction was excluded from modulus fitting to reduce the influence of adhesion-related hysteresis. These AFM-derived values were used only for within-platform relative comparisons among treatment groups measured and analyzed under matched conditions.

2. Nanoindentation depths of up to 4 μm raise further concerns, as no correction or discussion of substrate effects is provided. Indentations of this magnitude can be heavily influenced by the stiff underlying substrate, meaning that the reported Young's modulus values may not reflect intrinsic cellular mechanics.

A: We thank the reviewer for raising this important technical concern. We fully agree that when the nanoindentation depth is large relative to the local cell thickness, the underlying rigid substrate may influence the mechanical measurements and cause the calculated Young's modulus to deviate from the intrinsic material properties of the cell.

First, we would like to clarify that the indentation depth of approximately 4 μm refers to the force-controlled measurement process of the Chiaro nanoindentation system, not to the AFM force-curve measurements. The Chiaro nanoindentation experiments were performed in a peak-load-controlled mode, with the maximum load set to 0.03 μN. Under this mode, indentation depth was not manually fixed but was determined by the local mechanical properties of the cell. Therefore, relatively soft cellular regions could show a larger total indentation depth during the full loading process. In contrast, the AFM measurements were performed under independent force-curve conditions, with the maximum indentation depth set to 500 nm. We have clarified these two measurement conditions separately in the revised manuscript to avoid

confusion.

More importantly, for the Chiaro nanoindentation analysis, we did not use the full 0–4 μm force–indentation trajectory for modulus fitting. To better satisfy the small-deformation assumption of the Hertz contact model and to reduce potential substrate-related effects introduced during deeper indentation, we used only the initial shallow indentation region for fitting. Specifically, the fitting region was restricted to the shallow indentation range and did not exceed approximately 16% of the probe radius. Thus, although the probe could reach a relatively large total indentation depth under force-controlled acquisition, the calculation of the apparent modulus was based on the initial shallow portion of the force–indentation curve rather than on the full deep-indentation trajectory.

In addition, based on confocal three-dimensional reconstruction of porcine macrophages (Fig. R3), we observed that adherent macrophages formed a distinct raised cell-body region around the nucleus, where the local cell height reached approximately 16 μm. Because the nanoindentation sites in this study were mainly selected from these perinuclear raised regions, and the maximum indentation depth observed during the full loading process was approximately 4 μm, the indentation depth was lower than the local cell height in these thicker cell-body regions. This reduced the likelihood that the probe directly contacted the underlying rigid substrate. At the same time, as described above, modulus calculation was performed only using the initial shallow indentation region for Hertz-model fitting. Therefore, the selection of thicker perinuclear cell-body regions, together with the shallow-indentation fitting strategy, helped reduce the potential influence of substrate effects on between-group comparisons of apparent modulus.

Nevertheless, we fully agree with the reviewer that even with measurements performed in thicker cell-body regions and with shallow-region fitting, live-cell nanoindentation may still be affected by viscoelasticity, adhesion, cellular structural heterogeneity, and residual substrate-related effects. Therefore, in the revised manuscript, we no longer describe these modulus values as intrinsic material constants of the cells. Instead, we refer to them as apparent modulus or apparent stiffness. These parameters are used only for relative comparisons among treatment groups measured

on the same platform, under the same acquisition conditions, and analyzed using the same fitting strategy.

We have revised the Materials and Methods, Results, and Fig. 5 legend accordingly to clarify the force-controlled acquisition mode of the Chiaro nanoindentation measurements, the shallow-indentation fitting strategy, the selection of measurement regions, and the interpretive scope of apparent stiffness (lines 981-989 and 1299-1308). We thank the reviewer again for this valuable technical suggestion, which helped us more accurately define the applicability of the nanoindentation data and avoid overinterpretation of absolute modulus values.

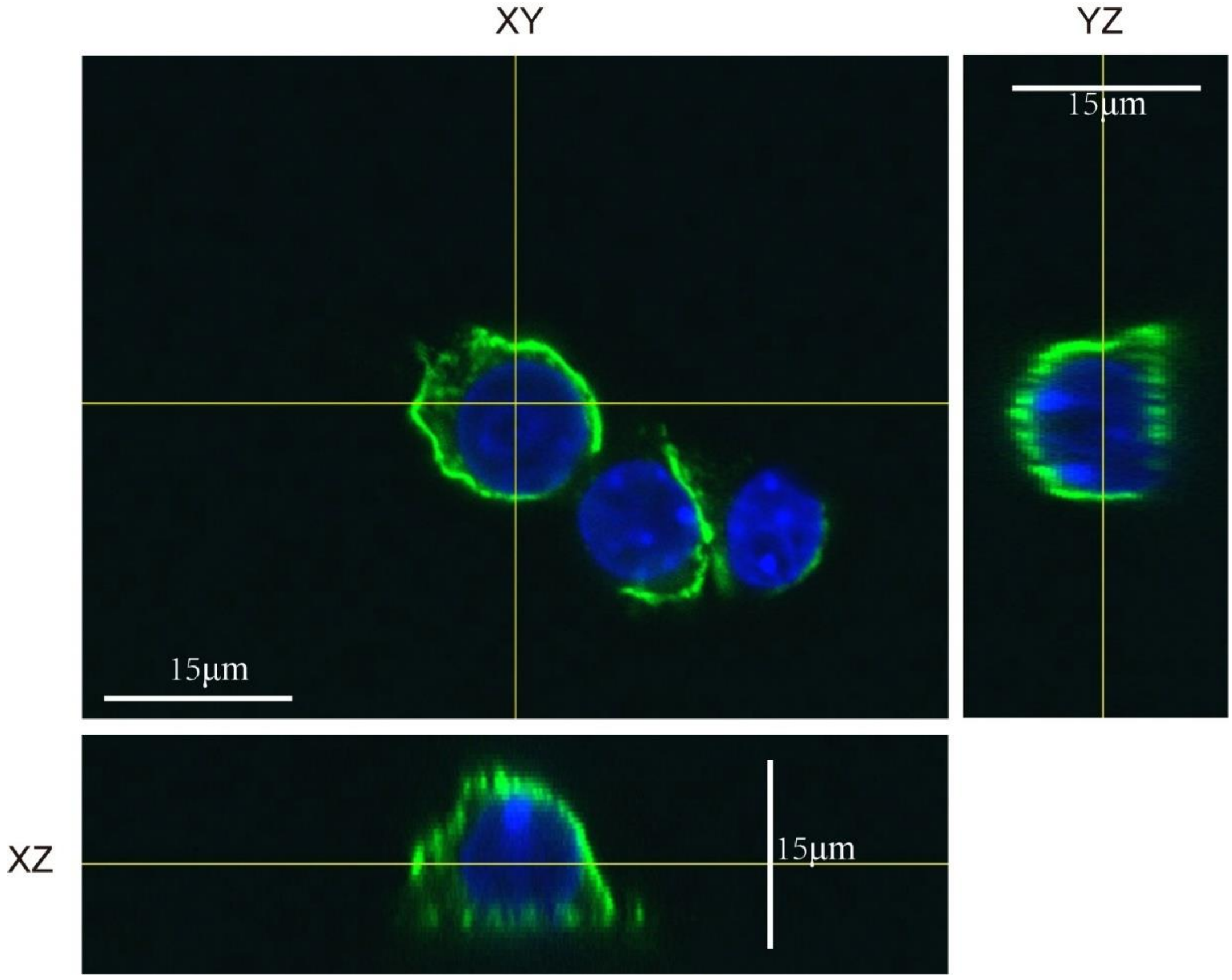

Fig. R3 Orthogonal confocal views of macrophage cell-body architecture.

Orthogonal confocal images showing F-actin organization and nuclear distribution in macrophages. F-actin is shown in green, and nuclei were counterstained with DAPI in blue. The XY plane shows cortical F-actin organization, while the YZ and XZ sections illustrate the raised perinuclear cell-body region used to guide nanoindentation/AFM measurement site selection. Scale bar, 15 μm.

3. There is also a notable inconsistency between probe geometries and applied forces.

Similar force values are reported for both the Chiaro and AFM measurements, despite the use of very different probe radii. This inconsistency complicates direct comparison between techniques and raises questions about the experimental design.

A: We thank the reviewer for raising this important issue. We agree that Chiaro nanoindentation and AFM force spectroscopy differ substantially in probe geometry, contact area, loading mode, trigger conditions, and data acquisition. Therefore, the absolute force values and modulus parameters obtained from the two platforms should not be directly compared across platforms. In the original manuscript, this point was not sufficiently clarified, which may have created the impression that the mechanical parameters obtained from the two techniques were directly equivalent.

Following the reviewer's suggestion, we have revised the Methods and figure legends to more clearly specify the probe type, probe radius, spring constant, and loading conditions used for both Chiaro and AFM measurements. We have also corrected the inconsistency between the AFM probe schematic and the actual probe geometry used in the experiment.

Regarding the reviewer's concern that similar force ranges were observed despite different probe radii, we clarify that the force values shown in representative force curves mainly reflect the loading/trigger range and probe–cell interaction regime under each platform-specific measurement condition. They should not be interpreted as directly comparable absolute mechanical readouts between Chiaro and AFM. Accordingly, in the revised manuscript, we no longer interpret the absolute force or modulus values from the two platforms side by side. Instead, we use each platform only to compare relative changes among treatment groups under its own fixed measurement conditions.

In this study, Chiaro nanoindentation was used mainly to assess cell-scale apparent stiffness and adhesion-related parameters, whereas AFM force-curve measurements were used to assess local apparent elastic modulus within defined cellular regions. Although the two platforms differ in probe geometry and loading conditions, both showed directionally consistent within-platform trends: LPS treatment increased stiffness-related parameters in macrophages, whereas miR-190a treatment attenuated this increase. Thus, the purpose of using both platforms was to provide independent,

directionally consistent mechanical evidence for the same biological phenomenon, rather than to perform cross-platform absolute mechanical quantification.

We have revised the Results, Methods, and Fig. 5 legend accordingly, and now describe these measurements as apparent mechanical parameters used for within-platform relative comparisons (Fig.5G, lines 257-260 and 1298-1315). We thank the reviewer for this helpful comment, which allowed us to avoid overinterpreting mechanical parameters obtained from different measurement platforms.

4. The analysis of AFM force curves also raises concerns. Approach curves are not shown, and the presence of adhesion and hysteresis suggests that a purely elastic Hertz-type model may not be appropriate. Without representative curves and a discussion of the suitability of the chosen mechanical model, it is difficult to assess the validity of the extracted moduli. Furthermore, the identification of high-stiffness "hotspots" and their interpretation as relevant to front–rear mechanical gradients is speculative, as the spatial resolution, indentation depth, and force setpoints used for mapping are not provided.

A: We thank the reviewer for this technically important comment regarding AFM force-curve analysis and the applicability of the mechanical model. We agree that the original manuscript did not sufficiently show representative force curves or describe the fitting branch, contact-point determination, and applicability of the Hertz-type model. We also agree that our previous descriptions of "high-stiffness hotspots" and a "front–rear mechanical gradient" went beyond what the current AFM force-curve data can directly support.

In the revised manuscript, Fig. 5H includes representative AFM force curves from cells under the three indicated treatment conditions. The blue curves correspond to the approach phase, whereas the red curves correspond to the retract phase. The figure legend has been revised accordingly to clarify the interpretation of these curves. Specifically, the shallow indentation region of the retract branch was used for Hertz–Sneddon fitting to estimate apparent elastic modulus for within-group comparisons. We have also described how the contact point was determined and have marked the contact point and fitting region in representative curves (Fig. 5I) to improve the transparency and interpretability of the force-curve analysis.

We agree with the reviewer that adhesion and hysteresis in AFM force curves reflect the viscoelastic and adhesive nature of living cells and may limit the applicability of a purely elastic Hertz-type model. Such adhesion-related effects are commonly observed in live-cell AFM measurements, partly due to cell-surface adhesion molecules and cell–probe interactions[7]. To minimize their influence on modulus estimation, we fitted the rebound curve only in the region above the baseline, excluding the negative-force adhesion region during retraction. The resulting parameters are now described as apparent mechanical parameters, rather than absolute intrinsic material constants. These values are used only to compare relative changes among treatment groups measured under the same experimental conditions and analyzed with the same fitting strategy.

We have also revised the interpretation of the AFM data. Because our AFM measurements were obtained from defined cellular regions using force-curve acquisition rather than from true whole-cell mechanical mapping, we have removed or weakened the previous descriptions of "AFM surface mapping," "high-stiffness hotspots," and "front–rear mechanical gradient." The revised Results now describe these data more cautiously: LPS treatment increased local apparent elastic modulus within defined cellular regions, whereas miR-190a treatment attenuated this increase. We also clarified the relevant acquisition parameters, including the measurement region, indentation/fitting range, and force setpoint, in the revised Methods and figure legend (lines 253-257, 265-269 and 996-1001).

Together, these revisions provide representative force-curve information, clarify how the AFM-derived apparent elastic modulus was estimated, and define the appropriate scope of interpretation for the AFM measurements. We thank the reviewer for this helpful suggestion, which allowed us to improve the presentation, analysis, and interpretation of the AFM mechanical data.

The missing experimental details, potential substrate effects, and ambiguities in force curve analysis make it challenging to evaluate the reliability of the reported mechanical changes. Consequently, the conclusions linking miR-190a to modulation of macrophage migration through mechanical effects are not convincingly supported by the current data.

Overall, we sincerely thank the reviewer for the expert comments on the AFM and

nanoindentation data. We agree that the original manuscript did not provide sufficient methodological detail regarding potential substrate effects, force-curve analysis, and the applicability of the mechanical model, which may have made the interpretive scope of this section unclear. In the revised manuscript, we have added key experimental parameters, representative approach/retract force curves, contact-point determination, the shallow-indentation fitting strategy, and a clearer description of the applicability and limitations of the Hertz–Sneddon model.

We now report Chiaro-derived values as apparent stiffness and adhesion force, and AFM-derived values as local apparent elastic modulus; all values are used only for within-platform relative comparisons among treatment groups measured under matched acquisition and analysis conditions. We no longer interpret the AFM and Chiaro measurements as absolute intrinsic material constants of live cells, nor do we directly compare absolute force or modulus values between the two platforms. We have also removed or weakened potentially overinterpretative terms such as "high-stiffness hotspots," "AFM surface mapping," and "front–rear mechanical gradient." The revised manuscript now describes the AFM results more cautiously as relative changes in apparent elastic modulus within defined cellular regions.

Together with F-actin remodeling, macrophage migration assays, and miR-190a inhibition / TMOD3 overexpression rescue experiments, the revised mechanical data support the conclusion that miR-190a affects macrophage migration-associated cellular mechanics. Importantly, these mechanical measurements are now presented as supportive evidence within a broader functional and molecular evidence chain, rather than as stand-alone or definitive proof of the mechanism.

Reference

1. Parreno, J. & Fowler, V.M. Multifunctional roles of tropomodulin-3 in regulating actin dynamics. *Biophysical Reviews* **10**, 1605-1615 (2018).
2. Kumari, R., *et al.* Tropomodulins Control the Balance between Protrusive and Contractile Structures by Stabilizing Actin-Tropomyosin Filaments. *Curr Biol* **30**, 767-778.e765 (2020).
3. Jo, Y.-J., Jang, W.-I., Kim, N.-H. & Namgoong, S. Tropomodulin-3 is essential in asymmetric division during mouse oocyte maturation. *Scientific Reports* **6**, 29204 (2016).
4. Zhang, Q., Ke, H., Blikslager, A., Fujita, T. & Yoo, D. Type III Interferon Restriction by Porcine Epidemic Diarrhea Virus and the Role of Viral Protein nsp1 in IRF1

Signaling. *J Virol* **92**(2018).
5. Jia, A., Feng, X., Liu, Q., Zhou, R. & Wang, G. Complete Genome Sequence of CHYJ130330, a Highly Virulent Strain of Porcine Epidemic Diarrhea Virus in South China. *Genome Announc* **2**(2014).
6. Pi, J.*, et al.* Detection of lipopolysaccharide induced inflammatory responses in RAW264.7 macrophages using atomic force microscope. *Micron* **65**, 1-9 (2014).
7. Alcaraz, J.*, et al.* Microrheology of Human Lung Epithelial Cells Measured by Atomic Force Microscopy. *Biophysical Journal* **84**, 2071-2079 (2003).

Manuscript: NCOMMS-25-78862B

Title: **Small extracellular vesicle-mediated epithelial–macrophage crosstalk contributes to macrophage dysfunction during PEDV infection**

Reviewer #2 (Remarks to the Author):

The second version after revision addressed my major concerns and current version of results are noteworthy and significant to the related field. The work is original and support the conclusions with enough details and evidence.

A: We thank the reviewer for the positive evaluation of our revised manuscript. No further specific revision was requested.

Reviewer #4 (Remarks to the Author):

The authors have answered my questions and concerns. The revised manuscript is acceptable.

A: We sincerely thank the reviewer for the approval of the revised manuscript.

Reviewer #5 (Remarks to the Author):

The work of the authors of the paper "Small extracellular vesicle-mediated epithelial–macrophage crosstalk contributes to macrophage dysfunction during PEDV infection" has been satisfactory in their rebuttal. All changes, concerning experimental work as well as textual changes, have, in my opinion, significantly strengthened the work and improved its rigour. I only have one final comment to make with regard to the graph visuals in the figure. This will improve interpretability and maximise the authors' opportunity to showcase the scientific rigour and reproducibility they have demonstrated throughout the publication process.

A: We sincerely thank the reviewer for the positive assessment of the revised manuscript and for the helpful final suggestions to further improve data presentation and reproducibility.

1. Could the authors please rework all bar graphs in a way that displays the data per experiment? Since the authors do not disclose this information in the text consistently

as well (n=?). So basically, all summary statistics bar charts, also those in the supplement, should contain more information. For inspiration, I would like to refer the authors to Figure 1 of the following link. (https://rupress.org/jcb/article/219/6/e202001064/151717/SuperPlots-Communicating-reproducibility-and). At the very least, I would appreciate it if the authors showed their mean value per experiment as a dot in all bar graphs. This is an easy conversion in all GraphPad files to do, and will make all data more convincing.

A: We sincerely thank the reviewer for this constructive suggestion. We agree that displaying independent experiment-level data improves the transparency, interpretability, and reproducibility of quantitative results.

In response, we reviewed the quantitative plots in both the main and Supplementary figures and revised the plots used for statistical comparisons to display independent experiment- or animal-level data points. For *in vitro* experiments, technical subsamples collected within the same independently performed experiment were averaged to generate one experiment-level value, and each displayed dot represents one independent experiment. For animal experiments, each displayed dot represents one individual piglet.

The exact *value* of *n* and the experimental unit are now stated in the corresponding figure legends. The Methods, figure legends, and Source Data have been updated accordingly （lines 1044-1049, 1054-1062, 1223-1225, 1237-1238, 1273-1274, 1296-1297, 1328-1329, 1351-1352, 1381-1382 and 1409）.

We thank the reviewer again for this recommendation, which has improved the transparency and rigor of data presentation throughout the manuscript.

2. I am also still missing all scripts that have contributed to their findings with regard to any omic analysis. Having this publicly available will help increase rigour and reproducibility for readers. With these changes, I approve this manuscript for publication.

A: We thank the reviewer for emphasizing the importance of computational reproducibility. We agree that the analysis workflows underlying the omics-related findings should be accessible to readers.

We have deposited the author-accessible R scripts used for the single-cell analyses in a public GitHub repository: https://github.com/apanhui/scRNA_hsa. The repository contains scripts for Seurat-based scRNA-seq analysis, public dataset reanalysis, CellChat analysis, sample-aware pseudobulk differential expression analysis using edgeR, and differential-expression and enrichment analyses. A README file is provided to summarize the software requirements and script organization.

Other omics analyses, including whole-transcriptome sequencing, bulk RNA-seq, proteomics, and platform-based enrichment analyses, were performed using standard commercial service-provider pipelines, publicly available software packages, or web-based platforms. No author-generated custom code or new algorithm was developed for these analyses. The relevant software, platforms, and key analysis parameters are described in the Methods.

A separate Code Availability section has also been added to the revised manuscript to provide access to the analysis scripts（lines 1080-1085）.

Reviewer #6 (Remarks to the Author):

I appreciate the substantial effort made by the authors to address the methodological concerns raised in the previous review. The revised manuscript now contains additional details regarding probe geometry, fitting procedures, contact-point determination, and the limitations of Hertz-type analysis. The authors have also appropriately toned down several overinterpretations. However, after examining the revised Figure 5, I remain concerned that the AFM and nanoindentation data do not yet meet current standards for quantitative cell-mechanics measurements.

A: We sincerely thank the reviewer for the detailed and technically rigorous evaluation of the AFM and nanoindentation-related analyses. These comments helped us reconsider the appropriate scope and interpretation of force-based live-cell measurements in the manuscript.

In accordance with the Editor's explicit instruction, we have completely removed all AFM experiments and AFM-derived conclusions from the revised manuscript. Because the Chiaro nanoindentation measurements served the same auxiliary

phenotypic role as the AFM measurements in the original manuscript, we also removed the remaining Chiaro nanoindentation data and all conclusions derived from these measurements. All corresponding figure panels, Methods descriptions, Source Data sheets, and related textual interpretations have been deleted.

The revised manuscript therefore no longer relies on force-based mechanical measurements as mechanistic evidence. Instead, it focuses on the independently validated PEDV-sEV–miR-190a–TMOD3–F-actin remodeling–macrophage migration axis, supported by macrophage migration assays, F-actin cytoskeletal imaging, flow cytometry-based quantification, direct miR-190a–TMOD3 targeting, and functional rescue experiments (lines11-12, 251-256, 418-429, 497-500, 501-505 and 1411-1419).

1. Presentation of the nanoindentation data

The presentation of the force–indentation curves in panels B,E,I, H remains problematic. The y-axis in panel B is presented in μN which appears unusual. The contact point appears incorrectly positioned in several representative curves. The fitting region shown in panel B is not sufficiently justified and the relationship between the selected fitting range and the actual contact point remains unclear. In panel D, only summary statistics are shown. Given the substantial biological variability expected in live-cell measurements, all individual measurements should be displayed. The panel I is not clear. What is the blue line? Where is the contact point?

A: We thank the reviewer for identifying these presentation concerns. Because all AFM and Chiaro nanoindentation data have been removed, the former force–indentation curves, contact-point and fitting annotations, mechanical summary plots, and associated interpretations are no longer included. These concerns are therefore no longer applicable to the revised manuscript.

2. Quality and interpretability of the representative force curves

The representative curves shown in panels E, H and I exhibit shapes that are difficult to reconcile with standard force-spectroscopy measurements on living cells. The curves display pronounced hysteresis, irregular transitions, and complex force responses that suggest substantial viscoelasticity, adhesion, or other non-Hertzian behaviour. While

such behaviour is not unexpected for living cells, it raises an important issue: it becomes unclear whether these curves are suitable for extracting a meaningful elastic modulus using a Hertz–Sneddon framework.

In particular: The representative AFM curves shown in panel H do not resemble force curves typically used for reliable modulus extraction.The manuscript does not provide sufficient evidence that the selected fitting regions correspond to a regime where Hertz-type assumptions are reasonably valid.

A: We thank the reviewer for raising this concern. All representative force curves, Hertz–Sneddon fitting, and stiffness, modulus, and adhesion analyses have been removed from the revised manuscript. No conclusion in the revised manuscript is based on these measurements or model-derived parameters.

3. Contact-point determination remains unclear

Although the authors state that the contact point has been indicated in panel I, the procedure remains insufficiently transparent. The determination of the contact point is one of the most critical steps in modulus extraction, particularly for soft and highly adhesive biological samples. From the figure, it is not obvious how the contact point was identified, nor how sensitive the resulting modulus values are to this choice.

The figure would benefit from a much clearer illustration of: the raw force curve, the baseline, the chosen contact point, the fitting interval, and the resulting model fit.

A: We thank the reviewer for emphasizing this issue. Contact-point determination and fitting-interval selection are no longer part of the revised study because all AFM and Chiaro nanoindentation analyses have been removed. The associated figure panels, Methods descriptions, Source Data sheets, and interpretations have also been deleted.

4. Validity of the modulus values shown in panel J

Most importantly, I remain unconvinced that the curves presented in panels H and I support reliable extraction of an elastic modulus. The representative curves appear highly dominated by adhesion and hysteretic effects and do not display an obvious region where a simple Hertz–Sneddon model would provide a physically meaningful description of the cell response. Consequently, the values reported in panel J should be

interpreted with extreme caution. Based on the representative curves provided, it is difficult to assess whether the calculated apparent elastic moduli reflect genuine mechanical differences between groups or are instead influenced by fitting choices, contact-point selection, adhesion effects, or other model-dependent artefacts.

A: We thank the reviewer for this concern. All AFM-derived modulus values and nanoindentation-derived mechanical parameters have been removed. The revised manuscript no longer reports apparent stiffness, adhesion force, elastic modulus, Young's modulus, or any Hertz–Sneddon-based parameter, and no conclusion relies on such measurements.

5. Cell-height argument

Finally, I remain unconvinced by the statement that the perinuclear cell-body region reaches approximately 16 μm in height. The orthogonal confocal views provided do not clearly support this value, and no quantitative height analysis is presented. Therefore, the possibility of substrate contributions during indentation cannot be fully excluded.

A: We thank the reviewer for this concern. We agree that the previous images did not provide sufficient quantitative support for assigning a precise cell-height value and did not fully exclude substrate effects. We therefore removed the numerical cell-height statement, the associated figure, and all related interpretations. Because all force-based analyses have been removed, cell-height estimates are no longer used to support any conclusion in the revised manuscript.

While the authors have substantially improved the methodological description and appropriately moderated the interpretation of the mechanical measurements, the representative force curves themselves remain difficult to interpret and do not convincingly demonstrate that reliable stiffness or elastic modulus values can be extracted. As a result, the mechanical component of Figure 5 remains, in my view, the weakest part of the manuscript and does not yet provide robust support for the proposed mechanistic conclusions.

A: We thank the reviewer for this overall assessment. In response to the Editor's instruction and the reviewer's concerns, we have removed the entire force-based

mechanical measurement component from the revised manuscript, including all AFM and Chiaro nanoindentation measurements and related interpretations. Accordingly, the mechanical component of the former Figure 5 is no longer present, and no mechanistic conclusion in the revised manuscript relies on stiffness, elastic modulus, adhesion force, or other force-based mechanical parameters. The revised manuscript now focuses on the independently validated PEDV-sEV–miR-190a–TMOD3–F-actin remodeling–macrophage migration axis.
